# Hospital Discharge Planning—An Investigation of Outcomes and Interventions

**DOI:** 10.1111/1475-6773.70060

**Published:** 2025-10-23

**Authors:** Lena Imhof, Robin Heber, Kai Svane Blume, Jonas Schreyoegg, Vera Winter

**Affiliations:** ^1^ Hamburg Center for Health Economics University of Hamburg Hamburg Germany; ^2^ Schumpeter School of Business and Economics University of Wuppertal Wuppertal Germany

**Keywords:** continuity of care, discharge interventions, hospital discharge planning, outcomes, strength of evidence, transitional care

## Abstract

**Objective:**

To provide a comprehensive overview of the different types of hospital discharge planning (DP) interventions and outcomes examined in systematic reviews, and to assess the strength of evidence (SoE) for the associations between DP and these outcomes.

**Study Setting and Design:**

Umbrella review (“review of systematic reviews”).

**Data Sources:**

We searched five databases (PubMed, CINAHL, Web of Science, Cochrane, and Business Source Complete) from inception through February 2024 for systematic reviews examining associations between hospital DP and various outcomes. We conducted backward and forward citation searches to identify additional systematic reviews. Altogether, these searches yielded 1817 records, of which 34 met the inclusion criteria. We assessed the methodological quality of the included reviews using the AMSTAR 2 tool, summarized DP intervention types and the reviews' subgroup analyses narratively, and evaluated the SoE for 19 outcomes using a recently developed method.

**Principal Findings:**

We identified 20 distinct DP intervention types which we grouped into six intervention categories. Patient education was the most frequently investigated type. We rated SoE as high for five outcomes, moderate for eight, and low for six. We found the strongest evidence for associations between hospital DP and reduced readmissions, fewer medication discrepancies, and greater patient satisfaction. Evidence for associations with quality of life, emergency department visits, mortality, and overall patient health, however, was weak or lacking. Our synthesis of the reviews' subgroup analyses indicated that the effects of hospital DP varied across patient populations and intervention types. Overall, the most effective interventions appeared to be high‐intensity, bundled programs, incorporating medication‐related interventions and follow‐ups, particularly for reducing readmissions.

**Conclusion:**

This umbrella review synthesizes evidence on associations between hospital DP and various outcomes. The findings support the development of tailored DP strategies and point to research gaps. Future studies should prioritize standardizing intervention definitions, outcome measures, and subgroup classifications, and investigate unexamined causal mechanisms.

**Summary**

- What is known on this topic
  ○ Previous literature has reported mixed results regarding the effectiveness of hospital discharge planning (DP).
  ○ Despite extensive research, there is no consensus on which DP practices are optimal or on the impact of DP on healthcare outcomes.
- What this study adds
  ○ We assessed the strength of evidence for associations between hospital DP and 19 outcomes, rating it as high for five, moderate for eight, and low for six outcomes.
  ○ The strongest evidence was found for associations with reduced readmissions, fewer medication discrepancies, and greater patient satisfaction. Evidence for associations with quality of life, mortality, emergency department visits, and patient health was weak or lacking.
  ○ Future reviews and meta‐analyses should standardize the definition of interventions, outcomes, and subgroups and statistically compare effect sizes to strengthen the evidence base.

## 1 Introduction

Transitions between inpatient and outpatient care are critical points at which errors in discharge processes or gaps in continuity of care can place patients at risk [1]. Yet in many health systems, ensuring safe and efficient discharge processes has become increasingly difficult due to rising patient volumes, shorter lengths of hospital stay (LoS), and growing cost pressures [2, 3, 4].

In response, many hospitals have implemented discharge planning (DP) interventions: structured processes designed to facilitate a safe and coordinated transition from hospital to outpatient care and to prevent adverse outcomes after discharge [3]. These interventions commonly include some combination of early assessment of patient needs, individualized discharge plans, medication reconciliation, patient education, and close communication with primary care providers and community‐based services.

A growing body of literature, including primary studies and literature reviews, has examined the relationship between hospital DP interventions and patient outcomes. Much of this research, including previous umbrella reviews [3, 5, 6, 7], has focused on hospital readmissions, likely due to factors such as financial incentives, data availability, and the policy relevance of this outcome (e.g., the Hospital Readmissions Reduction Program [HRRP] in the US [8]). While these reviews provide some evidence that DP may reduce readmission rates [3, 5, 6, 7], relatively little is known about its potential effects on other outcomes, such as patient health status, medication‐related outcomes, or patient satisfaction.

Moreover, existing reviews have rarely applied consistent classifications when synthesizing evidence on DP interventions. For example, Straßner et al. [6] provided a pragmatic narrative description of intervention characteristics, while another umbrella review [3] employed two different categorization schemes: one distinguishing between pre‐ and post‐discharge interventions, and the other using a four‐part categorization proposed in a previous review [9].

As a result, there is still no comprehensive overview of which types of DP interventions and outcomes have been studied, nor clear evidence on whether effects or associations are consistent across outcomes and subgroups.

To address these gaps, we conducted an umbrella review with three aims: (1) to provide a comprehensive overview and categorization of DP intervention types; (2) to compile the outcomes examined in systematic reviews and assess the strength of evidence (SoE) for associations between DP and these outcomes; and (3) to examine whether these associations varied across factors such as the types of interventions the reviews focused on or population characteristics.

In contrast to the broader concept of transitional care, which encompasses time‐limited services supporting continuity of care for patients transitioning between care settings not restricted to hospital stays [1], we focus specifically on hospital‐based DP to narrow the scope of analysis.

## 2 Methods

A protocol for this umbrella review was published in PROSPERO (CRD42024524726). We followed the Preferred Reporting Items for Overviews of Reviews (PRIOR) guidelines (Appendix S1), which were specifically developed for umbrella reviews of healthcare interventions [10].

### 2.1 Search Strategy

We searched PubMed, CINAHL, Web of Science, Cochrane, and Business Source Complete from database inception through February 2024 to identify systematic reviews that conducted narrative syntheses, meta‐analyses, or both. We also manually screened the reference lists of included reviews (i.e., backward searching) and papers citing the included reviews (i.e., forward searching). Our search strategy (see Appendix S2) combined terms related to discharge planning, healthcare setting, and the relationship between DP and healthcare outcomes. The search strategy was developed in collaboration with a medical librarian. To ensure practical relevance and capture variations in terminology, we also consulted DP practitioners, including physicians and nurses.

### 2.2 Selection Criteria and Screening Process

We included systematic reviews that (1) were peer‐reviewed and synthesized evidence on the relationship between hospital DP and quantitative outcomes on quality of care, (2) focused on DP interventions initiated during the hospital stay, and (3) compared DP with usual care.

We excluded reviews focusing on psychiatric patients, palliative patients, or children. Reviews that examined early discharge interventions aiming to reduce length of stay (LoS) were also excluded because these interventions are often not directly comparable to standard DP procedures. Additionally, we excluded reviews that addressed only post‐discharge interventions, as well as primary studies, qualitative studies, and reviews including fewer than five primary studies. Detailed inclusion and exclusion criteria are provided in Appendix S3. When the eligibility of a review was unclear, we required at least two‐thirds of its primary studies to fulfill the inclusion and exclusion criteria outlined above.

Two reviewers (LI, RH) independently screened the titles and abstracts of all identified records for eligibility. Records deemed potentially relevant were then subjected to full‐text screening by the same reviewers. Disagreements at either stage were resolved through discussion and consensus with a third reviewer (VW or KB).

### 2.3 Assessment of Methodological Quality

We appraised the methodological quality of the included reviews using the AMSTAR 2 tool [11]. To adjust the appraisal criteria to our research questions, we made slight modifications to the standard tool (see Appendix S4). Two authors (LI, RH) independently appraised each review. If needed, disagreements were resolved through discussion with a third researcher (VW or KB).

Following the approach of Blume et al. [12], each AMSTAR 2 item was rated 0 if the criterion was not met, 0.5 if partially met, and 1 if fully met. We calculated quality scores at the review level as the average across all items and categorized each review into one of three quality levels: high (score > 2/3), moderate (score ≥ 1/3 but ≤ 2/3), or low (score < 1/3). To assess the sensitivity of the scoring‐based classification, we applied a set of item weights reflecting the importance of each AMSTAR 2 item with respect to our research questions. However, sensitivity analyses using weighting schemes adopted from Blume et al. [12] indicated no meaningful effect on the results. We therefore report unweighted scores.

### 2.4 Data Extraction and Synthesis

Two researchers (LI, VW) independently extracted the following data from each review using a self‐designed Excel template: general review characteristics, description of DP interventions, reported outcomes, and findings from main and subgroup analyses.

#### 2.4.1 Outcome Synthesis and Strength of Evidence (SoE) Assessment

Reported outcomes from each review were extracted, summarized, and synthesized across reviews (see Appendix S5). For instance, we grouped various physical, functional, and mental health outcomes under the term *Health/functional status* and different types of satisfaction under *Patient satisfaction*. The outcome *Post‐discharge healthcare utilization* comprised outpatient follow‐up care (e.g., visits to primary care providers) and community services. Readmission outcomes were grouped according to commonly distinguished timeframes (30‐day, 90‐day, 180‐day, and 1‐year), where reported.

From each review, we extracted information on the reported associations for every outcome, including whether a finding was statistically significant, its effect direction, and whether it was presented as a pooled estimate or part of a narrative summary. This information formed the basis for our SoE analysis.

**FIGURE 1:**
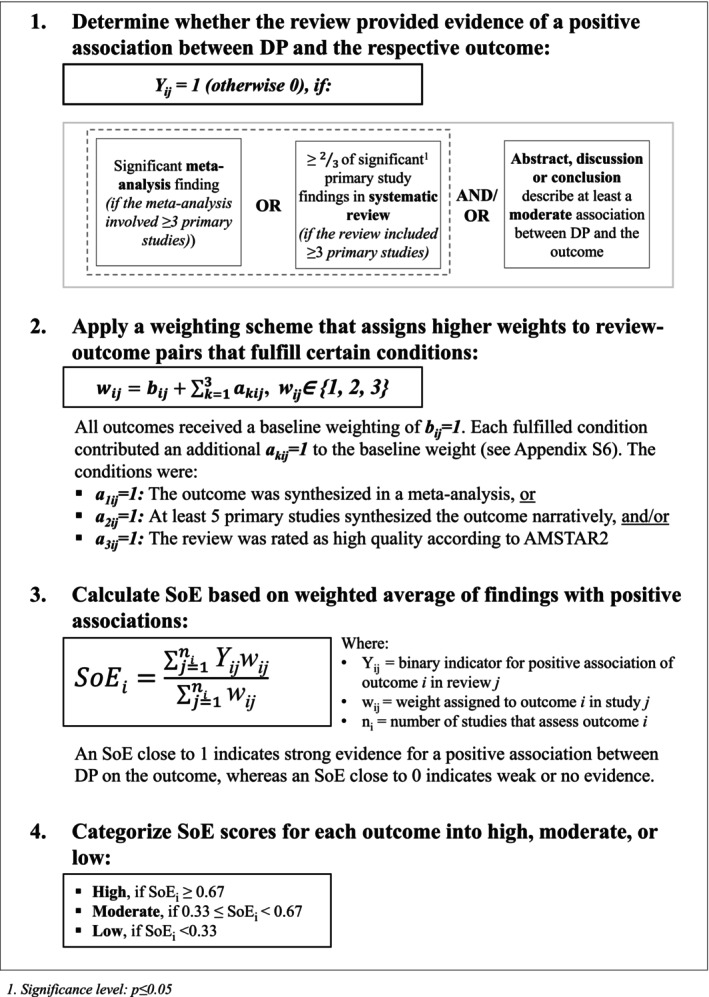
Four‐step methodological approach for assessing the strength of evidence (SoE) for the potential association between hospital discharge planning (DP) and outcomes across the included systematic reviews.

Building on the approach proposed by Blume et al. [12], we assessed the SoE for each outcome in a four‐step approach (see Figure 1). Outcomes were included in the SoE analysis only if they appeared in at least two systematic reviews and in at least 10 primary studies across those reviews.

In step 1, for each review–outcome pair, we evaluated whether the review provided evidence of a positive association between DP and the outcome. This was the case if the review met at least one of three predefined criteria (*Y*_*ij*_ = 1) (see Figure 1, step 1). Otherwise, the pair was coded as showing no such evidence (*Y*_*ij*_ = 0). This binary classification indicates whether the review supported the existence of an association but does not necessarily imply statistical significance in the formal sense. In step 2, to account for variation in methodological quality and synthesis type (meta‐analysis vs. narrative synthesis), we applied a weighting scheme that assigned higher weights to review–outcome pairs that met certain criteria (see Appendix S6). In step 3, we calculated the SoE for each outcome as the weighted average of the binary classifications from step 1 (*Y*_*ij*_ ∈ {0,1}) using the corresponding weights (*w*_*ij*_ ∈ {1,2,3}). Lastly, in step 4, we categorized the resulting SoE for each outcome into three levels: high (SoE ≥ 2/3), moderate (SoE ≥ 1/3 and < 2/3), and low (SoE < 1/3).

Two reviewers (LI and RH) independently assessed the SoE for each outcome. Discrepancies were resolved through discussion. To assess the robustness of our SoE estimates, we conducted sensitivity analyses by varying key assumptions and thresholds used in the SoE calculation (see Appendix S12).

#### 2.4.2 Intervention and Subgroup Synthesis

We extracted information on the hospital DP interventions described in the included reviews. These interventions were implemented in diverse hospital settings and often combined different components, which were sometimes delivered in isolation and sometimes bundled together. To enable comparison across reviews, we synthesized these DP components into 20 distinct intervention types (e.g., patient education, medication reconciliation, follow‐up calls). We then grouped these intervention types into five broader categories (*Preparation*, *Education*, *Medication*, *Coordination*, and *Follow‐up*), similar to the classification approach from Burke et al. [13]. Although our focus was on hospital‐based DP, we included intervention types with components that extended into the postdischarge period if these were initiated during the hospital stay and constituted only a minor part of the overall DP intervention.

For each review, we also documented whether subgroup analyses had been conducted, how they were performed, which subgroups were examined, and what results were reported. We defined subgroup analyses as narrative or statistical comparisons of effects across subgroups based on features such as intervention type, patient population, or study design. We then inductively assigned these subgroup analyses to thematic categories and synthesized the findings narratively.

We were particularly interested in the effects reported for distinct intervention types but also considered the effects of bundled interventions where available. However, most reviews did not provide results specific to intervention types, often due to poor descriptions in the primary studies and/or high heterogeneity across interventions. As a result, a formal SoE analysis at the level of individual intervention types was not feasible. Instead, we synthesized subgroup findings narratively at the intervention type level and also classified reviews that focused solely on one intervention type (e.g., education or medication reconciliation) as subgroup analyses since their study selection inherently reflects a specific subgroup. To summarize findings, we counted how often a positive association or no association between an outcome and an intervention type was reported.

## 3 Results

### 3.1 Screening Results and Inclusion of Reviews

**FIGURE 2:**
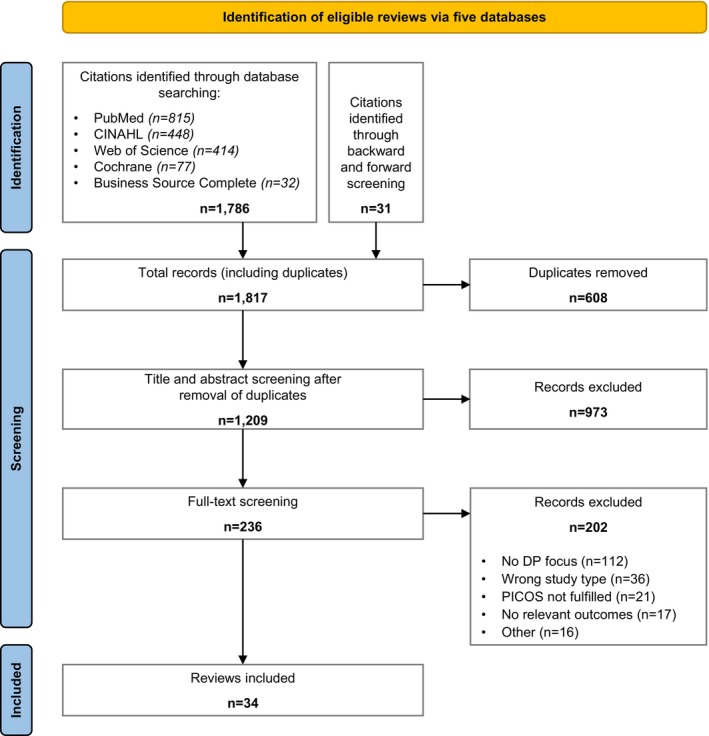
PRISMA flowchart.

We identified 1786 records through database searches and an additional 31 records through backward and forward screening. After removal of duplicates, 1209 unique records remained for title and abstract screening. Of these, 973 were excluded for not meeting the inclusion criteria. The remaining 236 records were retrieved for full‐text screening, resulting in 34 systematic reviews that met the final inclusion criteria. The screening and selection process is summarized in the PRISMA flowchart (Figure 2). Reasons for full‐text exclusions are provided in Appendix S7.

### 3.2 Description of Reviews

Table 1 summarizes the main characteristics of the 34 included reviews. Additional details are provided in Appendix S8. The reviews were published between 2003 and 2023. More than half (*n* = 19) included a meta‐analysis, and the remainder presented narrative syntheses. Most reviews focused on transitional care or DP (*n* = 13). Others targeted specific intervention types, such as medication‐related interventions (*n* = 9), patient‐ and family‐centered care (PFCC) (*n* = 4), or discharge education (*n* = 2). A smaller subset examined broader interventions such as heart failure management programs (*n* = 2), strategies to reduce readmissions (*n* = 1) [39] or programs to improve access to care (*n* = 1) [41].

**TABLE 1:** Characteristics of included reviews.

Author		Review focus	Study design	No. of primary studies	Intervention category					Outcomes^a^	Quality assessment
					Preparation	Education	Medication	Coordination	Follow‐up		
Albert et al. [14]		TC/DP	SR	23			*x*	*x*	*x*	ED visit^b^; HC utilization^b^; Health/Functional status; Patient knowledge; QoL^b^; Readmission^b^	Low [0.17]
Allen et al. [15]		TC/DP	SR	12		*x*	*x*	*x*	*x*	HC utilization^b^; Patient satisfaction^b^; QoL; Readmission^b^	Moderate [0.50]
Fønss Rasmussen et al. [16]		TC/DP	SR	11	*x*	*x*	*x*	*x*	*x*	Readmission^b^	High [0.83]
Gonçalves‐Bradley et al. [17]		TC/DP	MA	33	*x*	*x*	*x*	*x*	*x*	LoS^b^; Med. adherence; Med. error; Mortality^b^; Patient satisfaction^b^; Readmission^b^	High [0.93]
Lee et al. [18]		TC/DP	MA	21	*x*	*x*	*x*	*x*	*x*	Health/Functional status^b^; Mortality; QoL; Readmission^b^	Moderate [0.37]
Leithaus et al. [19]		TC/DP	SR	17	*x*	*x*	*x*	*x*	*x*	ED visit^b^; Readmission^b^	Moderate [0.38]
Mabire et al. [2]		TC/DP	MA	13	*x*	*x*	*x*	*x*	*x*	LoS; QoL; Readmission	Moderate [0.60]
Park et al. [20]		TC/DP	MA	9		*x*			*x*	ED visit; Health/Functional status^b^; QoL^b^	Moderate [0.60]
Rennke et al. [21]		TC/DP	SR	47		*x*	*x*	*x*	*x*	ADEs; ED visit; Readmission	Moderate [0.50]
Stamp et al. [22]		TC/DP	SR	20		*x*	*x*	*x*	*x*	QoL^b^; Readmission^c^	Low [0.25]
Tomlinson et al. [23]		TC/DP	MA	24	*x*	*x*	*x*	*x*	*x*	Med. error^b^; Mortality; QoL^b^; Readmission^b^	Moderate [0.67]
Tyler et al. [24]		TC/DP	MA	126		*x*	*x*	*x*	*x*	QoL; Readmission^c^	Moderate [0.50]
Verhaegh et al. [25]		TC/DP	MA	26	*x*	*x*		*x*	*x*	Readmission^c^	Moderate [0.50]
Bonetti et al. [26]		Med.	MA	21		*x*	*x*	*x*	*x*	ED visit; Readmission^c^	Moderate [0.67]
Cheema et al. [27]		Med.	MA	18		*x*	*x*	*x*	*x*	ADEs; Med. discrepancy^b^; Revisit	Moderate [0.43]
Daliri et al. [28]		Med.	MA	15		*x*	*x*	*x*		ADEs; Med. adherence; Mortality; Readmission^b^	High [0.80]
Hammad et al. [29]		Med.	SR	13	*x*	*x*	*x*	*x*		LoS; Mortality; Revisit	Moderate [0.63]
Mekonnen et al. [30]		Med.	MA	17	*x*	*x*	*x*	*x*	*x*	ADEs; ED visit^b^; Revisit^b^; Mortality; Readmission^b^	Moderate [0.57]
Mekonnen et al. [31]		Med.	MA	19	*x*	*x*	*x*	*x*	*x*	Med. discrepancy^b^	Moderate [0.47]
Skjøt‐Arkil et al. [32]		Med.	SR	28	*x*	*x*	*x*	*x*	*x*	ED visit; LoS^b^; Med. error; Mortality; QoL; Revisit; Readmission^b^	High [0.75]
Villeneueve et al. [33]		Med.	SR	17	*x*	*x*	*x*	*x*	*x*	ED visit; Readmission; Revisit	Moderate [0.46]
Weeda et al. [34]		Med.	SR	11	*x*	*x*	*x*	*x*	*x*	Med. adherence^b^; Mortality; Readmission^b^	Moderate [0.63]
Backman et al. [35]		PFCC	SR	28	*x*	*x*	*x*	*x*	*x*	Health/Functional status; Patient knowledge^b^; Patient satisfaction^b^; QoL	Moderate [0.67]
Chartrand et al. [36]		PFCC	MA	50		*x*	*x*			ED visit; Readmission^c^	Moderate [0.57]
Meulenbroeks et al. [37]		PFCC	SR	23	*x*	*x*		*x*		Discharge to home^b^; ED visit; HC utilization; Health/Functional status; LoS; Mortality; Patient satisfaction^b^; QoL; Readmission	Moderate [0.54]
Rodakowski et al. [38]		PFCC	SR	15	*x*	*x*	*x*	*x*		Readmission^c^	Moderate [0.47]
Hansen et al. [39]		Int.	SR	43		*x*	*x*	*x*	*x*	Readmission	Moderate [0.50]
Hesselink et al. [40]		Int.	SR	36			*x*	*x*	*x*	ED visit; HC utilization; Med. adherence; Med. discrepancy; Mortality; Patient knowledge^b^; Patient satisfaction^b^; QoL; Readmission	Moderate [0.58]
Richards and Coast [41]		Int.	SR	23	*x*				*x*	Discharge to home; HC utilization; Health/Functional status; LoS; Mortality; Readmission	Moderate [0.54]
Gillespie et al. [42]		Edu.	MA	10		*x*			*x*	Patient satisfaction; QoL; Readmission	High [0.70]
Oh et al. [43]		Edu.	MA	7		*x*				Readmission^c^	Moderate [0.67]
Gwadry‐Sridhar et al. [44]		HFMP	MA	8		*x*			*x*	Mortality; Readmission^b^	Moderate [0.57]
Lambrinou et al. [45]		HFMP	MA	19	*x*	*x*			*x*	Readmission^b^	Moderate [0.37]
Becker et al. [46]		Comm.	MA	60		*x*				ED visit; Med. adherence^b^; Mortality; Patient knowledge; Patient satisfaction^b^; Readmission^b^	Moderate [0.47]

Abbreviations: ADEs, adverse drug events; Comm., communication; Edu., discharge education; HC utilization, post‐discharge healthcare utilization; health/functional status, patient health and/or functional status; HFMP, Heart Failure Management Program; Int., interventions; LoS, hospital length of stay; MA, systematic review including meta‐analysis; Med., medication; PFCC, patient and family centered care; QoL, quality of life; Readmission, includes 30‐day, 90‐day, 180‐day readmission, and heart‐failure (HF) readmission; Revisit, hospital revisit (readmission and/or ED visits); SR, systematic review with narrative synthesis only; TC/DP, transitional care/discharge planning.

^a^ Only considering outcomes which were included in the SoE‐analysis.

^b^ Review reported either the identification of a significant association from meta‐analysis, ≥ 2/3 of primary studies showed a significant association, or at least a moderate association was mentioned in abstract/conclusion.

^c^ Review found mixed evidence (significant and nonsignificant associations) for different readmission types.

Just under half of the reviews were restricted to specific populations or diseases, with nine considering only older adults (≥ 60 years) and six considering only patients with cardiological diagnoses.

The number of primary studies per review ranged from seven to 126 (median = 20), yielding a total of 864 primary studies across all reviews. The corrected covered area (CCA) [47] was 2%, indicating a minimal overlap of primary studies. Most primary studies were conducted in the United States (39%), with smaller contributions from a wide range of other countries (Appendix S9).

Using the AMSTAR 2 tool, we appraised the methodological quality of most reviews as moderate (*n* = 27). Five reviews achieved a high quality and two a low quality appraisal (see Appendix S10). The most common reasons for ratings below high were inadequate descriptions of study selection, missing risk‐of‐bias (RoB) assessments in meta‐analyses, and a lack of information on excluded studies.

### 3.3 Outcomes and Strength of Evidence

Table 2 presents the 19 outcomes for which we assessed the SoE for potential association with DP. More than half (*n* = 10) were related to *healthcare resource use* (readmission, ED visits, revisits, LoS, discharge to home, post‐discharge healthcare utilization). Four outcomes pertained to *medication use* (medication discrepancy, error, adherence, adverse drug events [ADEs]), and the remaining five to *patient health and status* (patient satisfaction, patient knowledge, health/functional status, quality of life [QoL], mortality).

**TABLE 2:** Overview of outcomes and strength of evidence (SoE) for their potential association with hospital DP.

Categories	Outcome^a^	Expected direction	SoE rating [score]	# of reviews which report on the outcome	# of primary studies which report on the outcome
Healthcare resource use	180‐day readmission	▼	High [0.77]	8	77
	Readmission (mixed time frames)^b^	▼	High [0.70]	19	278
	1‐year readmission	▼	High [0.67]	2	10
	90‐day readmission	▼	Moderate [0.44]	6	58
	30‐day readmission	▼	Moderate [0.38]	14	224
	Length of stay (LoS)	▼	Moderate [0.36]	6	40
	Discharge to home	▲	Moderate [0.33]	2	10
	Revisits^c^	▼	Moderate [0.33]	3	24
	ED visits	▼	Low [0.24]	12	104
	Post‐discharge healthcare utilization	▲	Low [0.22]	6	34
Patient health and status	Patient satisfaction	▲	High [0.71]	7	51
	Patient knowledge	▲	Moderate [0.40]	4	32
	Health/functional status	▲	Low [0.30]	6	53
	Quality of life (QoL)	▲	Low [0.25]	13	105
	Mortality	▼	Low [0]	13	81
Medication^d^	Medication discrepancy	▼	High [0.80]	3	21
	Medication error	▼	Moderate [0.40]	3	14
	Medication adherence	▲	Moderate [0.33]	6	34
	Adverse drug events (ADEs)	▼	Low [0]	4	14

*Note:* Expected direction: Outcome is expected to decrease (▼)/increase (▲) in response to DP intervention.

Abbreviations: ED, emergency department; SoE, strength of evidence.

^a^ For the following outcomes, we did not calculate the SoE because the outcomes were inconsistently defined or discussed in only one review: “adverse events”, “caregiver health/burden”, “caregiver quality of life”, “cost”, “cardiovascular‐related readmission”, “drug‐related readmission“, “COPD‐related readmission”, “heart failure readmission”, “medication appropriateness”, “patient confidence” (see Appendix S5).

^b^ Readmission results for which no timeframe was clearly specified were grouped under “readmission (mixed time frames)”.

^c^ Composite outcome of Readmission and ED visits.

^d^ Medication discrepancy: any difference between the medication use history and the admission medication [48]; medication error: preventable event that may (but does not have to) cause or lead to inappropriate medication use or patient harm while the medication is in the control of the healthcare professional, patient, or consumer [49].

The most frequently assessed outcome, in terms of both the number of reviews and the number of primary studies, was readmission (30‐day and mixed timeframes), followed by QoL, mortality, and ED visits. We observed no consistent relationship between the frequency with which an outcome was assessed and its corresponding SoE rating.

We rated the SoE as high for five outcomes (medication discrepancy, 180‐day readmission, patient satisfaction, readmission [mixed time frames], 1‐year readmission), moderate for eight (90‐day and 30‐day readmission, medication error, medication adherence, patient knowledge, LoS, discharge to home, revisits), and low for six (health/functional status, post‐discharge healthcare utilization, QoL, ED visits, mortality, ADEs).

We further examined whether the SoE varied by review focus or patient population (see Appendix S13). For 30‐day readmission, the overall SoE was moderate, but it was higher in the four reviews that focused specifically on medication‐related interventions. In contrast, reviews addressing a broader range of transitional care or DP interventions received a lower SoE rating for 30‐day readmission, although these yielded high SoE ratings for 180‐day and 1‐year readmission rates.

The SoE also varied by patient population. Reviews that included older patients (≥ 60 years) received an exceptionally high SoE rating for 180‐day readmission, with three out of eight reviews restricted to this population only. In contrast, only one of 14 reviews investigating 30‐day readmission was restricted to older patients. Finally, among the 19 reviews considering readmission at mixed time frames, five were restricted to cardiological patients. All five of these reviews reported evidence consistent with DP reducing readmissions.

We excluded several outcomes from the SoE analysis due to insufficient evidence. As a result, we could not assess the SoE for the association between DP and some potentially relevant but understudied outcomes (e.g., caregiver satisfaction). Although cost outcomes were frequently reported (*n* = 9), we excluded them from the SoE analysis due to substantial heterogeneity in cost definitions and calculation methods.

### 3.4 Interventions

We identified 20 different intervention types, which we grouped into five categories: *Preparation, Education, Medication, Coordination*, and *Follow‐up*. Additionally, we created a separate category, *Discharge Planning (not further specified)*, for reviews that referred to DP as an intervention but did not describe specific DP activities or elements included.

**FIGURE 3:**
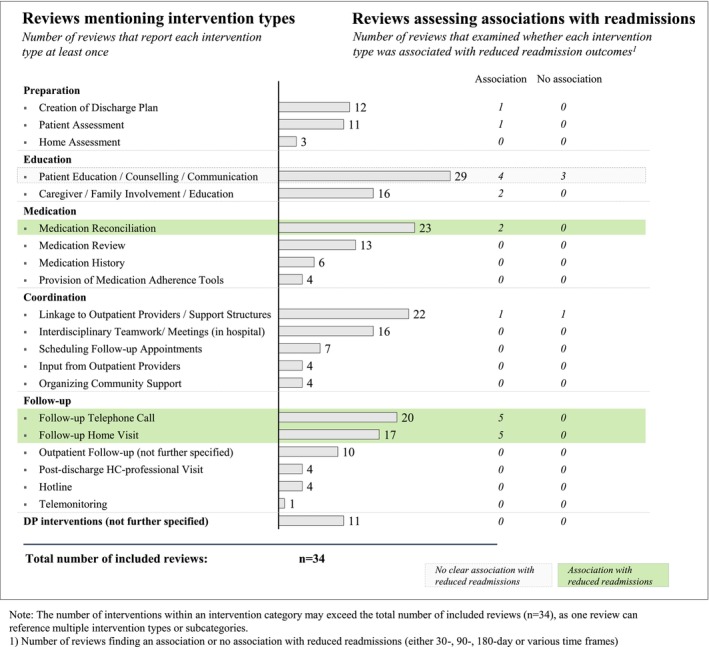
Reviews mentioning DP intervention types and assessing associations with reduced readmissions.

At the review level, the number of intervention types examined ranged from one to 16 (median = 7). Most reviews covered multiple intervention categories, with 22 reviews considering at least four of our five main categories. Only one review focused exclusively on a single intervention type (patient education) [46]. The frequency with which intervention types appeared across the reviews is illustrated in Figure 3 (further details in Appendix S11).

The *Preparation category* encompassed intervention types designed to prepare the discharge process shortly after hospital admission. Twelve reviews described interventions involving the creation of a discharge plan, eleven the assessment of patients' needs, and three the evaluation of patients' home living conditions.

In the *Education* category, intervention types involving patient education, counseling, or communication were the most frequently reported, examined in 29 reviews (85% of all reviews). These were usually delivered in person by healthcare professionals, most often nurses or pharmacists, and aimed to improve patient self‐management.

Intervention types in the *Medication* category were described in both medication‐focused and nonmedication‐focused reviews. The most common, appearing in 23 reviews (68% of all reviews), was medication reconciliation, in which a patient's prescribed medications are compared with their current medication regimen to identify and resolve medication discrepancies [50].

In the *Coordination* category, 22 reviews included intervention types aiming to establish links with outpatient providers and support structures. Examples included communication between hospital staff and primary care physicians (PCPs) through personal contact [2, 51] or the transmission of discharge letters to PCPs or community pharmacists [32, 39].

Several reviews also described post‐discharge intervention components, most commonly follow‐up telephone calls (20 reviews) or home visits by healthcare professionals (17 reviews). Lastly, 11 reviews considered general DP interventions without providing further details.

### 3.5 Subgroup Evidence

Of the 34 included reviews, 26 reported subgroup results (see Appendix S8). The number of subgroup analyses varied, ranging from one (6 reviews) to 10 (one review), while reviews varied in their methodological subgroup analysis approaches (see Appendix S14). In total, we categorized and synthesized results from 15 subgroup types (see Appendices S15 and S16).

#### 3.5.1 Findings by Intervention

Twenty‐two reviews examined the outcomes of specific intervention types, either through subgroup analysis or by focusing on a single type (e.g., education [42] or medication reconciliation [30, 31]). We identified results for eight of 20 intervention types overall, most commonly for follow‐up interventions, medication reconciliation, and patient education (see Appendix S17). The findings largely concerned readmissions, while other outcomes were rarely addressed. Figure 3 illustrates the number of reviews mentioning each intervention type and, for readmissions, whether they reported an association or no association with reduced readmissions.

Five reviews reported that follow‐up telephone calls and/or home visits were associated with reduced readmissions, and one additionally noted an association with reduced mortality [24]. We found mixed evidence for the association of medication reconciliation and healthcare outcomes. While two reviews report reductions in readmissions [23, 30] and two others identified reductions in medication discrepancies [31, 40], two reviews report no association with reduced mortality [29, 30]. Lastly, regarding patient education, four reviews reported associations with reduced readmissions, whereas three found no association.

In addition to the specific intervention types described above, reviews' subgroup analyses found further beneficial DP features such as patient‐centered approaches [39], clear discharge instructions [39], caregiver involvement [19], promotion of self‐management [22, 23], and effective hospital‐PCP communication [25].

#### 3.5.2 Findings by Intervention Intensity

Nine out of 15 reviews found that higher‐intensity, more complex, or bundled interventions were more frequently or strongly associated with positive DP outcomes than lower‐intensity, less complex, or single‐component interventions.

#### 3.5.3 Findings by Patient Characteristics

Some reviews indicated that associations between DP interventions and outcomes were stronger for older adults and for patients at higher risk. In contrast, no consistent differences were observed across subgroups defined by the number or type of providers delivering the interventions, other patient characteristics (e.g., medical conditions, gender, care settings, or geographic region), by study design, or methodological quality.

## 4 Discussion

Based on 34 systematic reviews synthesizing 864 primary studies conducted in 47 countries, we identified substantial evidence of associations between hospital DP interventions and a range of care and utilization outcomes. The low degree of overlap between primary studies across reviews, probably due to differences in review focus, underscores the need to create an overview of this broad evidence base and identify gaps in understudied areas.

A wide range of outcome measures has been examined to evaluate DP, but with a strong emphasis on healthcare resource use, particularly readmission rates. This emphasis likely reflects data availability, research path dependency, and the financial relevance of readmissions for healthcare payers and providers (e.g., HRRP in the US [8]).

Overall, we found strong evidence (high SoE) that DP interventions are associated with reduced hospital readmissions. Consistent with earlier umbrella reviews [3, 6, 7] and the multicomponent nature of DP, our analysis suggests that several mechanisms may jointly account for these reductions. Based on our analysis, potential mechanisms include improved patient knowledge and self‐care [46], optimized medication management [28], early identification of post‐discharge complications, and timely scheduling of outpatient follow‐up care [52].

When comparing follow‐up periods, we found higher SoE for medium‐term (180‐day) and long‐term (1‐year) readmissions than for short‐term (30 and 90‐day) readmissions. Several factors may contribute to this pattern. Short‐term readmissions are by definition less frequent and more likely to be driven by factors outside the immediate scope of DP (e.g., quality of medical care, clinical severity) [53, 54, 55], making it more difficult to isolate the specific effect of DP interventions. Moreover, certain components of DP, especially those targeting longer‐term behavioral changes (e.g., patient education), may require an extended period before measurable effects appear [56, 57]. In contrast, our SoE subgroup analysis suggests that medication‐related interventions may be particularly effective in reducing 30‐day readmissions, indicating that prompt access to medications and patient education on their use may yield more immediate improvements in this quality indicator.

The higher SoE for medium‐ and long‐term readmissions may also reflect greater effectiveness of DP among older patients, who often require more extensive discharge support due to higher levels of multimorbidity or frailty [58, 59, 60]. Because the reviews examining 30‐day readmissions included older populations less frequently than those examining 180‐day readmissions, our results may be systematically biased towards younger patient groups. To better understand the temporal relationship between DP and readmissions, future research should directly compare short‐, medium‐, and long‐term outcomes within consistent patient populations. In addition, studies should investigate the mechanisms through which DP influences readmissions (e.g., via mediation analysis) to help explain variations across follow‐up periods.

We found moderate to low SoE for associations between DP and other healthcare utilization outcomes. The moderate SoE for LoS may reflect competing mechanisms [5]. On the one hand, DP may improve hospital process efficiency, thereby contributing to a reduction in LoS. On the other hand, DP may increase LoS as additional time is needed to ensure patient discharge readiness and the coordination of appropriate follow‐up care. Although the LoS‐reducing mechanism appears to predominate, opposing effects may help explain the overall moderate SoE. It is also important to note that we excluded reviews that focused on early discharge or hospital‐at‐home services [61], as these primarily aim to reduce LoS rather than promote continuity of care. Including such reviews would likely have yielded stronger evidence of an LoS‐reducing effect. Future research should explore the potential dual impact of DP on LoS and examine how this relationship varies with patient characteristics and health system contexts.

For medication‐related outcomes, we found high SoE that DP interventions may reduce medication discrepancies, moderate SoE for an association with reduced medication errors and improved medication adherence, and low SoE for an association with reduced adverse drug events (ADEs). This pattern suggests that DP interventions may be more effective for outcomes within the direct control of healthcare providers (e.g., identifying and resolving medication discrepancies) than for outcomes that rely on patient behavior after discharge (e.g., medication adherence). In addition, medication adherence is harder to measure, often relying on self‐reported data, heterogeneous scales, and longer follow‐up periods [62, 63], whereas medication discrepancies are directly observable at discharge. The low SoE for ADEs and moderate SoE for medication errors may also reflect inconsistent outcome definitions, with both terms typically describing a broad and heterogeneous set of endpoints [64, 65].

For patient health outcomes, the evidence was mixed. The highest SoE was found for a potential association between DP and improved patient satisfaction. According to classic frameworks on patient‐centered care, components such as clear communication, personalized care, and active patient involvement may help patients feel informed and respected, thereby contributing to higher satisfaction [66, 67]. In contrast, we found little to no evidence of an association with mortality or QoL. Mortality is influenced by multiple complex factors that DP interventions alone cannot fully address, including disease severity and comorbidities [68, 69]. Similarly, QoL depends on factors beyond the scope of DP and is often measured using diverse definitions and tools (see Appendix S5), introducing variability and imprecision into reported findings.

In addition to assessing the SoE for potential associations between DP and 19 different outcomes, we synthesized 20 intervention types into five categories spanning the patient journey from hospital admission to post‐discharge care. Both the intervention categories and the individual intervention types were consistent with established transitional care models (e.g., the Transitional Care Model (TCM) [70] or the Ideal Transition in Care process [13]). Medication‐related intervention types appeared both in medication‐focused and nonmedication‐focused reviews, reinforcing the importance of medication management in DP. In contrast, intervention types designed to initiate and prepare the discharge process early in the hospital stay were the least frequently reported, suggesting an area for future research. Lastly, the category DP (not further specified) illustrates the definitional ambiguity of the term itself. While some reviews conceptualized DP as a collection of distinct interventions, others framed it as a subcomponent of broader constructs such as transitional care [16] or patient handover [40]. This diversity of conceptual approaches and definitional heterogeneity has also been noted in previous umbrella reviews [3, 7].

Our synthesis of subgroup analyses supports findings from earlier umbrella reviews. In line with Kansagara et al. [7], we found that a combination of different intervention types (bundled approaches), including follow‐up components, tends to be associated with improved outcomes, particularly reduced readmissions. Consistent with Straßner et al. [6], we found some evidence that high‐intensity DP interventions targeting older adults and at‐risk patients may be particularly effective, especially in reducing readmissions. However, because the evidence base still consists mainly of studies on readmissions, future research should examine intervention‐ and population‐specific effects on a broader range of outcomes.

Our study has several limitations. First, we may have missed relevant literature due to the wide variation in terminology used to describe DP. Although most studies use the terms “transitional care” or “discharge planning”, others use alternative definitions. To address this issue, we employed a broad search strategy and conducted additional manual searches. Second, our synthesis is limited by heterogeneity in study designs and the way in which interventions were described, both in the included reviews and their underlying primary studies, a challenge noted previously in the literature [32, 39]. To improve future evidence syntheses, researchers and practitioners should establish and use common definitions, core outcome sets, and standardized intervention protocols. This is particularly pressing for cost‐related outcomes, as the current evidence base provides little insight into the cost‐effectiveness of DP, which is a highly relevant aspect for healthcare decision‐making. Lastly, our SoE method required setting thresholds, which inherently influences the ratings. To address this, we based our approach on previous research [12] and conducted sensitivity analyses, which confirmed the stability of our results. Nonetheless, when only a small number of reviews are available for a given outcome, the influence of individual review findings on SoE ratings is greater. As the evidence base grows, future research may permit more robust SoE calculations for outcomes that are currently underrepresented.

Despite these limitations, our umbrella review makes several valuable contributions. First, we provide an extensive overview and categorization of hospital DP intervention types to support future researchers in selecting and classifying interventions for study.

Second, we build upon previous umbrella reviews, which concluded that the effectiveness of DP varies across outcomes [3, 5], by systematically assessing the SoE for 19 different outcomes, taking into account the methodological quality of the included reviews. In doing so, we provide a comprehensive overview of outcome coverage, identifying well‐studied outcomes (e.g., readmission), as well as those that remain underexplored, inconsistently defined, or measured (e.g., costs).

Third, we substantially expand on previous tentative findings regarding effect heterogeneity by applying two complementary approaches: (1) systematically extracting and synthesizing the results of subgroup analyses reported in existing reviews and (2) assessing whether the SoE for the potential associations between DP and each outcome varied across review focus or patient population. Although subgroup definitions and analytical methods varied widely, our analysis nonetheless suggests that DP interventions may be beneficial for certain patient groups and intervention types. To verify these findings, future research should aim to standardize subgroup definitions and systematically compare effect sizes across subgroups.

## 5 Conclusion

Knowing when and under which circumstances hospital DP is most effective is a central concern for research, policy, and practice. In this umbrella review, we synthesized evidence from 34 systematic reviews covering various types of hospital DP interventions and assessed the strength of evidence (SoE) for their associations with 19 outcomes. The strongest evidence was found for associations with reduced hospital readmissions, fewer medication discrepancies, and greater patient satisfaction. Evidence for associations with QoL, mortality, ED visits, and overall patient health status was weak or lacking. Policymakers and clinicians should therefore consider prioritizing DP when the main objective is to reduce rehospitalizations (e.g., HRRP) and medication‐related problems. Regarding the design of DP, high‐intensity, bundled programs with follow‐up and medication‐related interventions appeared to be most effective, particularly for reducing readmissions. Together, these findings provide direction for evaluating DP programs and tailoring them to meet specific healthcare objectives. Future research should prioritize the development and use of standardized definitions, core outcome sets, and consistent subgroup classifications. In addition, statistical comparisons of effect sizes and mediation analyses are needed to clarify the mechanisms through which DP interventions may influence quality of care and patient outcomes.

## Conflicts of Interest

The authors declare no conflicts of interest.

## Supporting information


**Data S1:** Supporting Information.

## Data Availability

Data sharing not applicable to this article as no datasets were generated or analysed during the current study.
