# Supplementary material for: Hospital Discharge Planning—An Investigation of Outcomes and Interventions
Source: Health Serv Res. 2025 Oct 23;61(1):e70060. doi: 10.1111/1475-6773.70060 (PMC12857470; doi:10.1111/1475-6773.70060)
Supplement: Supplementary file 1 — Data S1: Supporting Information. [file HESR-61-0-s001.docx]

**APPENDIX**

# **S1: PRIOR Checklist**

| **Section**  Topic | **#** | **Item** | **Location reported** |
| --- | --- | --- | --- |
| TITLE | | |  |
| Title | 1 | Identify the report as an overview of reviews. | Title |
| ABSTRACT | | |  |
| Abstract | 2 | Provide a comprehensive and accurate summary of the purpose, methods, and results of the overview of reviews. | Abstract |
| INTRODUCTION | | |  |
| Rationale | 3 | Describe the rationale for conducting the overview of reviews in the context of existing knowledge. | Introduction |
| Objectives | 4 | Provide an explicit statement of the objective(s) or question(s) addressed by the overview of reviews. | Introduction |
| METHODS | | |  |
| Eligibility criteria | 5a | Specify the inclusion and exclusion criteria for the overview of reviews. If supplemental primary studies were included, this should be stated, with a rationale. | Selection criteria and screening process/ Appendix (S3) |
|  | 5b | Specify the definition of ‘systematic review’ as used in the inclusion criteria for the overview of reviews. | Selection criteria and screening process |
| Information source | 6 | Specify all databases, registers, websites, organizations, reference lists, and other sources searched or consulted to identify systematic reviews and supplemental primary studies (if included). Specify the date when each source was last searched or consulted. | Search Strategy |
| Search strategy | 7 | Present the full search strategies for all databases, registers and websites, such that they could be reproduced. Describe any search filters and limits applied. | Search Strategy/  Appendix (S2) |
| Selection process | 8a | Describe the methods used to decide whether a systematic review or supplemental primary study (if included) met the inclusion criteria of the overview of reviews. | Selection criteria and screening process |
|  | 8b | Describe how overlap in the populations, interventions, comparators, and/or outcomes of systematic reviews was identified and managed during study selection. | Data extraction and analysis |
| Data collection process | 9a | Describe the methods used to collect data from reports. | Data extraction and analysis |
|  | 9b | If applicable, describe the methods used to identify and manage primary study overlap at the level of the comparison and outcome during data collection. For each outcome, specify the method used to illustrate and/or quantify the degree of primary study overlap across systematic reviews. | Description of reviews |
|  | 9c | If applicable, specify the methods used to manage discrepant data across systematic reviews during data collection. | n.a. |
| Data items | 10 | List and define all variables and outcomes for which data were sought. Describe any assumptions made and/or measures taken to identify and clarify missing or unclear information. | Data extraction and analysis/ Appendix (S8) |
| Risk of bias assessment | 11a | Describe the methods used to assess risk of bias or methodological quality of the included systematic reviews. | Assessment of methodological quality |
|  | 11b | Describe the methods used to collect data on (from the systematic reviews) and/or assess the risk of bias of the primary studies included in the systematic reviews. Provide a justification for instances where flawed, incomplete, or missing assessments are identified but not re-assessed. | n.a. |
|  | 11c | Describe the methods used to assess the risk of bias of supplemental primary studies (if included). | n.a. |
| Synthesis methods | 12a | Describe the methods used to summarize or synthesize results and provide a rationale for the choice(s). | Data extraction and analysis/ Appendix (S6) |
|  | 12b | Describe any methods used to explore possible causes of heterogeneity among results. | Appendix (S14) |
|  | 12c | Describe any sensitivity analyses conducted to assess the robustness of the synthesized results. | Data extraction and analysis/ Appendix (S12) |
| Reporting bias assessment | 13 | Describe the methods used to collect data on (from the systematic reviews) and/or assess the risk of bias due to missing results in a summary or synthesis (arising from reporting biases at the levels of the systematic reviews, primary studies, and supplemental primary studies, if included). | Assessment of methodological quality |
| Certainty assessment | 14 | Describe the methods used to collect data on (from the systematic reviews) and/or assess certainty (or confidence) in the body of evidence for an outcome. | Data extraction and analysis/ Appendix (S6) |

| RESULTS | | |  |
| --- | --- | --- | --- |
| Systematic review and supplemental primary study selection | 15a | Describe the results of the search and selection process, including the number of records screened, assessed for eligibility, and included in the overview of reviews, ideally with a flow diagram. | Screening results an inclusion of reviews |
|  | 15b | Provide a list of studies that might appear to meet the inclusion criteria, but were excluded, with the main reason for exclusion. | Appendix (S7) |
| Characteristics of systematic reviews and supplemental primary studies | 16 | Cite each included systematic review and supplemental primary study (if included) and present its characteristics. | Description of reviews/ Appendix (S8) |
| Primary study overlap | 17 | Describe the extent of primary study overlap across the included systematic reviews. | Description of reviews/ Appendix (S9) |
| Risk of bias in systematic reviews, primary studies, and supplemental primary studies | 18a | Present assessments of risk of bias or methodological quality for each included systematic review. | Description of reviews/ Appendix (S10) |
|  | 18b | Present assessments (collected from systematic reviews or assessed anew) of the risk of bias of the primary studies included in the systematic reviews. | n.a. |
|  | 18c | Present assessments of the risk of bias of supplemental primary studies (if included). | n.a. |
| Summary or synthesis of results | 19a | For all outcomes, summarize the evidence from the systematic reviews and supplemental primary studies (if included). If meta-analyses were done, present for each the summary estimate and its precision and measures of statistical heterogeneity. If comparing groups, describe the direction of the effect. | Outcomes and Strength of Evidence/  Synthesis of subgroup analyses in the included reviews/ Appendix (S12, S13, S14, S15, S16) |
|  | 19b | If meta-analyses were done, present results of all investigations of possible causes of heterogeneity. | n.a. |
|  | 19c | If meta-analyses were done, present results of all sensitivity analyses conducted to assess the robustness of synthesized results. | n.a. |
| Reporting biases | 20 | Present assessments (collected from systematic reviews and/or assessed anew) of the risk of bias due to missing primary studies, analyses, or results in a summary or synthesis (arising from reporting biases at the levels of the systematic reviews, primary studies, and supplemental primary studies, if included) for each summary or synthesis assessed. | Data extraction and analysis/  Appendix (S10) |
| Certainty of evidence | 21 | Present assessments (collected or assessed anew) of certainty (or confidence) in the body of evidence for each outcome. | Outcomes and Strength of Evidence/Appendix (S12, S13) |
| DISCUSSION | | |  |
| Discussion | 22a | Summarize the main findings, including any discrepancies in findings across the included systematic reviews and supplemental primary studies (if included). | Discussion |
|  | 22b | Provide a general interpretation of the results in the context of other evidence. | Discussion |
|  | 22c | Discuss any limitations of the evidence from systematic reviews, their primary studies, and supplemental primary studies (if included) included in the overview of reviews. Discuss any limitations of the overview of reviews methods used. | Discussion |
|  | 22d | Discuss implications for practice, policy, and future research (both systematic reviews and primary research). Consider the relevance of the findings to the end users of the overview of reviews, e.g., healthcare providers, policymakers, patients, among others. | Discussion/  Conclusion |
| OTHER INFORMATION | | |  |
| Registration and protocol | 23a | Provide registration information for the overview of reviews, including register name and registration number, or state that the overview of reviews was not registered. | PROSPERO-registration |
|  | 23b | Indicate where the overview of reviews protocol can be accessed, or state that a protocol was not prepared. | Methods |
|  | 23c | Describe and explain any amendments to information provided at registration or in the protocol. Indicate the stage of the overview of reviews at which amendments were made. | Methods |
| Support | 24 | Describe sources of financial or non-financial support for the overview of reviews, and the role of the funders or sponsors in the overview of reviews. | Title page |
| Competing interests | 25 | Declare any competing interests of the overview of reviews’ authors. | Title page |
| Author information | 26a | Provide contact information for the corresponding author. | Title page |
|  | 26b | Describe the contributions of individual authors and identify the guarantor of the overview of reviews. | / |
| Availability of data and other materials | 27 | Report which of the following are available, where they can be found, and under which conditions they may be accessed: template data collection forms; data collected from included systematic reviews and supplemental primary studies; analytic code; any other materials used in the overview of reviews. | Data extraction and analysis/ Appendix  (S2, S8) |

# **S2: Search Strategy**

| **PubMed (20.02.2024):**  ("discharge plan*"[Title/Abstract] OR "discharge management"[Title/Abstract] OR "transitional intervention"[Title/Abstract] OR "transitional care" [Title/Abstract] OR "transition of care" [Title/Abstract] OR "continuity of care"[Title/Abstract] OR "coordination of care"[Title/Abstract] OR "care coordination"[Title/Abstract] OR "discharge intervention*"[Title/Abstract] OR "discharge process*"[Title/Abstract] OR "discharge arrangement"[Title/Abstract] OR "discharge educat*"[Title/Abstract] OR "discharge training"[Title/Abstract] OR ("medication reconciliat*"[Title/Abstract] AND discharg*[Title/Abstract]) OR "Patient Discharge"[MeSH])  AND (hospital*[Title/Abstract] OR "acute care"[Title/Abstract] OR inpatient[Title/Abstract] OR "in-patient"[Title/Abstract] OR hospitals[MeSH])  AND ("effect*"[Title/Abstract] OR "outcome*"[Title/Abstract] OR "impact*"[Title/Abstract] OR "result*"[Title/Abstract] OR "influenc*"[Title/Abstract] OR "quality"[Title/Abstract] OR "length of stay"[Title/Abstract] OR "readmission*"[Title/Abstract] OR "patient satisfaction"[Title/Abstract] OR "patient safety"[Title/Abstract] OR "rehospitali*"[Title/Abstract] OR mortality [Title/Abstract] OR "Quality of Health Care"[MeSH])  NOT (placebo[Title/Abstract]) NOT (psych*[Title]) NOT (child*[Title]) NOT (infant*[Title]) |
| --- |

For other databases, the search prompt was adapted accordingly.

# **S3: Detailed inclusion / exclusion criteria (PICOS)**

|  | **Inclusion criteria** | **Exclusion criteria** |
| --- | --- | --- |
| **Participants** | Adults (>17 years) | - Children - Psychiatric patients - Palliative patients |
| **Intervention** | - Interventions must be initiated during the hospital stay and focus on pre-discharge and bridging interventions - Eligible interventions must explicitly aim to smooth the transition from hospital to home or another care facility and/or to prevent adverse events after discharge - Interventions may be standalone or embedded as components within broader discharge support structures (e.g., case management), and may be delivered in conjunction with other interventions or care programs (e.g., case management). | - Focus on post-discharge interventions - No pre-discharge/bridging elements - Interventions that constitute only a minor or indistinct part of a broader continuous care program - Discharge occurring from the emergency department, or within-hospital transfers (e.g., between wards) - Interventions explicitly aiming to reduce length of hospital stay (i.e., early discharge programs) |
| **Comparison** | Usual or standard care | - |
| **Outcome** | - Outcomes must be used to measure the effectiveness of discharge planning interventions (e.g., hospital length of stay, readmission rates, patient or caregiver satisfaction) - Studies must report a quantitative outcome with an associated level of statistical significance (e.g., p-value or confidence interval) | - Studies that lack comprehensiveness (e.g., those focusing narrowly on a specific patient group or disease) - Outcomes that are not generalizable beyond the specific population or disease under study |
| **Study design** | - Peer-reviewed systematic literature reviews and/or meta-analyses - At least two-thirds of the included primary studies must meet the predefined eligibility criteria for population, intervention and outcomes | - Primary studies (e.g., RCTs, observational studies), qualitative studies, umbrella reviews - Systematic literature reviews with fewer than five primary studies |

# **S4: Adaptation of AMSTAR2 checklist**

| **No.** | **Item** | **Original item modified?** | **Item description** | **No weighting** | **Weighting** | **Comment** |
| --- | --- | --- | --- | --- | --- | --- |
| **1** | **PICO** | No | *Did the research questions and inclusion criteria for the review include the components of PICO?* | 1 | 2 |  |
| **2** | **Methods/Protocol** | Yes | *Did the authors state that the review methods were established prior to the conduct of the review OR that the methods were based on a standardized written protocol/guide, and did the report justify any significant deviations from the determined methods/protocol?* | 1 | 2 | *Item description adapted as in Blume et al. (2021)* |
| **3** | **Selection Study Designs** | No | *Did the review authors explain their selection of the study designs for inclusion in the review?* | 1 | 1 |  |
| **4** | **Search Strategy** | Yes | *Did the review authors use a comprehensive literature search strategy?* | 1 | 3 | *Included "years searched" as additional criterion for partial yes; we did not consider language restrictions* |
| **5** | **Duplicate study selection** | No | *Did the review authors perform study selection in duplicate?* | 1 | 2 |  |
| **6** | **Duplicate Data Extraction** | No | *Did the review authors perform data extraction in duplicate?* | 1 | 2 |  |
| **7** | **Excluded Studies** | No | *Did the review authors provide a list of excluded studies and justify the exclusions?* | 1 | 3 |  |
| **8** | **Description Included Studies** | No | *Did the review authors describe the included studies in adequate detail?* | 1 | 3 |  |
| **9** | **Risk of Bias (RoB)** | No | *Did the review authors use a satisfactory technique for assessing the risk of bias (RoB) in individual studies that were included in the review?* | 1 | 3 |  |
| ***10*** | ***Sources of Funding*** | Yes | *Did the review authors report on the sources of funding for the studies included in the review?* | 1 | - | *Item description similar to Blume et al. (2021): Item 10 left out due to limited relevance in our research context* |
| **11** | **Justification Meta-Analysis** | No | *If meta-analysis was performed, did the review authors use appropriate methods for statistical combination of results?* | 1 | 3 |  |
| **12** | **RoB in Meta-Analysis** | No | *If meta-analysis was performed, did the review authors assess the potential impact of RoB in individual studies on the results of the meta-analysis or other evidence synthesis?* | 1 | 3 |  |
| **13** | **RoB individual studies** | No | *Did the review authors account for RoB in individual studies when interpreting/discussing the results of the review?* | 1 | 3 |  |
| **14** | **Heterogeneity** | No | *Did the review authors provide a satisfactory explanation for, and discussion of, any heterogeneity observed in the results of the review?* | 1 | 3 | *Weighting: Due to the heterogeneity in study designs, interventions, and outcome measurements, this aspect was considered particularly relevant to our research question and was therefore assigned a higher weight* |
| **15** | **Publication Bias** | No | *If they performed quantitative synthesis, did the review authors carry out an adequate investigation of publication bias (small study bias) and discuss its likely impact on the results of the review?* | 1 | 3 |  |
| **16** | **Conflict of interest** | No | *Did the review authors report any potential sources of conflict of interest, including any funding they received for conducting the review?* | 1 | 1 |  |

# **S5: Synthesis of reported outcomes**

| **Outcome** | **Included in SoE?** | **Review** | **Original description of outcome in the respective review** |
| --- | --- | --- | --- |
| **Adverse events (AE)** | *No* | Hesselink et al. (2012) | (Preventable) adverse outcomes/events |
|  |  | Rennke et al. (2013) | Other adverse events (e.g., falls, infection rates, post-discharge adverse events, post-discharge infection rates) |
| **Adverse drug events (ADEs)** | *Yes* | Cheema et al. (2018) | Potential and preventable adverse drug events |
|  |  | Daliri et al. (2021) | MRPs (Medication-Related Problems) within 30 days of discharge: definition by studies: overall MRPs, adverse drug events, or a combination of adverse drug events and medication errors |
|  |  | Mekonnen et al. (a) (2016) | Patients with ADE |
|  |  | Rennke et al. (2013) | Medication-related injury, probable adverse drug event, adverse drug reaction (ADR), preventable ADEs |
| **Caregiver health/burden** | *No* | Meulenbroeks et al. (2021) | Caregiver objective burden of caregiving, self-rated burden of caregiving, depression, prostration, mental health |
| **Caregiver QoL** | *No* | Meulenbroeks et al. (2021) | Caregiver: QoL, QUALY |
| **COPD-readmission** | *No* | Park et al. (2023) | Readmission for COPD (number of readmissions) |
| **Cost** | *No* | Albert et al. (2016) | Hospital cost, cost savings per patient & intervention Group (open time frame / over 8 months), mean-non elective cost (30, 60, 180-day post intervention), observed cost, net savings, health care service cost, medical cost, mean cost for interventions |
|  |  | Allen et al. | Health care costs (inclusive of inpatient, clinic, home visits), total mean costs |
|  |  | Gonçalves-Bradley et al. (2022) | Healthcare resource use and costs (readmission costs, ED-visit costs, intervention cost, caregiver cost, total costs) |
|  |  | Hammad et al. (2017) | Cost related to extra time of intervention (MR), cost savings related to hospital and emergency department revisits, health resource use in community the time of doctors and nurses freed from obtaining |
|  |  | Lee et al. (2022) | Medical costs, cost-effectiveness of intervention, cost-effective considering quality-adjusted life years |
|  |  | Meulenbroeks et al. (2021) | Acute resources cost, cost for unplanned readmission, hospitalization cost |
|  |  | Rodakowski et al. (2017) | Costs of post-discharge care, cost of initial hospitalization |
|  |  | Skjøt-Arkil et al. (2018) | Cost effectiveness: reduction in cost of hospital care by calculating the saved LOS of readmissions against the cost of pharmacy staff |
|  |  | Stamp et al. (2014) | Direct vs. indirect costs |
| **Cardiovascular (CV)-related readmission** | *No* | Weeda et al. (2023) | CV-related encounters (any CV-related hospital readmission, readmission with PCI, readmission due to in-stent thrombosis/ restenosis/ MI, revascularization) |
| **Discharge to home** | *Yes* | Gonçalves-Bradley et al. (2022) | Place of discharge |
|  |  | Lee et al. (2022) | Effect on nursing home placement, days spent in nursing homes, institutionalization rate, days at home |
|  |  | Meulenbroeks et al. (2021) | Patient: discharge destination |
|  |  | Richards and Coast (2003) | Use of services post-discharge for old/frail people: nursing home admission vs. discharged to home (discharge destination) |
| **Drug-related readmission** | *No* | Villeneueve et al. (2021) | Drug-related readmission/hospitalizations |

| **Outcome** | **Included in SoE?** | **Review** | **Outcome description in review** |
| --- | --- | --- | --- |
| **Drug-related revisits (readmission and ED-visits)** | *Yes* | Cheema et al. (2018) | Healthcare utilization as drug related ED-visits or readmissions |
|  |  | Mekonnen et al. (a) (2016) | Adverse drug event-related hospital revisits |
| **ED-visits** | *Yes* | Albert et al. (2016) | ED-visits |
|  |  | Becker et al. (2021) | ED reattendance 30, 180 days |
|  |  | Bonetti et al. (2021) | ED visit rate |
|  |  | Chartrand et al. (2023) | ED visits |
|  |  | Hesselink et al. (2012) | ED visits |
|  |  | Leithaus et al. (2022) | ED visits |
|  |  | Mekonnen et al. (a) (2016) | All-cause ED visits |
|  |  | Meulenbroeks et al. (2021) | ED use |
|  |  | Park et al. (2023) | Emergency department visits for COPD |
|  |  | Rennke et al. (2013) | ED visits |
|  |  | Skjøt-Arkil et al. (2018) | ED visits |
|  |  | Villeneueve et al. (2021) | ED visits |
| **Health/ functional status** | *Yes* | Albert et al. (2016) | Self-rated health / perception of health |
|  |  | Backman et al. (2020) | Functional status, patient stress (30days), depressive symptoms |
|  |  | Lee et al. (2022) | Physical function, self-rated health, mental health and life satisfaction |
|  |  | Meulenbroeks et al. (2021) | Physical health, mental health, function, performance, psychological wellbeing |
|  |  | Park et al. (2023) | Physical capacity (six-minute walk test, number of steps per day) |
|  |  | Richards and Coast (2003) | Functional health status and disability, patient perception of health, cognitive functioning and psychological well-being (perceived well-being) |
| **Heart failure (HF) readmission** | *No* | Lambrinou et al. (2012) | HF readmissions (heart failure-related hospitalizations) |
| **Length of stay (LOS)** | *Yes* | Gonçalves-Bradley et al. (2022) | Hospital length of stay |
|  |  | Hammad et al. (2017) | Average hospital stay |
|  |  | Hesselink et al. (2012) | Length of hospital stay |
|  |  | Meulenbroeks et al. (2021) | Patient: LOS |
|  |  | Richards and Coast (2003) | Use of services post-discharge for old/frail people: LOS |
|  |  | Skjøt-Arkil et al. (2018) | LOS of index admission |

| **Outcome** | **Included in SoE?** | **Review** | **Outcome description in review** |
| --- | --- | --- | --- |
| **Medication adherence** | *Yes* | Becker et al. (2021) | Medication adherence 7, 10, 30 days |
|  |  | Daliri et al. (2021) | Medication adherence 1, 3, 6, 12 months |
|  |  | Hesselink et al. (2012) | Medication adherence |
|  |  | Gonçalves-Bradley et al. (2022) | Medication adherence |
|  |  | Weeda et al. (2023) | Medication adherence |
| **Medication appropriateness** | *No* | Skjøt-Arkil et al. (2018) | Medication appropriateness |
| **Medical discrepancy** | *Yes* | Cheema et al. (2018) | Medication discrepancies (incl. characteristics and clinical severity of such medication discrepancies) |
|  |  | Hesselink et al. (2012) | Medication discrepancies (between hospital and community pharmacy records) |
|  |  | Mekonnen et al. (b) (2016) | Proportion of patients with medication discrepancies |
| **Medicational error** | *Yes* | Gonçalves-Bradley et al. (2022) | Medication error (problems with medication after discharge from hospital) |
|  |  | Skjøt-Arkil et al. (2018) | Medication error |
|  |  | Tomlinson et al. (2020) | Error (dose frequency / dose / name) |
| **Mortality** | *Yes* | Becker et al. (2021) | Death at 10, 30 days, overall mortality at 180 days |
|  |  | Daliri et al. (2021) | Mortality 1, 3, 6, 12 months |
|  |  | Gonçalves-Bradley et al. (2022) | Mortality at 3 to 9 months |
|  |  | Gwadry-Sridhar et al. (2004) | Relative risks of mortality |
|  |  | Hammad et al. (2017) | Mortality (12 months) |
|  |  | Hesselink et al. (2012) | Death |
|  |  | Lee et al. (2022) | Mortality |
|  |  | Mekonnen et al. (a) (2016) | All-cause mortality |
|  |  | Meulenbroeks et al. (2021) | Patient mortality |
|  |  | Richards and Coast (2003) | Mortality |
|  |  | Skjøt-Arkil et al. (2018) | Mortality in a follow-up period of 3–12 month |
|  |  | Tomlinson et al. (2020) | Mortality (3, 6 months) |
|  |  | Weeda et al. (2023) | All-cause mortality |
| **Patient confidence** | *No* | Gillespie et al. (2023) | Patient confidence |
| **Patient satisfaction** | *Yes* | Allen et al. (2014) | Satisfaction with care, discharge and transition care, discharge arrangements and preparation, documentation, discharge planning |
|  |  | Backman et al. (2020) | Overall patient satisfaction, satisfaction with healthcare education, quality of the transitional care experience |

| **Outcome** | **Included in SoE?** | **Review** | **Outcome description in review** |
| --- | --- | --- | --- |
| **Patient satisfaction** *(continued)* | *Yes* | Becker et al. (2021) | General satisfaction (30 days), satisfaction with explanation (30 days) |
|  |  | Gillespie et al. (2023) | Patient satisfaction |
|  |  | Gonçalves-Bradley et al. (2022) | Satisfaction of patients, caregivers and healthcare staff |
|  |  | Hesselink et al. (2012) | Satisfaction |
|  |  | Mabire et al. (2018) | Satisfaction with patient care |
| **Patient knowledge** | *Yes* | Albert et al. (2016) | Patients' knowledge, self-management scores for health, and understanding warnings and purpose of medication, ability to identify discharge diagnoses and to name their PCP |
|  |  | Backman et al. | Heart failure knowledge |
|  |  | Becker et al. (2021) | Patient knowledge |
|  |  | Hesselink et al. (2012) | Self-perceived medication understanding |
| **Post-discharge healthcare utilization** | *Yes* | Albert et al. (2016) | PCP follow-up, likelihood of completing follow-up visits, patient communication with physician |
|  |  | Allen et al. (2014) | Access to health services (likelihood of referral to community-based services / to be allocated home help) |
|  |  | Hesselink et al. (2012) | Follow-up visits by GP, more actions initiated by GP on receipt of information |
|  |  | Meulenbroeks et al. (2021) | Outpatient resource use: Generally, resource use (e.g., doctors’ appointment, home visits etc.), caregiver resource use |
|  |  | Richards and Coast (2003) | Use of services post-discharge for old/frail people: GP visit, community health services |
|  |  | Villeneueve et al. (2021) | GP visits, other health care services (home visits by GP, planned ambulatory contacts, ambulatory care visits, physicians outside working hours, medical specialist contact, hospital outpatient clinics) |
| **Quality of life (QoL)** | *Yes* | Albert et al. (2016) | HF-specific QoL, physical QoL, HR-QoL |
|  |  | Allen et al. (2014) | QoL, mental quality of life |
|  |  | Backman et al. (2020) | Quality of life (4weeks, 12weeks, and 24weeks) |
|  |  | Gillespie et al. (2023) | Patient quality of life (QoL) (overall or with dimensions: physical function, social function, emotional well-being, energy/fatigue, role limitation emotional, general health perception, role limitations physical, bodily pain) |
|  |  | Hammad et al. (2017) | Quality of life |
|  |  | Hesselink et al. (2012) | Quality of life |
|  |  | Lee et al. (2022) | Health-related QoL (using the EQ-5D, medical outcomes study survey (12-item/36-item), Quality of Well-Being Scale) |
|  |  | Mabire et al. (2018) | QoL |
|  |  | Meulenbroeks et al. (2021) | Quality of life (QoL) (CSAL, EQ-5D, SF-12, SF-36) |
|  |  | Park et al. (2023) | Respiratory-related Quality of Life (St. George’s respiratory questionnaire) |
|  |  | Skjøt-Arkil et al. (2018) | HRQL (by use of the questionnaires EQ-5D and SF-36) |
|  |  | Stamp et al. (2014) | Quality of life (Minnesota living with heart failure questionnaire, chronic heart failure questionnaire9 |
|  |  | Tomlinson et al. (2020) | QOL |
| **Outcome** | **Included in SoE?** | **Review** | **Outcome description in review** |
| **Readmission (mixed time frames)** | *Yes* | Albert et al. (2016) | Readmission |
|  |  | Bonetti et al. (2021) | Hospital readmission rates |
|  |  | Chartrand et al. (2023) | Hospital readmissions |
|  |  | Fønss Rasmussen et al. (2021) | Readmission |
|  |  | Gonçalves-Bradley et al. (2022) | Unscheduled readmission to hospital |
|  |  | Gwadry-Sridhar et al. (2004) | Relative risks of readmissions to the hospital |
|  |  | Oh et al. (2023) | Overall readmission rates |
|  |  | Hesselink et al. (2012) | Unplanned rehospitalizations, hospitalizations |
|  |  | Lambrinou et al. (2012) | All-cause readmissions (hospitalizations due to any cause) |
|  |  | Leithaus et al. (2022) | Hospital readmissions at different post-discharge measurement moments |
|  |  | Mabire et al. (2018) | Readmission |
|  |  | Mekonnen et al. (a) (2016) | All-cause readmissions |
|  |  | Meulenbroeks et al. (2021) | Unplanned readmission |
|  |  | Richards and Coast (2003) | Use of services post-discharge for old/frail people: readmission |
|  |  | Rodakowski et al. (2017) | Readmissions for any cause |
|  |  | Skjøt-Arkil et al. (2018) | Readmissions |
|  |  | Stamp et al. (2014) | Readmission (from 30days - 18months post-discharge) |
|  |  | Tomlinson et al. (2020) | All-cause hospital readmission |
|  |  | Villeneueve et al. (2021) | Hospital readmission |
| **Readmission (30-day)** | *Yes* | Verhaegh et al. (2014) | Readmission at 30 days |
|  |  | Lee et al. (2022) | Readmission 1 Month |
|  |  | Rennke et al. (2013) | Hospital readmissions |
|  |  | Tyler et al. (2023) | Readmission 30 days |
|  |  | Chartrand et al. (2023) | 28-Readmission |
|  |  | Bonetti et al. (2021) | One-month readmission rates |
|  |  | Daliri et al. (2021) | Readmission |
|  |  | Mekonnen et al. (a) (2016) | 1 month readmission |
|  |  | Hansen et al. (2011) | 30-day readmission |

| **Outcome** | **Included in SoE?** | **Review** | **Outcome description in review** |
| --- | --- | --- | --- |
| **Readmission (30-day)** *(continued)* | *Yes* | Becker et al. (2021) | 30-day readmission rates |
|  |  | Gillespie et al. (2023) | Readmission 30 days |
|  |  | Oh et al. (2023) | 30-day readmissions |
|  |  | Stamp et al. (2014) | Hospital readmission rates: short-term (30) |
|  |  | Weeda et al. (2023) | Hospital readmission |
| **Readmission (90-day)** | *Yes* | Bonetti et al. (2021) | Readmission 3 Month |
|  |  | Chartrand et al. (2023) | Hospital readmissions |
|  |  | Lee et al. (2022) | 3 months |
|  |  | Rodakowski et al. (2017) | Readmission rates (90 days) |
|  |  | Stamp et al. (2014) | Readmission 90 days |
|  |  | Tyler et al. (2023) | 90-day readmission |
| **Readmission (180-day)** | *Yes* | Allen et al. (2014) | Re-hospitalization at up to 6 months |
|  |  | Bonetti et al. (2021) | Readmission 6 months |
|  |  | Chartrand et al. (2023) | Hospital readmissions (180 d) |
|  |  | Lee et al. (2022) | 6 months readmission |
|  |  | Rodakowski et al. (2017) | Readmission rates (180 days) |
|  |  | Stamp et al. (2014) | Readmission 180 days |
|  |  | Tyler et al. (2023) | 180-day readmission |
|  |  | Verhaegh et al. (2014) | Hospital readmission rates: intermediate-term (31–180 days) |
| **Readmission (1-year)** | *Yes* | Lee et al. (2022) | 12 months readmission |
|  |  | Verhaegh et al. (2014) | Hospital readmission rates: long-term (181–365 days) |
| **Revisits (readmission and ED-visits** | *Yes* | Hammad et al. (2017) | Readmission and emergency visit rate |
|  |  | Mekonnen et al. (a) (2016) | Composite rate of readmissions and / or ED visits |
|  |  | Skjøt-Arkil et al. (2018) | Hospital visits (30, 90, days, 12 months) |

# **S6: Calculation of Strength of Evidence (SoE)**

Determination of weights, consisting of baseline weight and three additional criteria

| **Criteria** | | Outcome synthesized narratively  *(no meta-analysis)* | Outcome analyzed in a **meta-analysis** |
| --- | --- | --- | --- |
|  | **Baseline weight** | ***b_ij_=1*** | |
| *1* | Meta-analysis conducted | *-* | *a_ij_=1* |
| *2* | Number of primary studies ≥5 | *a_ij_=1* | *-* |
| *3* | High AMSTAR2 rating | *a_ij_=1* | *a_ij_=1* |

# **S7: Excluded studies including exclusion reason**

| **No.** | **Title** | **Authors** | **Exclusion Reason** | **Exclusion Category** |
| --- | --- | --- | --- | --- |
| 1 | Effect of health information technology (HIT)-based discharge transition interventions on patient readmissions and emergency room visits: a systematic review | Abraham et al. (2022) | PICO not given (no comparator) | PICOS not fulfilled |
| 2 | Intervention effectiveness by pharmacists integrated within an interdisciplinary health team on chronic complex patients | Acosta-García et al. (2020) | No DP focus | No DP focus |
| 3 | Effectiveness of Transitional Care Interventions for Heart Failure Patients: A Systematic Review With Meta-Analysis | Al Sattouf et al. (2022) | No DP focus | No DP focus |
| 4 | The medication reconciliation process and classification of discrepancies: a systematic review | Almanasreh et al. (2016) | No (relevant) outcomes reported | No (relevant) outcomes reported |
| 5 | Stroke rehabilitation services to accelerate hospital discharge and provide home-based care: an overview and cost analysis | Anderson et al. (2002) | No DP focus | No DP focus |
| 6 | Evidenced-based factors in readmission of patients with heart failure | Anderson et al. (2006) | No DP focus | No DP focus |
| 7 | Patient education in the management of coronary heart disease | Anderson et al. (2017) | No DP focus | No DP focus |
| 8 | A review to access knowledge, attitudes, and practices among nurses of cardiac medicine for heart failure patients to prevent readmission in hospitals | Anny and Ani (2023) | No (relevant) outcomes reported | No (relevant) outcomes reported |
| 9 | The transition of palliative care from the hospital to the home: a narrative review of experiences of patients and family caretakers | Arias et al. (2015) | Patient group (palliative focus) | PICOS not fulfilled |
| 10 | Perceived Self-Efficacy, Confidence, and Skill Among Factors of Adult Patient Participation in Transitional Care: A Systematic Review of Quantitative Studies | Bailey et al. (2022) | Interventions unclear | Other |
| 11 | A systematic and critical review of the literature: the effectiveness of Occupational Therapy Home Assessment on a range of outcome measures | Barras (2005) | No DP focus | No DP focus |
| 12 | Improving uptake and adherence in cardiac rehabilitation: literature review | Beswick et al. (2005) | No DP focus | No DP focus |
| 13 | Transitional care after hospitalization for acute stroke or myocardial infarction: a systematic review | Bettger et al. (2012) | No DP focus | No DP focus |
| 14 | Interventions to Reduce Hospital Readmissions in Older African Americans: A Systematic Review of Studies Including African American Patients | Bhandari et al. (2023) | No DP focus | No DP focus |
| 15 | Effectiveness of discharge interventions from hospital to home on hospital readmissions: a systematic review | Braet et al. (2016) | No DP focus | No DP focus |
| 16 | Medication Counselling in Older Patients Prior to Hospital Discharge: A Systematic Review | Capiau et al. (2020) | PICO not given (no comparator) | PICOS not fulfilled |
| 17 | Hospital Palliative Care Teams and Post-Acute Care in Nursing Facilities | Carpenter et al. (2017) | Patient group (palliative focus) | PICOS not fulfilled |
| 18 | End‐of‐life care pathways for improving outcomes in caring for the dying | Chan et al. (2016) | Patient group (palliative focus) | PICOS not fulfilled |
| 19 | Effects of continuity of care on health outcomes among patients with diabetes mellitus and/or hypertension: a systematic review | Chan et al. (2021) | No DP focus | No DP focus |
| 20 | Does hospital-based transitional care reduce the postoperative complication in patients with enterostomy? A meta-analysis | Chen et al. (2016) | Interventions unclear | Other |
| 21 | Enablers and barriers in hospital-to-home transitional care for stroke survivors and caregivers: A systematic review | Chen et al. (2021) | Study type (qualitative) | Wrong study type |
| 22 | A systematic review of nurse-assisted case management to improve hospital discharge transition outcomes for the elderly | Chiu et al. (2007) | No DP focus | No DP focus |
| 23 | A qualitative evidence synthesis exploring people after stroke, family members, carers and healthcare professionals' experiences of early supported discharge (ESD) after stroke | Connor et al. (2023) | Study type (qualitative) | Wrong study type |
| 24 | A systematic review of comprehensive geriatric assessment to improve outcomes for frail older people being rapidly discharged from acute hospital: 'interface geriatrics' | Conroy et al. (2011) | No DP focus | No DP focus |
| 25 | A systematic review on the effect of the organisation of hospital discharge on patient health outcomes | Couturier et al. (2016) | PICO not given (no comparator) | PICOS not fulfilled |
| 26 | The impact of facility-based transitional care programs on function and discharge destination for older adults with cognitive impairment: a systematic review | Cumal et al. (2022) | No DP focus | No DP focus |
| 27 | Medication review interventions to reduce hospital readmissions in older people | Dautzenberg et al. (2021) | Interventions unclear | Other |
| 28 | Effectiveness of nurse-led services for people with chronic disease in achieving an outcome of continuity of care at the primary-secondary healthcare interface: A quantitative systematic review | Davis et al. (2021) | Setting not (primarily) in-hospital | PICOS not fulfilled |
| 29 | Do palliative care interventions reduce emergency department visits among patients with cancer at the end of life? A systematic review | DiMartino et al. (2014) | Patient group (palliative focus) | PICOS not fulfilled |
| 30 | Transitional care of older ethnic minority patients: An integrative review | Dolu et al. | No (relevant) outcomes reported | No (relevant) outcomes reported |
| 31 | Effectiveness of structured discharge process in reducing hospital readmission of adult patients with community acquired pneumonia: A systematic review | Domingo et al. (2012) | Study type (protocol) | Wrong study type |
| 32 | Strategies for utilisation management of hospital services: a systematic review of interventions | Doshmangir et al. (2022) | No DP focus | No DP focus |
| 33 | Interventions to Prevent Readmissions in Hospitalized Peripheral Vascular Surgery Patients: A Systematic Review | Doss et al. (2023) | No DP focus | No DP focus |
| 34 | Early Supported Discharge/Hospital At Home For Acute Exacerbation of Chronic Obstructive Pulmonary Disease: A Review and Meta-Analysis | Echevarria et al. (2016) | No DP focus | No DP focus |
| 35 | Effectiveness of discharge interventions on readmissions for patients with chronic obstructive pulmonary disease: a systematic review protocol | Ersgard et al. (2014) | Study type (protocol) | Wrong study type |
| 36 | Continuity of care interventions for preventing hospital readmission of older people with chronic diseases: A meta-analysis | Facchinetti et al. (2020) | No DP focus | No DP focus |
| 37 | Services for reducing duration of hospital care for acute stroke patients | Fearon et al. (2012) | No DP focus | No DP focus |
| 38 | Care Transition from hospital to home: Integrative Review | Feil et al. (2017) | No (relevant) outcomes reported | No (relevant) outcomes reported |
| 39 | Pharmacist-led medication reconciliation at patient discharge: A scoping review | Fernandes et al. (2020) | No (relevant) outcomes reported | No (relevant) outcomes reported |
| 40 | A scoping review of models of care for the management of older trauma patients | Ferrah et al. (2024) | Study type (scoping review) | Wrong study type |
| 41 | Impact of pharmacist and physician collaborations in primary care on reducing readmission to hospital: A systematic review and meta-analysis | Foot et al. (2022) | No DP focus | No DP focus |
| 42 | Effectiveness of acute geriatric unit care using acute care for elders components: a systematic review and meta-analysis | Fox et al. (2012) | No DP focus | No DP focus |
| 43 | Acute care for elders components of acute geriatric unit care: systematic descriptive review | Fox et al. (2013) | No DP focus | No DP focus |
| 44 | Barriers and Facilitators to Implementing Interventions for Reducing Avoidable Hospital Readmission: Systematic Review of Qualitative Studies | Fu et al. (2023) | Study type (qualitative) | Wrong study type |
| 45 | Transitional care programs to improve outcomes in patients with traumatic brain injury and their caregivers: A systematic review and meta-analysis | Ganefianty et al. (2021) | No DP focus | No DP focus |
| 46 | Outcomes of transitional care programs on adolescent chronic inflammatory systemic diseases: systematic review and meta-analyses | García-Rodríguez et al. (2022) | Patient group (children) | PICOS not fulfilled |
| 47 | Nurse-led Care for Patients with Rheumatoid Arthritis: A Systematic Review of the Effect on Quality of Care | Garner et al. (2017) | No DP focus | No DP focus |
| 48 | What interventions keep older people out of nursing homes? A systematic review and meta-analysis | Gaugler et al. (2023) | Interventions unclear | Other |
| 49 | European Society of Cardiology quality indicators for the cardiovascular pre-operative assessment and management of patients considered for non-cardiac surgery. Developed in collaboration with the European Society of Anaesthesiology and Intensive Care | Gencer et al. (2023) | No (relevant) outcomes reported | No (relevant) outcomes reported |
| 50 | Patient Navigator Program in Oncology Clinical Practice | George et al. (2018) | No DP focus | No DP focus |
| 51 | Safety of early discharge after primary angioplasty in low-risk patients with ST-segment elevation myocardial infarction: A meta-analysis of randomised controlled trials | Gong et al. (2018) | No DP focus | No DP focus |
| 52 | Effectiveness of intermediate care in nursing-led in-patient units | Griffiths et al. (2007) | No DP focus | No DP focus |
| 53 | Community services' involvement in the discharge of older adults from hospital into the community | Guerin et al. (2013) | No (relevant) outcomes reported | No (relevant) outcomes reported |
| 54 | Effectiveness of facility-based transition care on health-related outcomes for older adults: A systematic review and meta-analysis | Hang et al. (2021) | Interventions unclear | Other |
| 55 | A Systematic Review of Patient Safety Measures in Adult Primary Care | Hatoun et al. (2017) | No DP focus | No DP focus |
| 56 | Transition of patients with recently diagnosed Dementia from inpatient to outpatient setting- a scoping review | Hegerath et al. (2024) | Study type (qualitative) | Wrong study type |
| 57 | Clinical implementation of systematic medication reconciliation and review as part of the Lund Integrated Medicines Management model - impact on all-cause emergency department revisits | Hellström et al. (2012) | Study type (primary study) | Wrong study type |
| 58 | Impact of informational and relational continuity for people with palliative care needs: a mixed methods rapid review | Hudson et al. (2018) | Patient group (palliative focus) | PICOS not fulfilled |
| 59 | Is case management effective in reducing the risk of unplanned hospital admissions for older people? A systematic review and meta-analysis | Huntley et al. (2013) | No DP focus | No DP focus |
| 60 | Which features of primary care affect unscheduled secondary care use? A systematic review | Huntley et al. (2014) | No DP focus | No DP focus |
| 61 | Does case management for patients with heart failure based in the community reduce unplanned hospital admissions? A systematic review and meta-analysis | Huntley et al. (2016) | No DP focus | No DP focus |
| 62 | Specialist nurse-initiated interventions in breast cancer care: A systematic review of randomised controlled trials | Hussain et al. (2020) | No DP focus | No DP focus |
| 63 | The effects of supporting discharge from hospital to home in older people | Hyde et al. (2000) | No DP focus | No DP focus |
| 64 | Efficacy of acute care pathways for older patients: a systematic review and meta-analysis | Ijadi et al. (2022) | No DP focus | No DP focus |
| 65 | Implications of Transitional Care Interventions on Hospital Readmissions in Patients With Destination Therapy Left Ventricular Assist Devices | Iseler et al. (2019) | No DP focus | No DP focus |
| 66 | Transitions in care between hospital and community settings for individuals with a substance use disorder: A systematic review | James et al. (2023) | Patient group (psychiatric focus) | PICOS not fulfilled |
| 67 | Early Supported Discharge and Transitional Care Management After Stroke: A Systematic Review and Meta-Analysis | Jee et al. (2022) | No DP focus | No DP focus |
| 68 | Continuity of Care in Chronic Diseases: A Concept Analysis by Literature Review | Jingjing et al. (2020) | Study type (no systematic review) | Wrong study type |
| 69 | An integrative review of nurse-led community-based case management effectiveness | Joo et al. (2014) | No DP focus | No DP focus |
| 70 | Case management effectiveness in reducing hospital use: a systematic review | Joo et al. (2017) | No DP focus | No DP focus |
| 71 | Effectiveness of transitional care interventions for chronic illnesses: A systematic review of reviews | Joo et al. (2021) | Study type (Umbrella review) | Wrong study type |
| 72 | The Effectiveness of Transitions-of-Care Interventions in Reducing Hospital Readmissions and Mortality: A Systematic Review | Kamermayer et al. (2017) | Interventions unclear | Other |
| 73 | Discharge education delivered to general surgical patients in their management of recovery post discharge: A systematic mixed studies review | Kang et al. (2018) | No (relevant) outcomes reported | No (relevant) outcomes reported |
| 74 | So many options, where do we start? An overview of the care transitions literature | Kansagara et al. (2016) | Study type (umbrella review) | Wrong study type |
| 75 | Review of successful hospital readmission reduction strategies and the role of health information exchange | Kash et al. (2017) | No DP focus | No DP focus |
| 76 | How Leading Hospitals Operationalize Evidence-Based Readmission Reduction Strategies: A Mixed-Methods Comparative Study Using Systematic Review and Survey Design | Kash et al. (2019) | Study type (mixed methods) | Wrong study type |
| 77 | Economic evidence with respect to cost-effectiveness of the transitional care model among geriatric patients discharged from hospital to home: a systematic review | Kast et al. (2021) | Too few primary studies | Other |
| 78 | A systematic review of the cost-effectiveness of noncardiac transitional care units | Keenan et al. (1998) | No DP focus | No DP focus |
| 79 | Palliative care transitions from acute care to community-based care: A qualitative systematic review of the experiences and perspectives of health care providers | Killackey et al. (2020) | Study type (qualitative) | Wrong study type |
| 80 | Advanced Medication Reconciliation: A Systematic Review of the Impact on Medication Errors and Adverse Drug Events Associated with Transitions of Care | Killin et al. (2021) | PICO not given (not usual care) | PICOS not fulfilled |
| 81 | Effects, barriers and facilitators in predischarge home assessments to improve the transition of care from the inpatient care to home in adult patients: an integrative review | Kirchner et al. (2021) | No DP focus | No DP focus |
| 82 | The developing role of community pharmacists in facilitating care transitions: A systematic review | Kooyman et al. (2019) | No (relevant) outcomes reported | No (relevant) outcomes reported |
| 83 | Effectiveness of discharge education strategies versus usual care on clinical outcomes in acute coronary syndrome patients: a systematic review | Kourbelis et al. (2020) | No Access | Other |
| 84 | A Call for Interfacing Measures of Instrumental Activities of Daily Living Across the Transition of Care | Koyfman et al. (2019) | Study type (no systematic review) | Wrong study type |
| 85 | Deficits in communication and information transfer between hospital-based and primary care physicians: implications for patient safety and continuity of care | Kripalani et al. (2007) | PICO not given (not usual care) | PICOS not fulfilled |
| 86 | A synthesis of the secondary literature on effectiveness of hospital avoidance and discharge programs | Kumar et al. (2007) | Study type (no systematic review) | Wrong study type |
| 87 | Medication reconciliation during transitions of care as a patient safety strategy: a systematic review | Kwan et al. (2013) | No DP focus | No DP focus |
| 88 | Interventions to improve transitional care between nursing homes and hospitals: a systematic review | LaMantia et al. (2010) | Setting not (primarily) in-hospital | PICOS not fulfilled |
| 89 | Effect of inpatient rehabilitation treatment ingredients on functioning, quality of life, length of stay, discharge destination, and mortality among older adults with unplanned admission: an overview review | Lambe et al. (2022) | No DP focus | No DP focus |
| 90 | Early supported discharge services for stroke patients: a meta-analysis of individual patients' data | Langhorne et al. (2005) | No DP focus | No DP focus |
| 91 | Early supported discharge services for people with acute stroke | Langhorne et al. (2017) | No DP focus | No DP focus |
| 92 | Interventions to improve patient safety in transitional care - a review of the evidence | Laugaland et al. (2012) | No DP focus | No DP focus |
| 93 | Impact of Transitional Care Services for Chronically Ill Older Patients: A Systematic Evidence Review | Le Berre et al. (2017) | No DP focus | No DP focus |
| 94 | Effectiveness of Discharge Education With the Teach-Back Method on 30-Day Readmission: A Systematic Review | Lee et al. (2021) | Too few primary studies | Other |
| 95 | A Systematic Review of Criteria-Led Patient Discharge | Lees-Deutsch et al. (2019) | Interventions unclear | Other |
| 96 | Impact of Medication Reconciliation and Review on Clinical Outcomes | Lehnbom et al. (2014) | No DP focus | No DP focus |
| 97 | Clinical and organizational content of clinical pathways for digestive surgery: a systematic review | Lemmens et al. (2009) | No DP focus | No DP focus |
| 98 | Preventing 30-day hospital readmissions: a systematic review and meta-analysis of randomized trials | Leppin et al. (2014) | No DP focus | No DP focus |
| 99 | Caregiver Engagement Enhances Outcomes Among Randomized Control Trials of Transitional Care Interventions: A Systematic Review and Meta-analysis | Levoy et al. (2022) | PICO not given (no comparator) | PICOS not fulfilled |
| 100 | The effectiveness of transitional care interventions for adult people with heart failure on patient-centered health outcomes: A systematic review and meta-analysis including dose-response relationship | Li et al. (2021) | No DP focus | No DP focus |
| 101 | Effect of Transitional Care Strategies on Health-Related Quality of Life (HRQoL) in Heart Failure with Reduced Ejection Fraction (HFrEF): A Systematic Review and Meta-analysis of Randomized Controlled Trails | Li et al. (2024) | No DP focus | No DP focus |
| 102 | The effect of transition care interventions incorporating health coaching strategies for stroke survivors: A systematic review and meta-analysis | Lin et al. (2020) | No DP focus | No DP focus |
| 103 | Interventions to reduce hospital readmissions in the elderly: in-hospital or home care. A systematic review | Linertová et al. (2011) | No DP focus | No DP focus |
| 104 | Effectiveness of Pharmacological-Based Interventions, Including Education and Prescribing Strategies, to Reduce Subacute Pain After Total Hip or Knee Arthroplasty: A Systematic Review of Randomized Controlled Trials | Liu et al. (2022) | No DP focus | No DP focus |
| 105 | Pre-discharge home assessment visits in assisting patients' return to community living: A systematic review and meta-analysis | Lockwood et al. (2015) | PICO not given (no comparator); not in-hospital | PICOS not fulfilled |
| 106 | Factors associated with readmission in chronic kidney disease: Systematic review and meta-analysis | Low et al. (2023) | No DP focus | No DP focus |
| 107 | Effectiveness of nursing discharge planning interventions on health-related outcomes in discharged elderly inpatients: a systematic review | Mabire et al. (2016) | Study type (protocol) | Wrong study type |
| 108 | Transition of care for stroke patients: an integrative review | Magagnin et al. (2022) | Study type (qualitative) | Wrong study type |
| 109 | Family involvement in managing medications of older patients across transitions of care: a systematic review | Manias et al. (2019) | Study type (mixed methods) | Wrong study type |
| 110 | Effectiveness of nurse‐led discharge service on adult surgical inpatients: A meta‐analysis of randomized controlled trials | Mao et al. (2022) | No DP focus | No DP focus |
| 111 | The role of the pharmacist in the hospital discharge of cancer patients: an integrative review | Mattos et al. (2023) | No (relevant) outcomes reported | No (relevant) outcomes reported |
| 112 | Improving Care Transitions in Patients with Heart Failure: An Integrative Literature Review | McLain (2018) | No DP focus | No DP focus |
| 113 | Effectiveness of patient-caregiver dyad discharge interventions on hospital readmissions of elderly patients with community acquired pneumonia: a systematic review | McLeod et al. (2011) | Too few primary studies | Other |
| 114 | Discharge planning in chronic conditions: an evidence-based analysis | McMartin (2013) | Study type (umbrella review) | Wrong study type |
| 115 | Systematic review and meta-analysis of the effectiveness of pharmacist-led medication reconciliation in the community after hospital discharge | McNab et al. (2018) | No DP focus | No DP focus |
| 116 | Nurse-led intervention for the management of bariatric surgery patients: A systematic review | Mendes et al. (2023) | No DP focus | No DP focus |
| 117 | Defining Delayed Discharges of Inpatients and Their Impact in Acute Hospital Care: A Scoping Review | Micallef et al. (2022) | No DP focus | No DP focus |
| 118 | Medication Reconciliation at Discharge from Hospital: A Systematic Review of the Quantitative Literature | Michaelsen et al. (2015) | No DP focus | No DP focus |
| 119 | Effectiveness of hospital-to-home transitional care interventions and consultation for implementation in Sudan: a scoping review of systematic reviews | Mohamedsharif et al. (2023) | Study type (umbrella review) | Wrong study type |
| 120 | Nurse practitioner-led transitional care interventions: An integrative review | Mora et al. (2017) | No DP focus | No DP focus |
| 121 | Components of the transitional care model (TCM) to reduce readmission in geriatric patients: a systematic review | Morkisch et al. (2020) | No DP focus | No DP focus |
| 122 | Hospital-Based Medication Reconciliation Practices A Systematic Review | Mueller et al. (2012) | No DP focus | No DP focus |
| 123 | A systematic review of the role of community pharmacies in improving the transition from secondary to primary care | Nazar et al. (2015) | Setting not (primarily) in-hospital | PICOS not fulfilled |
| 124 | Discharge communication practices and healthcare provider and patient preferences, satisfaction and comprehension: A systematic review | Newnham et al. (2017) | Study type (mixed methods) | Wrong study type |
| 125 | Systematic Review of Ostomy Care Pathways | Nizum et al. (2022) | PICO not given (no comparator) | PICOS not fulfilled |
| 126 | Enhancing potential impact of hospital discharge interventions for patients with COPD: a qualitative systematic review | Nygård et al. (2023) | Study Type (qualitative) | Wrong study type |
| 127 | The Effectiveness of Transition Interventions to Support Older Patients From Hospital to Home: A Systematic Scoping Review | O'Donnell et al. (2021) | No DP focus | No DP focus |
| 128 | The effect of transitions intervention to ensure patient safety and satisfaction when transferred from hospital to home health care-A systematic review | Oksholm et al. (2023) | No DP focus | No DP focus |
| 129 | Effectiveness of Pharmacist Intervention to Reduce Medication Errors and Health-Care Resources Utilization After Transitions of Care: A Meta-analysis of Randomized Controlled Trials | Oliveira et al. (2021) | No (relevant) outcomes reported | No (relevant) outcomes reported |
| 130 | Transition of care for acute stroke and myocardial infarction patients: from hospitalization to rehabilitation, recovery, and secondary prevention | Olson et al. (2011) | No DP focus | No DP focus |
| 131 | Effectiveness of acute geriatric unit care on functional decline, clinical and process outcomes among hospitalised older adults with acute medical complaints: a systematic review and meta-analysis | O'Shaughnessy et al. (2022) | No DP focus | No DP focus |
| 132 | A systematic review of the effectiveness of discharge care bundles for patients with COPD | Ospina et al. (2017) | Too few primary studies | Other |
| 133 | Transitions of care interventions to improve quality of life among patients hospitalized with acute conditions: a systematic literature review | Oyesanya et al. (2021) | Study type (qualitative) | Wrong study type |
| 134 | A systematic review of older patients' experiences and perceptions of communication about managing medication across transitions of care | Ozavci et al. (2021) | No (relevant) outcomes reported | No (relevant) outcomes reported |
| 135 | Can non-pharmacological interventions reduce hospital admissions in people with dementia? A systematic review | Packer  et al. (2019) | No DP focus | No DP focus |
| 136 | A systematic review of discharge arrangements for older people | Parker (2002) | No DP focus | No DP focus |
| 137 | Interventions to optimise transitional care coordination for older people living with dementia and concomitant multimorbidity and their caregivers: A systematic review | Parker et al. (2020) | No DP focus | No DP focus |
| 138 | Discharge planning from hospital to home | Parkes et al. (2000) | Latest version of paper considered only | Other |
| 139 | A state‐of‐the‐art review of the experience of care coordination interventions for people living with multimorbidity | Peart et al. (2020) | Study type (qualitative) | Wrong study type |
| 140 | Continuity of care with doctors-a matter of life and death? A systematic review of continuity of care and mortality | Pereira et al. (2018) | No DP focus | No DP focus |
| 141 | Transitional care programs: who is left behind? A systematic review | Piraino et al. (2012) | No (relevant) outcomes reported | No (relevant) outcomes reported |
| 142 | Effectiveness of nurse-led disease management programs on health outcomes and health service utilization in adult patients with chronic obstructive pulmonary disease: A systematic review protocol | Poon at al. (2013) | Study type (protocol) | Wrong study type |
| 143 | Effectiveness of self-management interventions during the peri-hospitalization period in patients with stroke: A systematic review and meta-analysis | Prados-Román et al. (2024) | No DP focus | No DP focus |
| 144 | Discharge planning from hospital to home for elderly patients: a meta-analysis | Preyde et al. (2009) | PICO not given (no comparator) | PICOS not fulfilled |
| 145 | A review of the early discharge experiences of stroke survivors and their carers | Pringle et al. (2008) | No DP focus | No DP focus |
| 146 | Effectiveness of dyadic interventions to improve stroke patient-caregiver dyads' outcomes after discharge: A systematic review and meta-analysis study | Pucciarelli et al. (2021) | No DP focus | No DP focus |
| 147 | Factors affecting hospital readmission rates following an acute coronary syndrome: A systematic review | Rashidi et al. (2022) | No (relevant) outcomes reported | No (relevant) outcomes reported |
| 148 | Impact of medication reconciliation for improving transitions of care | Redmond et al. (2018) | No DP focus | No DP focus |
| 149 | The Nurse Led of stroke patient after discharge from hospital. A Systematic Review and GRADE | Rega et al. (2020) | No DP focus | No DP focus |
| 150 | The role of hospitals in bridging the care continuum: a systematic review of coordination of care and follow-up for adults with chronic conditions | Regge et al. (2017) | No DP focus | No DP focus |
| 151 | The impact of interventions on management of frailty in hospitalized frail older adults: a systematic review and meta-analysis | Rezaei et al. (2020) | No DP focus | No DP focus |
| 152 | Review: comprehensive discharge planning plus post-discharge support reduced total readmissions in older patients with congestive heart failure | Rideout (2004) | Study type (comment) | Wrong study type |
| 153 | Systematic Review of Ambulatory Transitional Care Management (TCM) Visits on Hospital 30-Day Readmission Rates | Roper et al. (2017) | No DP focus | No DP focus |
| 154 | Approaches to evaluating efficiency and quality of integrated health care: state of the evidence | Rudawska (2016) | No DP focus | No DP focus |
| 155 | Transitions in Atrial Fibrillation Care: A Systematic Review | Rush et al. (2020) | No DP focus | No DP focus |
| 156 | Case management for integrated care of older people with frailty in community settings | Sadler et al. (2023) | No DP focus | No DP focus |
| 157 | The costs, resource use and cost-effectiveness of Clinical Nurse Specialist-led interventions for patients with palliative care needs: A systematic review of international evidence | Salamanca-Balen et al. (2018) | No DP focus | No DP focus |
| 158 | The beneficial effects of transitional care for patients with stroke: A meta-analysis | Saragih et al. (2024) | No DP focus | No DP focus |
| 159 | Palliative Care Transitions From Acute Care to Community-Based Care-A Systematic Review | Saunders et al. (2019) | No DP focus | No DP focus |
| 160 | A Review: Discharge Navigation and Its Effect on Heart Failure Readmissions | Schell (2014) | Study type (no systematic review) | Wrong study type |
| 161 | A systematic literature review and narrative synthesis on the risks of medical discharge letters for patients' safety | Schwarz et al. (2019) | No (relevant) outcomes reported | No (relevant) outcomes reported |
| 162 | Does inpatient palliative care consultation impact outcomes following hospital discharge? A narrative systematic review | Scott et al. (2020) | No DP focus | No DP focus |
| 163 | A structured review of chronic care model components supporting transition between healthcare service delivery types for older people with multiple chronic diseases | Sendall et al. (2017) | No DP focus | No DP focus |
| 164 | In older LTC residents, transitional care interventions vs. usual care reduce hospital readmissions | Sewell (2020) | Study type (comment) | Wrong study type |
| 165 | Transitional care: Concept analysis using Rodgers' evolutionary approach | Shahsavari et al. (2019) | Study type (qualitative) | Wrong study type |
| 166 | Methods and Effectiveness of Communication Between Hospital Allied Health and Primary Care Practitioners: A Systematic Narrative Review | Sheehan et al. (2021) | Study type (qualitative) | Wrong study type |
| 167 | Discharge planning from hospital to home | Shepperd et al. (2013) | Latest version of paper considered only | Other |
| 168 | Discharge planning from hospital to home | Shepperd et al. (2013) | Latest version of paper considered only | Other |
| 169 | Discharge planning from hospital to home | Shepperd et al. (2013) | Latest version of paper considered only | Other |
| 170 | Towards a patient journey perspective on causes of unplanned readmissions using a classification framework: results of a systematic review with narrative synthesis | Singotani et al. (2019) | Study type (qualitative) | Wrong study type |
| 171 | A systematic review of the effectiveness of nurse coordinated transitioning of care on readmission rates for patients with heart failure | Slyer et al. (2011) | No DP focus | No DP focus |
| 172 | Effectiveness of different nursing handover styles for ensuring continuity of information in hospitalised patients | Smeulers et al. (2014) | No DP focus | No DP focus |
| 173 | Interventions for reducing hospital-associated deconditioning: A systematic review and meta-analysis | Smith et al. (2020) | No DP focus | No DP focus |
| 174 | The effectiveness of tele-transitions of care interventions in high-risk older adults: A systematic review and meta-analysis | Soh et al. (2023) | No DP focus | No DP focus |
| 175 | Approaches for improving continuity of care in medication management: a systematic review | Spinewine et al. (2013) | No DP focus | No DP focus |
| 176 | Advancing Complex Case Management Competencies in a Health Care System | Stark (2020) | Study type (no systematic review) | Wrong study type |
| 177 | Effects of comprehensive geriatric care on depressive symptoms, emergency department visits, re-hospitalization and discharge to the same residence in older persons receiving hip-fracture surgery: A meta-analysis | Su et al. (2022) | No DP focus | No DP focus |
| 178 | The Effectiveness of Transition Care Interventions from Hospital to Home on Rehospitalization in Older Patients with Heart Failure: An Integrative Review | Suksatan et al. (2022) | No DP focus | No DP focus |
| 179 | Facilitators and inhibitors in hospital-to-home transitional care for elderly patients with chronic diseases: A meta-synthesis of qualitative studies | Sun et al. (2023) | Study type (qualitative) | Wrong study type |
| 180 | Disease management interventions for heart failure | Takeda et al. (2019) | No DP focus | No DP focus |
| 181 | Family involvement in transitional care from hospital to home and its impact on older patients, families, and health care providers: a mixed methods systematic review protocol | Thiengtham et al. (2022) | Study type (protocol) | Wrong study type |
| 182 | What health outcomes matter to frail older people? | Tipping et al. (2020) | Study type (qualitative) | Wrong study type |
| 183 | Transitional care of older adults in skilled nursing facilities: A systematic review | Toles et al. (2016) | No DP focus | No DP focus |
| 184 | A Literature Review on the Benefits for an Interprofessional Educational Program to Increase Novice Nurse Awareness of Case Management in Heart Failure | Trefethen et al. (2021) | No DP focus | No DP focus |
| 185 | Effectiveness of quality improvement strategies for coordination of care to reduce use of health care services: a systematic review and meta-analysis | Tricco et al. (2014) | No DP focus | No DP focus |
| 186 | Effectiveness of care transition strategies for colorectal cancer patients: a systematic review and meta-analysis | Trindade et al. (2022) | No DP focus | No DP focus |
| 187 | A Systematic Review of the Effects of Community Transition Programs on Quality of Life and Hospital Readmissions for Adults With Traumatic Spinal Cord Injury | Tschoepe et al. (2022) | No DP focus | No DP focus |
| 188 | Evidence of effectiveness of hospital transition care in the elderly: rapid systematic review | Uchimura et al. (2023) | No DP focus | No DP focus |
| 189 | Drug-Related Problems in the transitional care of the elderly from hospital to home | Valente et al. (2019) | Study type (qualitative) | Wrong study type |
| 190 | Clinical Pharmacy Services in Older Inpatients: An Evidence-Based Review | Van der Linden et al. (2020) | No DP focus | No DP focus |
| 191 | Measurement tools and outcome measures used in transitional patient safety; a systematic review | Van Melle et al. (2018) | No (relevant) outcomes reported | No (relevant) outcomes reported |
| 192 | Comparative effectiveness of transitional care services in patients discharged from the hospital with heart failure: a systematic review and network meta-analysis | Van Spall et al. (2017) | PICO not given (comparator not usual care) | PICOS not fulfilled |
| 193 | The association between continuity of care and outcomes: a systematic and critical review | Van Walraven et al. (2010) | No DP focus | No DP focus |
| 194 | Transitional Care for Patients With Congestive Heart Failure: A Systematic Review and Meta-Analysis | Vedel et al. (2015) | No DP focus | No DP focus |
| 195 | The impact of transitional care programs on health services utilization in community-dwelling older adults: a systematic review | Weeks et al. (2018) | No DP focus | No DP focus |
| 196 | Early supported discharge for older adults admitted to hospital with medical complaints: a systematic review and meta-analysis | Williams et al. (2022) | No DP focus | No DP focus |
| 197 | Early supported discharge for older adults admitted to hospital after orthopaedic surgery: a systematic review and meta-analysis | Williams et al. (2024) | No DP focus | No DP focus |
| 198 | Continuity of Care to Prevent Readmissions for Patients with Chronic Obstructive Pulmonary Disease: A Systematic Review and Meta-Analysis | Yang et al. (2017) | No DP focus | No DP focus |
| 199 | Measurement tools that assess the quality of transitional care from patients' perspective: A literature review | Yoshimura et al. (2022) | No (relevant) outcomes reported | No (relevant) outcomes reported |
| 200 | Disease management programmes for older people with heart failure: crucial characteristics which improve post-discharge outcomes | Yu et al. (2006) | No DP focus | No DP focus |
| 201 | Peri-discharge complex interventions for reducing 30-day hospital readmissions among heart failure patients: overview of systematic reviews and network meta-analysis | Zhong et al. (2022) | PICO not given (comparator not usual care) | PICOS not fulfilled |
| 202 | The benefits of transitional care in older patients with chronic diseases: a systematic review and meta-analysis | Zou et al. (2022) | No DP focus | No DP focus |

# **S8: Study characteristics**

| **Review** | **Study goal** | **Included studies** (no. of studies/no. of total records screened, search horizon, study types) | **Participants**  (number; characteristics/ condition) | **Inclusion criteria^1^**  (structured according to PICOS) | **Exclusion criteria^1^** | **Subgroup analysis:**  Subgroup methodology & subgroups types | **Study conclusion** |
| --- | --- | --- | --- | --- | --- | --- | --- |
| **Albert et al. (2016)** | To evaluate existing transition-of-care models and identify common themes that may reduce exacerbations and rehospitalizations, and improve quality of life in patients with heart failure | 24/n.a.  1990–2015  RCTs (n=9)  Cluster RCTs (n=6)  Quasi-experimental (n=4)  Prospective (n=2)  Retrospective (n=1)  Case studies (n=1) | 26,647 patients    Patients with heart failure | - Population: At least some patients with heart failure - Intervention: Included at least one transition component between care settings; intervention had to be delivered in North America - Comparator: Usual care | n.a. | **Method:**  (5) Results described but not compared (not considered in subgroup synthesis) | Many [transitional care models] that used a bundled approach led to reduced rehospitalization and emergency care, increased post-discharge follow-up, and improved quality of life and cost savings |
| **Allen et al. (2014)** | Identify and synthesize research on the quality of transitional care interventions for older people with chronic illnesses and make recommendations for research and practice | 12/405  1990-05/2013  RCT (n=12) | 5,269 patients    ≥60 years | - Population: Older people (≥60 years) - Intervention: Transitional care intervention compared with standard hospital discharge; transitional care included any intervention applied in the inpatient setting, inclusive of follow-up in the community - Comparator: Standard hospital discharge - Outcomes: Outcomes that evaluated quality indicators related to older people. (≥60 years) - Study types: RCTs | n.a. | **Method:**  (5) Results described but not compared (not considered in subgroup synthesis) | Most of the reported transitional care interventions reduced re-hospitalizations, with the exception of models led by GPs or primary care nurses |
| **Backman et al. (2020)** | To analyze evidence on the effectiveness of person- and family-centered care (PFCC) transition interventions on quality of care and the experience of patients | 28/6,127  Up to 11/2016  RCTs (n=28) | 25–1,413 patients/ per study    Adults (≥18 years) | - Population: Adults (≥18 years) - Interventions: PFCC transition interventions from hospital to home with at least one element from each component of the PFCC framework - Comparator: Usual care - Outcomes: Condition-specific knowledge, self-care behaviors, functional status, adverse events, quality of life, medication adherence, follow-up adherence, and satisfaction - Study types: RCTs | - Non-randomized experimental studies, qualitative studies, editorials, commentaries or study protocols - PFCC transition interventions from emergency departments to home - Studies related to obstetrics, gynecology, psychiatric or mental health | - | Interventions varied in the extent of PFCC focus and the comprehensiveness of care transition. Multifaceted PFCC interventions had mixed effects on patient-oriented outcomes |
| **Becker et al. (2021)** | To examine the effect of communication interventions delivered at hospital discharge on patient-relevant outcomes | 60/15,578  Up to 02/2021  RCTs (n=60) | 16,070 patients    Medical patients | - Population: Medical patients - Interventions: Communication interventions delivered shortly before or at hospital discharge - Comparator: Usual care/ standard discharge care - Outcomes: readmission, medical adherence, mortality, patient satisfaction, medical knowledge, or ED reattendance - Study types: RCTs | - Studies conducted in surgical wards, psychiatric hospitals, or outpatient settings - Interventions continued after discharge (e.g., ongoing teaching sessions) | **Method:**  (3) Statistical testing of subgroup differences  **Subgroup types:**   - Intervention types/ components - Medical conditions - Age - Gender - Country/region - Setting - Study quality/ RoB | Communication interventions at discharge were significantly associated with fewer hospital readmissions, greater treatment adherence, and higher patient satisfaction |
| **Bonetti et al. (2020)** | To evaluate the impact of pharmacist-led discharge counseling on hospital readmissions and ED visits through a systematic review and meta-analysis | 21/2,660  Up to 2016 (updated 03/2021)  RCTs (n=21) | 7,244 patients  ≥60 years (81%), 38% with chronic conditions | - Population: Patients with any clinical condition, gender, or age - Intervention: Pharmacist-led discharge counseling - Comparator: - Comparator: Usual care (defined as patients who received the usual treatment in regular practice) - Outcomes: Hospital readmissions and ED visits - Study types: RCTs | - Discharge counseling delivered by non-pharmacists or multidisciplinary teams - Comparisons between pharmacist-led discharge counseling and an intervention led by another healthcare professional - Control group also receiving discharge counseling from a pharmacist - Study designs other than RCTs - Studies that reported other pharmacist interventions, but not discharge counseling, - Counseling not performed at discharge, - Studies not reporting the outcomes of interest | **Method:**  (2) Narrative or visual comparison of subgroup effects  **Subgroup types:**   - Intervention intensity | Meta-analysis showed a significant reduction in overall hospital readmissions and ED visits for pharmacist-led discharge counseling compared to usual care. However, conclusions were limited by the small number of included studies, substantial heterogeneity, and wide prediction intervals which prevented drawing further conclusions |
| **Chartrand et al. (2023)** | To examine evidence on patient- and family-centered (PFC) care transition interventions and evaluate their effectiveness in reducing hospital readmissions and ED visits among adults after discharge | 50/10,021  Up to 03/2021  RCTs (n=50) | 13,985 patients    Patients ≥18 years | - Population: Patients ≥18 years - Intervention: PFC care transition interventions from hospital to home provided during or after hospitalization include at least one element from each component of the PFCC framework and focus on the patient and/or their family or caregiver - Comparator: Usual care, simplified intervention, or no intervention - Outcomes: Hospital readmissions and ED visits - Study types: RCTs, cluster RCTs, or pilot RCTs | - Studies conducted among adults discharged to acute care settings or specialty nursing facilities - Transitions from ED to home - Studies in pediatrics, obstetrics, gynecology, psychiatry, or mental health - Non-randomized experimental and qualitative studies | **Method:**  (2) Narrative or visual comparison of subgroup effects  **Subgroup types:**   - Intervention intensity | PFC care transition interventions appear to significantly reduce the risk of unplanned hospital readmissions but have minimal impact on ED visit rates |
| **Cheema et al. (2018)** | To assess the impact of pharmacist-led medication reconciliation on healthcare outcomes | 18/724  Up to 2016  RCTs (n=18) | 6,038 patients  Adults (≥18 years) | - Population: Adults (≥18 years) - Interventions: Pharmacist-led medicine reconciliation on certain outcomes in hospital settings - Comparator: Standard or routine care - Outcomes: medication discrepancies, potential/ preventable ADEs, healthcare utilization post-discharge - Study types: RCTs | - Study protocols, non-RCTs, conference abstracts - Non-hospital settings - Interventions not delivered by pharmacists - Studies with different study outcomes than those defined in the inclusion criteria | - | Pharmacist-led interventions substantially decreased medication discrepancies compared with standard care. No meaningful reductions were found in potential or preventable adverse drug events or healthcare utilization |
| **Daliri et al. (2021)** | To examine the effects of medication-­related interventions delivered both during hospitalization and after discharge on hospital readmissions, medication-­related problems, medication adherence and mortality | 15/11,741 (15 records related to 14 studies)  Up to 06/2019  RCTs (n=9) Before–after study (n=2) Controlled clinical trial (n=2) Matched case-control study (n=1) | 8,182 patients  Adults aged 48–77 years (usual care) and 47–75 (intervention group) | - Population: Adults (aged ≥18 years) hospitalized for at least 24 hours - Interventions: Medication-related interventions delivered by any healthcare professional both during hospitalization and within 1 month after discharge - Comparator: Usual care - Outcomes: Readmissions, medication-related problems (MRPs), medication adherence or nonadherence, mortality - Study types: Prospective studies | - Studies examining only a specific medication group - Literature reviews or non-peer-reviewed publications - Patients with psychiatric disorders, cognitive impairment, or those discharged to another institution (e.g., nursing homes) - Studies with unextractable data | **Method:**  (2) Narrative or visual comparison of subgroup effects  **Subgroup types:**   - Intervention intensity - Professional group - Medical conditions *(planned, but too few studies)* - Study quality/RoB *(planned, but too few studies)* | Medication-related interventions delivered both during hospitalization and post-discharge reduced 30-day readmissions compared with usual care. Medication adherence and MRPs may be improved. Effects on mortality were unclear. |
| **Fønss Rasmussen et al. (2021)** | To evaluate the impact of transitional care interventions that include both pre- and post-discharge components on readmissions among older medical patients | 11/1,951  01/2008–09/2019  RCTs (n=5) NRCTs (n=4) Cohort analytic (n=2) | ~ 24,500 patients  Mean age = 78 years (range 74.9–83.6) (intervention group)  Mean age = 79 years (range 75.2–84.5) (control group) | - Population: Medical patients (≥65 years or mean age ≥75) years discharged from a general medical ward or ED - Intervention: in the transitional phase between hospital and home which examined the impact of the intervention on readmission rates. The interventions had to include both predischarge and postdischarge components. - Comparator: Usual care defined as standard care and treatment - Outcome: Unplanned hospital readmission | - Patients who were non-medical (e.g., surgical), had only one medical diagnosis, or were diagnosed with psychiatric disorders - Interventions including only pre- or post-discharge components - Study designs such as reviews, case reports or case studies without comparison groups | **Method:**  (2) Narrative or visual comparison of subgroup effects  **Subgroup types:**   - Intervention intensity - Patient complexity - Country/region - Sample size - Study quality/RoB | Most transitional care interventions had a positive impact on readmission rates among older medical patients, particularly within 30 days after hospital discharge. However, no specific intervention could be recommended based on the available evidence |
| **Gillespie et al. (2023)** | To determine the effect of discharge education interventions that included a surgical wound care component compared with standard education given to general surgery patients before, or up to 30 days after hospital discharge on clinical and patient-reported outcomes | 10/2,536  2010–2021  RCTs (n=8) Non-randomized studies (n=2) | n.a.    Adult surgical patients | - Population: Adult patients who underwent an elective or emergent laparoscopic or laparotomic surgery in specified surgical locations - Interventions: Discharge education, information or communication for managing surgical wound care - Comparator: Standard/usual discharge education - Study settings: Single or multisite studies in tertiary or quaternary hospitals (public or private) - Study types: RCTs or non-randomized studies of interventions | - Reconstructive, trauma, transplant, biopsy only, or bariatric surgery - Surgical wounds not primarily closed - Case studies, case controls, case series, cohort studies, cross-sectional studies, etc. - Patient discharge education provided preoperatively or delivered within a multi-component protocol - Studies not focusing on or including content about the discharge period of postoperative wound care/ management - Results could not be disaggregated for general surgery | - | Due to the uncertainty of the evidence base, the effect of discharge education on clinical and patient-reported outcomes in general surgery could not be determined |
| **Gonçalves-Bradley et al. (2022)** | To examine whether individualized hospital discharge planning improves health care quality by reducing delayed discharge and readmission, and by improving health status, and to assess the costs of implementation | 33/4,632  Up to 04/2021  Parallel randomized trials (n=33) | 12,242 patients    Medical diagnosis, average age range 60–84 years | - Population: Hospitalized patients (acute, rehabilitation, or community settings) irrespective of age, gender or condition - Intervention: Individualized discharge plan developed for a patient prior to them leaving hospital (for home or residential care) - Comparator: Standard care without individualized discharge planning - Study types: RCTs | - Studies lacking an assessment or implementation phase in discharge planning - Interventions in which discharge planning was a minor component of a broader intervention - Interventions focusing solely on post-discharge care provision | **Methods:**  (5) Results described but not compared (not considered in subgroup synthesis) | Individualized discharge planning probably reduces hospital length of stay and may slightly reduce unscheduled readmissions. It may also improve patient and provider satisfaction, but evidence regarding cost implications is uncertain |
| **Gwadry-Sridhar et al. (2004)** | To evaluate the effectiveness of multidisciplinary heart failure management programs in reducing hospital admission rates | 8/529  1966–2000  RCTs (n=8) | 1,049 patients  Patients with heart failure, mean age:  71.0–80.3 years | - Population: Adults (≥18 years) hospitalized for heart failure and enrolled during hospitalization or shortly before/after discharge - Intervention: Patient education - Comparator: Usual care - Outcomes: Unplanned readmission (primary outcome) - Study types: RCTs with intervention and control group design | n.a. | **Methods:**  (5) Results described but not compared (not considered in subgroup synthesis) | Heart-failure-specific multidisciplinary interventions significantly reduced hospital readmissions but did not affect mortality rates |
| **Hammad et al. (2017)** | To review evidence on the effects and cost/cost-effectiveness of complete pharmacy-led medicine reconciliation in hospital settings | 13/4,065  Up to 12/2015  Prospective Uncontrolled (n=5) RCTs (n=3) Before–after study (n=3) Prospective controlled (n=1) Quasi-controlled trial (n=1) | n.a.    Average age range: 55–93 years, equal male to female ratio | - Population: Patients receiving pharmacy-led medicine reconciliation during hospital inpatient settings, all admission types and ward specialties - Intervention: Defined as “complete MR”, i.e., reconciliation fully implemented from admission through discharge, including accurate transfer of information to next health provider - Comparator: Standard/usual care | - Studies evaluating non-pharmacy-led MR at only one end of patient care or transfer - Studies evaluating pharmacy-led MR using a qualitative approach - Studies evaluating enhanced interventions, including telephone helpline and post discharge follow-up calls | **Method:**  (2) Narrative or visual comparison of subgroup effects  **Subgroup types:**   - Intervention intensity | Due to the lack of evidence, conclusions could not be drawn regarding the effects and costs of complete pharmacy-led medicine reconciliation |
| **Hansen et al. (2011)** | To summarize interventions studied to reduce 30-day rehospitalization and describe the best published evidence for effectiveness of these interventions | 43/4,013  01/1975–01/2011  Quasi-experimental and cohort(n=20)  RCTs (n=16) Non-controlled before–after studies (n=7) | 34–700,000 patients per study  General medical adult population | - Population: General medical adult population - Interventions: Peri-discharge process interventions - Comparator: Non-intervention cohort - Outcomes: Relative 30-day readmission outcomes for intervention vs. non-intervention cohort - Study designs: Experimental or observational (prospective or retrospective) | - Pediatric, obstetric or psychiatric populations - Reviews, editorials or case reports - Disease-specific interventions not applicable to general inpatient populations (e.g., BNP testing in heart failure) - Studies without 30-day readmission end point | **Method:**  (2) Narrative or visual comparison of subgroup effects  **Subgroup types:**   - Intervention intensity - Intervention types/components | No single intervention implemented alone was regularly associated with reduced risk for 30-day rehospitalization. |
| **Hesselink et al. (2012)** | To describe interventions tested in RCTs that aimed to improve patient handovers from hospital to primary care, and to evaluate their overall effects | 36/1,162  01/1990–03/2011  RCTs (n=36) | 20–1,098 patients (intervention group);  14–1,107 Patient (control group)  No further characteristics specified | - Population: Patients and providers involved in hospital-to-primary care or hospital-to-home care transitions - Interventions: Explicitly targeting at least one component of handover process (before, during, or after physical transition of patient) within country borders - Comparator: Usual/standard care - Outcomes: Min. one outcome related to handover quality or safety, or patient status within three months after discharge - Study types: RCTs | - Studies involving psychiatric patients, patients <18 years, or pregnant women - Studies examining only healthcare costs or expenditures | **Method:**  (2) Narrative or visual comparison of subgroup effects  **Subgroup types:**   - Intervention intensity - Intervention types/components - Other (Alignment of intervention purpose and outcome measured) | Most interventions were multicomponent and had statistically significant benefits in one or more outcomes. However, no single intervention was consistently associated with positive effects on specific outcomes. Benefits were most often reported for hospital utilization, continuity of care, and post-discharge patient status. Due to the complexity of interventions and outcome measures, firm conclusions could not be drawn. |
| **Lambrinou et al. (2012)** | To investigate the effects of nurse-driven pre-discharge actions in disease management programs for heart failure patients on hospital readmissions and to explore characteristics associated with effectiveness | 19/12,809  Up to 12/2009  RCTs (n=19) | 70–1,023 patients per study  Heart failure patients | - Population: Heart failure patients - Interventions: Heart failure disease management programs that included nurse-directed discharge planning or any kind of nurse-directed pre-discharge care, in addition to the usual care - Comparator: Usual care - Outcomes: Readmission - Study types: RCTs | - Studies assessing additional conditions beyond heart failure - Technology-related interventions (e.g., telemedicine) or medication management (except for titration/ optimization) - Unpublished or pilot studies | **Method:**  (3) Statistical testing of subgroup differences  **Subgroup types:**   - Intervention intensity - Intervention types/ components - Country/region | Nurse-led pre-discharge interventions in heart failure management programs may reduce hospital readmissions. However, essential characteristics or components of successful heart failure management programs remain to be identified. |
| **Lee et al. (2022)** | To systematically review and synthesize evidence regarding the effects of transitional care on health outcomes among frail older adults discharged from hospital to home | 21/3,182  Up to 08/2021  RCTs (n=21) | 5,776 patients  Age range: 77.0–85.7 years | - Population: Frail older adults (≥ 65 years) discharged from hospital to home - Interventions: Healthcare services that provided continuity of care; Focus on hospital-based transitional care initiated during admission and continued after discharge. Comparator: - Comparator: Usual care - Outcomes: Impact of transitional care on all health outcomes - Study types: RCTs | - Interventions not of interest (e.g., initiated only after discharge or involving transition to long-term care) - Non-RCTs | - | The meta-analysis found no effect on mortality or quality of life but showed reduced 6-month readmission rates. Narrative synthesis indicated improvements in self-rated health, life satisfaction, and functional status. |
| **Leithaus et al. (2022)** | (1) To identify integrated components used in transitional care models from hospital to home for frail older patients and map them to the SELFIE framework (2) To list service-, patient- and provider-level outcomes  (3) To identify components of transitional care models that reduce readmissions and ED visits | 17/6,221  01/2000–06/2020  RCTs (n=7)  Quasi-experimental (n=4) Retrospective cohort studies (n=2) Retrospective analysis of a clinical demonstration (n=1) Retrospective case-control study (n=1) Matched case- control study (n=1) Before–after study (n=1) | 107–8,936 patients per study​​  Mean age: 75.1– 82.6 years (intervention group); 74.4– 82.8 years (control group) | - Population: Adults ≥ 65 years with a frailty profile (e.g., functional decline or ≥1 chronic conditions) - Interventions: Transitional care models focusing on transition from hospital to home and including both pre-discharge and post-discharge components - Comparator: Usual or control care - Outcomes: at least hospital readmission or ED visit rates | - Descriptive or qualitative studies - Interventions limited to the ED or non-hospitalized patients - Palliative care interventions - Transitions not involving hospital to home​​ | **Method:**  (1) Inductive analysis of successful interventions  **Subgroup types:**   - Intervention types/ components | Multicomponent transitional care models with both pre-discharge and intensive post-discharge follow-up components may reduce hospital readmissions and ED visits. Components such as shared decision-making and the involvement of an informal caregiver, as well as a small, tailored care team with a defined coordinator, can increase their effectiveness. However, heterogeneity and poor reporting limited generalizability |
| **Mabire et al. (2018)** | To determine the effectiveness of nursing discharge planning interventions involving at least one nurse on health-related outcomes including readmission, length of stay, functional limitations, symptom management, unmet needs and health-related quality of life in older inpatients discharged home | 13/1,217  2000–2015  RCTs (n=10) RCT pilot study (n=1) Pilot cohort study (n=1) Before-after study (n=1) | 3,964  Older inpatients  (median age: 77 years) | - Population: Older inpatients (≥65 years) discharged home from acute care or a post-acute care rehabilitation setting - Interventions: Discharge planning interventions delivered by at least one nurse and involving a multidisciplinary or interdisciplinary model of care - Comparator: Usual (standard) care - Outcomes: readmission, length of stay, healthcare utilization, functional status, unmet needs and care satisfaction - Study types: RCTs, nonrandomized controlled trials, quasi experimental, before-after, prospective/retrospective cohort, case–control and analytical cross-sectional studies | n.a. | **Method:**  (2) Narrative or visual comparison of subgroup effects  **Subgroup types:**   - Intervention types/ components - Professional group - Age - Country/region | Nurse-led discharge planning had no significant effect on quality of life or hospital readmissions overall, although readmissions were statistically significantly lower in studies based in the US. Length of stay was significantly increased by nurse discharge planning. |
| **Mekonnen et al. (a) (2016)** | To assess the effectiveness of pharmacist-led medication reconciliation programs on clinical outcomes at hospital transition | 17/2,610  Up to 12/2014  RCTs (n=8) Before–after (n= 6) Non-RCTs (n= 3) | 21,342    Primarily high-risk patients (e.g., elderly, polypharmacy, risk of medication-related events) | - Population: n.a. - Interventions: Pharmacist-led, initiated in hospital and targeting transitions to and from hospital - Comparator: Usual or standard care (i.e., medication reconciliation not undertaken or not pharmacist-led) - Outcomes: All-cause readmission, ED visits, composite rate of readmission and/or ED visits, mortality, hospital visits related to adverse drug events (ADEs), ≥30 days of follow-up - Study types: RCTs, quasi-experimental studies with a control group, before–after studies | - Other medication reconciliation practices (e.g., nurse-led) or practices as part of a multicomponent intervention (e.g., medication therapy management) - Case studies, systematic reviews, qualitative outcomes, and non-research articles, conference abstracts | **Method:**  (3) Statistical testing of subgroup differences  **Subgroup types:**   - Study design | Pharmacist-led medication reconciliation at hospital transitions reduced ADE-related revisits, all-cause readmissions and ED visits. Effects on mortality and composite all-cause readmissions/ED visits were inconclusive, although the majority of studies showed improvement |
| **Mekonnen et al. (b) (2016)** | To evaluate the impact of pharmacy-led medication reconciliation interventions on reducing medication discrepancies at hospital transitions, and categorize these according to the intervention target (single transition, multiple transitions) | 19/2,551  Up to 12/2014  RCTs (n=11) Before–after studies (n=5) Non-RCTs (n=3) | 15,525    Adult patients of various ages | - Population: Not explicitly defined - Interventions: Medication reconciliation interventions delivered in hospital primarily by pharmacy personnel - Comparator: Usual care - Outcomes: Medication discrepancies | - Interventions delivered by physicians or nurses - Studies assessing discrepancies in documentation (e.g., allergy records) - Non-English publications, duplicate references, conference abstracts, or irrelevant study types (e.g., case studies, systematic reviews, qualitative studies) | **Method:**  (3) Statistical testing of subgroup differences  **Subgroup types:**   - Professional group - Intervention timing - Intervention intensity - Study setting - Study design | Pharmacy-led medication reconciliation interventions reduced medication discrepancies, particularly when delivered at admission or discharge. Interventions targeting multiple transitions were less effective |
| **Meulenbroeks et al. (2021)** | To assess whether caregiver-inclusive transitional care programs provide better value care (defined by the quadruple aim) compared to routine care | 23/1,580  Up to 06/2019  RCTs (n=14) Quasi-experimental studies (n=9) | 16,657    Mean age: 77.8 years, 46.4% male, 83.7% diagnosed with general medical conditions; remainder with stroke-specific diagnoses | - Population: Adults (≥65 years) with moderate to severe geriatric syndrome traits - Interventions: Transitional care between acute and community settings, with routine caregiver involvement - Comparator: Routine care - Outcomes: Aligned with the quadruple aim | - Patients receiving palliative care | **Method:**  (2) Narrative or visual comparison of subgroup effects  **Subgroup types:**   - Intervention types/ components - Professional group - Intervention intensity - Study setting | Evidence was inconclusive on whether caregiver-inclusive transitional care programs provided better value than routine care. No conclusions could be draw on the influence of specific modes of caregiver integration, leadership, or intensity |
| **Oh et al. (2023)** | To evaluate the effectiveness of discharge education using the teach-back method on hospital readmission rates in patients with heart failure | 7/619  Up to 05/2022  RCTs (n=3) Before–after control studies (n=3) One-group before–after study (n=1) | 29–171 participants per study    ≥18 years | - Population: Patients with heart failure scheduled for discharge from inpatient settings   Interventions: Discharge education using the teach-back method   - Comparator: Usual care - Outcomes: Unplanned readmissions - Study types: RCTs and quasi-experimental designs | - Patients <18 years - Non-nurse-led programs - Studies with high risk of bias | - | Teach-back discharge education reduced overall readmission rates for heart failure patients, although its effects on one-month readmission were not statistically significant |
| **Park et al. (2023)** | To synthesize evidence regarding the effectiveness of transitional care services on healthcare utilization and health outcomes in patients with COPD discharged from hospital | 9/3,961  01/17–12/21  RCTs (n=9) | 2,002  Adult patients (≥18 years) with COPD | - Population: Adult patients (≥18 years) with COPD - Interventions: Multidisciplinary approach that involves ≥1 providers, is consistent with the Transitional Care Model, includes a face-to-face component, and is initiated during hospitalization and continued post-discharge - Comparator: Usual/ standard care - Outcomes: Related to health or healthcare utilization - Study types: RCTs or cluster RCTs | - Studies on multiple diseases without distinguishing COPD-specific effects - Intervention limited to in-hospital discharge management or post-discharge community management only; remote-delivery; or delivered by a single nurse only - Control group not receiving usual/standard care - Outcomes not related to health or healthcare utilization - Non-English language publications | **Method:**  (1) Inductive analysis of successful interventions  **Subgroup types:**   - Intervention types/ components | No statistically significant differences were found between the intervention and control groups in terms of COPD-related readmissions or ED visits. Although the relative risk of readmission was lower in the intervention group, this difference was not statistically significant. Similarly, respiratory-related quality of life tended to be better in the intervention group, though not significantly. However, physical capacity was improved in the intervention group |
| **Rennke et al. (2013)** | To assess the effectiveness of hospital-initiated transitional care interventions in reducing clinical adverse events, ED visits, and readmissions in general medical patients after discharge | 47/20,248  1990–2012  RCTs (n=28) Controlled clinical trials (n=19) | n.a.  Majority of studies (n=27) targeted older adult populations; definitions of “elderly” varied | - Population: Undifferentiated population of adult general medical patients - Interventions: Transitional care strategy initiated before hospital discharge - Comparator: Usual discharge care - Outcomes: Post-discharge adverse event rates, ED utilization, or readmissions - Study types: RCTs and non-randomized, controlled clinical trials | - Disease-specific populations - Interventions not initiated in hospital or not explicitly targeting care transitions - Transitions to another acute or subacute setting - Studies reporting cost outcomes only, unless paired with one of the main outcomes | - | Ten studies showed reduced readmissions or ED visits with bridging strategies involving dedicated transition providers, but overall, the strength of evidence was low. There was insufficient evidence to determine how to prevent post-discharge adverse events |
| **Richards and Coast (2003)** | To determine the effectiveness and costs of interventions intended to improve access to health and social care for older patients following discharge from acute hospitals | 23/135  Up to 2000  RCTs (n=23) | n.a.    ≥60 years | - Population: older people (≥60 years) of any level of frailty, whose expected location upon discharge was the patient’s home - Interventions: Those aiming to improve access to post-discharge care by using standardized methods (e.g., comprehensive geriatric assessment, discharge-planning protocols, comprehensive care plans) to identify patient needs for post-discharge health and social care - Comparator: Routine care - Outcomes (at least one): Utilization of services, mortality, patient perceptions of health, quality of life, social support, cognitive functioning and well-being, adequacy of services, and patient functional health and disability | Studies in which routine care (control group) included standardized assessment tools or specialized assessors acting on behalf of a multidisciplinary team | **Methods:**  (5) Results described but not compared (not considered in subgroup synthesis) | Some evidence suggests that services combining needs assessment, discharge planning and a method for facilitating implementation are more effective than those lacking an implementation component |
| **Rodakowski et al. (2017)** | To evaluate the effects of integrating informal caregivers into the discharge planning process for older adults on post-discharge cost and resource use, including readmissions and length of rehospitalizations | 15/10,715 (13 unique studies)  1990–04/2016  RCTs (n=15) | 4,361    Average age  ≥65 years, 56% female | - Population: Older adults (average age ≥65 years) - Interventions: Discharge planning interventions initiated in hospital or skilled nursing facilities that integrate an informal caregiver into at least one part the intervention - Comparator: Usual care - Outcomes: cost and resource use, including readmissions and length of rehospitalizations - Study types: RCTs | Discharges to a non-community setting | - | The majority of studies reported statistically significant reductions in time to readmission, duration of rehospitalization, and costs of post-discharge care when caregivers were integrated into discharge planning |
| **Skjøt-Arkil et al. (2018)** | To evaluate the impact of multifaceted pharmacist-led interventions in hospital settings on patient and healthcare outcomes | 28/11,896  01/2006–11/2016 (additional search in two of the four databases from 11/2016 to 03/2018)  RCTs (n=16) Quasi-experimental studies (n=12) | 18,113  Mean age 58–85 years | - Population: Hospitalized patients - Interventions: Patient-related multifaceted interventions delivered by a clinical pharmacist and/or pharmacy technician during the hospital stay. Had to include at least three of the following: medication reconciliation, medication review, education, discharge report/communication with primary care. Disease- or drug-specific interventions were included if the full medication regimen was considered - Comparator: Usual care - Study types: Controlled studies (randomized trials at patient-level, cluster-randomized trials, and quasi-experimental trials) | - Interventions performed only by pharmacy students - Interventions conducted exclusively post-discharge - Studies involving outpatients and ED patients not admitted to hospital - Conference abstracts | **Method:**  (2) Narrative or visual comparison of subgroup effects  **Subgroup types:**   - Sample size | Multifaceted pharmacist-led interventions delivered in a hospital setting may improve quality of medication use and reduce hospital visits and length of stay. No effect was seen on mortality, patient-reported outcome or cost-effectiveness |
| **Stamp et al. (2014)** | To examine the effects of nurse-led transitional care interventions on hospital readmissions, quality of life, and cost-effectiveness in heart failure patients and the healthcare system | 20/850  “No date range selected”  RCTs (n=15) Quasi-experimental (n=2) Retrospective observational study (n=1) Prospective observational study (n=1) Randomized pilot study (n=1) | 70–1,023 patients per study  Heart failure patients aged ≥18 years | - Population: Heart failure patients aged ≥18 years - Interventions: Discharge planning and follow-up both pre-discharge and post-discharge - Comparator: Usual care - Study types: Qualitative and quantitative research | - Studies scoring less than an average of 20 out of 48 points methodological quality scale | **Method:**  (1) Inductive analysis of successful interventions  **Subgroup types:**   - Intervention types/ components - Intervention intensity | Transitional care programs for heart failure patients can improve quality of life, reduce readmissions and lower overall care costs. The most effective interventions involved home visits alone or combined with telephone follow-up |
| **Tomlinson et al. (2020)** | To evaluate evidence for interventions intended to support successful transitions of care through enhanced medication continuity for older adults | 24/2,394  01/2003–09/2019  RCTs (n=24) | 17,664  Mean age  ≥65 years | - Population: Older adults (mean age ≥65 years) being prepared for discharge or recently discharged from hospital - Interventions: Supporting medication continuity at discharge or within one month post-discharge - Comparator: Usual care - Outcomes: Readmission, safe medication use, quality of life - Study types: RCTs or cluster RCTs | - Interventions not specifically targeting medication continuity | **Method:**  (4) Mixed analyses (statistical testing/ narrative or visual comparison)  **Subgroup types:**   - Intervention types/ components - Intervention intensity | Medication-related interventions bridging the care transition led to the largest reductions in hospital readmissions. Effective components included self-management, telephone follow-up and medication reconciliation |
| **Tyler et al. (2023)** | To evaluate the comparative effectiveness of transitional care interventions with different levels of complexity in reducing health care utilization and improving outcomes in patients transitioning from hospital to community | 126/13,694  Up to 08/2022  RCTs (n=126) | 97,408  Median age: 66 years (range 59–75) | - Population: Patients in hospitals (acute, rehabilitation, or community) - Intervention: Transitional care interventions with different levels of complexity - Comparator: Usual care - Outcomes: Readmission (30, 90, 180 days), ED visits, mortality, quality of life, patient satisfaction, medication adherence, length of stay, primary care and other outpatient visits, and intervention uptake - Study types: RCTs or cluster RCTs | - Interventions where transitional/discharge component was minor - Studies examining only post-discharge follow-up in community without a hospital-based component | **Method:**  (3) Statistical testing of subgroup differences  **Subgroup types:**   - Intervention types/ components - Professional group - Intervention timing - Intervention intensity - Medical conditions - Patient complexity - Age - Gender - Country/region - Study quality/RoB | Low- and medium-complexity transitional care interventions were associated with reductions in health care utilization during the transition from hospital to community |
| **Verhaegh et al. (2014)** | (1) To examine whether transitional care interventions reduce readmission rates in chronically ill patients (2) To assess variations in the effects by intervent. intensity, follow-up time, patient age, sample size, health system and publ. date (3) To identify components associated with reduced readmissions | 26/8,092 1980–2013  RCTs (n=26) | 7,932    Chronically ill adults or patients at risk of poor outcomes after discharge | - Population: Chronically ill adults or patients at risk of poor outcomes after discharge and their informal caregivers - Interventions initiated during hospitalization and continued ≥1 month after discharge - Comparator: Usual care - Outcomes: Readmission rates in the short (30 days or less), intermediate (31–180 days), and long terms (181–365 days) - Study types: RCTs | - Focused on rehabilitation after a hospitalization - Pediatric or psychiatric patients | **Method:**  (4) Mixed analyses (statistical testing/ narrative or visual comparison)  **Subgroup types:**   - Intervention types/ components - Intervention intensity - Medical conditions - Age - Sample size - Country/ region - Other (publication date) | Transitional care was effective in reducing intermediate- and long-term all-cause hospital readmissions. Only high-intensity interventions appeared to be effective in reducing short-term readmissions |
| **Villeneueve et al. (2021)** | To describe the impact of pharmacist-led interventions during transitions of care on the use of healthcare services in older adults | 17/1,527  1990–02/2019 (updated in 08/2019)  RCTs (n = 11) Non-RCTs (n=4) Quasi-experimental studies (n=2) | 41–2,083 patients per study  In over half the studies (n=9), median patient age was >80 years; most studies w/ preponderance of women; all studies involved patients with polypharmacy (defined as use of ≥5 medications) | - Population: Patients aged ≥65 years - Interventions: Pharmacist-led interventions during transitions of care from a general acute medicine ward to home - Comparator: Usual care - Outcomes: Clinical outcomes related to healthcare utilization - Study types: RCTs or controlled studies | - Not a general geriatric population (e.g., oncology, cardiac insufficiency, majority of nursing home patients) - Did not evaluate a transitions of care or involve pharmacists in a meaningful way - Other reasons (e.g., systematic reviews, pharmacoeconomic analysis, study protocols) | **Method:**  (1) Inductive analysis of successful interventions  **Subgroup types:**   - Professional group - Intervention timing - Intervention intensity | By diversifying their interventions at different moments throughout transition of care, pharmacists can reduce the use of healthcare services for older adults during transition of care |
| **Weeda et al. (2023)** | To evaluate the impact of pharmacist-driven transition-of-care interventions on post-hospital outcomes in patients with coronary artery disease (CAD) | 11/1,612  Up to 03/2020  Prospective studies (n=6) RCTs (n=4) Retrospective study (n=1) | 3,024  Adult patients with CAD aged 54–73 years; most male (50–98%) | - Population: Adult patients with CAD (acute coronary syndrome events, coronary lesions, or undergoing coronary revascularization procedures) treated at US hospitals - Interventions: Pharmacist-driven interventions targeting transition-of-care (defined as discharge and/or follow-up process from acute care to an outpatient setting or outside facility) - Comparator: Control group - Outcomes: All-cause and cardiovascular-related, hospital readmissions and emergency room visits, mortality, CAD secondary prevention measures and medication adherence | n.a. | - | Pharmacist-driven transition-of-care interventions generally led to more favorable outcomes related to reencounters, mortality, CAD secondary prevention, and medication adherence. However, most studies were underpowered to detect significant differences in outcomes |

***Notes:*** *ADE = adverse drug event; BNP = brain natriuretic peptide; CAD = coronary artery disease; CHF = congestive heart failure; COPD = chronic obstructive pulmonary disease; DMP = disease management program; DP = discharge planning; ED = emergency department; GP = general practitioner; HF = heart failure; LoS = length of stay; MA = meta-analysis; MR = medication reconciliation; MRA = medication review and assessment; n.a. = not available/applicable; NRCT = non-randomized controlled trial; PFC = patient- and family-centered; PFCC = patient- and family-centered care; PRISMA = Preferred Reporting Items for Systematic Reviews and Meta-Analyses; QoL = quality of life; RCT = randomized controlled trial; RoB = risk of bias; SR = systematic review; TCI = transitional care intervention; TCM = Transitional Care Model; ToC = transitions of care; TBM = teach-back method; US = United States*

*Information, descriptions, and wording taken from review authors*

# **S9: Included primary studiesS per review**

|  | **Number of primary studies per review** | 24 | 12 | 28 | 60 | 21 | 50 | 18 | 15 | 11 | 10 | 33 | 8 | 13 | 43 | 36 | 19 | 21 | 17 | 13 | 17 | 19 | 23 | 7 | 9 | 47 | 23 | 15 | 28 | 20 | 24 | 126 | 26 | 17 | 11 |  |
| --- | --- | --- | --- | --- | --- | --- | --- | --- | --- | --- | --- | --- | --- | --- | --- | --- | --- | --- | --- | --- | --- | --- | --- | --- | --- | --- | --- | --- | --- | --- | --- | --- | --- | --- | --- | --- |
| ***Author*** | ***Country*** | **Albert et al. (2016)** | **Allen et al. (2014)** | **Backman et al. (2020)** | **Becker et al. (2021)** | **Bonetti et al. (2020)** | **Chartrand et al. (2020)** | **Cheema et al. (2018)** | **Daliri et al. (2021)** | **Fønss Rasmussen et al. (2021)** | **Gillespie et al. (2023)** | **Gonçalves-Bradley et al. (2022)** | **Gwadry-Sridhar et al. (2004)** | **Hammad et al. (2017)** | **Hansen et al. (2011)** | **Hesselink et al. (2012)** | **Lambrinou et al. (2012)** | **Lee et al. (2022)** | **Leithaus et al. (2022)** | **Mabire et al. (2018)** | **Mekonnen et al. (a) (2016)** | **Mekonnen et al. (b) (2016)** | **Meulenbroeks et al. (2021)** | **Oh et al. (2023)** | **Park et al. (2023)** | **Rennke et al. (2013)** | **Richards and Coast (2003)** | **Rodakowski et al. (2017)** | **Skjøt-Arkil et al. (2018)** | **Stamp et al. (2014)** | **Tomlinson et al. (2020)** | **Tyler et al. (2023)** | **Verhaegh et al. (2014)** | **Villeneueve et al. (2021)** | **Weeda et al. (2023)** | *Number of occurrence* |
| Aag et al. (2014) | Norway |  |  |  |  |  |  | x |  |  |  |  |  |  |  |  |  |  |  |  |  |  |  |  |  |  |  |  |  |  |  |  |  |  |  | 1 |
| Abizanda et al. (2011) | Spain |  |  |  |  |  |  |  |  |  |  |  |  |  |  |  |  |  |  |  |  |  | x |  |  |  |  |  |  |  |  |  |  |  |  | 1 |
| Aboumatar et al. (2019) | USA |  |  |  |  |  | x |  |  |  |  |  |  |  |  |  |  |  |  |  |  |  |  |  | x |  |  |  |  |  |  |  |  |  |  | 2 |
| Adamuz et al. (2015) | Spain |  |  |  | x |  |  |  |  |  |  |  |  |  |  |  |  |  |  |  |  |  |  |  |  |  |  |  |  |  |  | x |  |  |  | 2 |
| Adler et al. (2009) | n.a. |  |  |  |  |  |  |  |  |  |  |  |  |  |  |  |  |  |  |  |  |  |  |  |  | x |  |  |  |  |  |  |  |  |  | 1 |
| Afilalo et al. (2007) | Canada |  |  |  |  |  |  |  |  |  |  |  |  |  |  | x |  |  |  |  |  |  |  |  |  |  |  |  |  |  |  |  |  |  |  | 1 |
| Ahmad et al. (2010) | Netherlands |  |  |  |  |  |  |  |  |  |  |  |  |  |  |  |  |  |  |  |  |  |  |  |  |  |  |  |  |  | x |  |  |  |  | 1 |
| Ahmed et al. (2004) | USA |  |  |  |  |  |  |  |  |  |  |  |  |  | x |  |  |  |  |  |  |  |  |  |  |  |  |  |  |  |  |  |  |  |  | 1 |
| Alassaad et al. (2014) | Sweden |  |  |  |  |  |  |  |  |  |  |  |  |  |  |  |  |  |  |  |  |  |  |  |  |  |  |  | x |  |  |  |  |  |  | 1 |
| Alex et al. (2016) | USA |  |  |  |  |  |  |  |  |  |  |  |  |  |  |  |  |  |  |  |  |  |  |  |  |  |  |  | x |  |  |  |  |  |  | 1 |
| Al-Ghamdi et al. (2012) | Saudi Arabia |  |  |  |  |  |  |  |  |  |  |  |  |  |  |  |  |  |  |  |  |  |  |  |  | x |  |  |  |  |  |  |  |  |  | 1 |
| Al-Hashar et al. (2018) | Oman |  |  |  | x | x | x |  |  |  |  |  |  |  |  |  |  |  |  |  |  |  |  |  |  |  |  |  |  |  |  |  |  |  |  | 3 |
| Al-Rashed et al. (2002) | UK |  |  |  |  | x |  |  |  |  |  |  |  |  |  |  |  |  |  |  |  |  |  |  |  | x |  |  |  |  |  |  |  | x |  | 3 |
| Altfeld et al. (2012) | USA |  |  |  |  |  | x |  |  |  |  |  |  |  |  |  |  |  |  |  |  |  |  |  |  |  |  |  |  |  |  |  |  |  |  | 1 |
| Altfeld et al. (2013) | USA | x |  | x |  |  |  |  |  |  |  |  |  |  |  |  |  |  |  |  |  |  |  |  |  |  |  |  |  |  |  |  |  |  |  | 2 |
| Amarasingham et al. (2013) | USA | x |  |  |  |  |  |  |  |  |  |  |  |  |  |  |  |  |  |  |  |  |  |  |  |  |  |  |  |  |  |  |  |  |  | 1 |
| Anderegg et al. (2014) | USA |  |  |  |  |  |  |  |  |  |  |  |  | x |  |  |  |  |  |  | x |  |  |  |  |  |  |  |  |  |  |  |  |  | x | 3 |
| Andersen et al. (2000) | Denmark |  |  |  |  |  | x |  |  |  |  |  |  |  |  |  |  |  |  |  |  |  |  |  |  |  |  |  |  |  |  |  |  |  |  | 1 |
| Anderson et al. (2005) | USA | x |  |  |  |  |  |  |  |  |  |  |  |  | x |  | x |  |  |  |  |  |  |  |  |  |  |  |  | x |  |  |  |  |  | 4 |
| Anttila et al. (2000) | Finland |  |  |  |  |  |  |  |  |  |  |  |  |  |  |  |  |  |  | x |  |  |  |  |  |  |  |  |  |  |  |  |  |  |  | 1 |
| Applegate et al. (1990) | USA |  |  |  |  |  |  |  |  |  |  |  |  |  |  |  |  |  |  |  |  |  |  |  |  |  | x |  |  |  |  |  |  |  |  | 1 |
| Applegate et al. (1991) | USA |  |  |  |  |  |  |  |  |  |  |  |  |  |  |  |  |  |  |  |  |  |  |  |  |  | x |  |  |  |  |  |  |  |  | 1 |
| Arbaje et al. (2010) | USA |  |  |  |  |  |  |  |  |  |  |  |  |  |  | x |  |  |  | x |  |  |  |  |  |  |  |  |  |  |  |  |  |  |  | 2 |
| Arendts et al. (2018) | Australia |  |  |  |  |  |  |  |  |  |  |  |  |  |  |  |  |  |  |  |  |  |  |  |  |  |  |  |  |  |  | x |  |  |  | 1 |
| Askim et al. (2003) | Norway |  |  |  |  |  |  |  |  |  |  |  |  |  |  |  |  |  |  | x |  |  |  |  |  |  |  |  |  |  |  |  |  |  |  | 1 |
| Athar et al. (2018) | USA |  |  |  | x |  |  |  |  |  |  |  |  |  |  |  |  |  |  |  |  |  |  |  |  |  |  |  |  |  |  |  |  |  |  | 1 |
| Atienza et al. (2004) | Spain |  |  |  |  |  |  |  |  |  |  |  |  |  |  |  | x |  |  |  |  |  |  |  |  |  |  |  |  | x |  |  | x |  |  | 3 |
| Auger et al. (2018) | USA |  |  |  |  |  |  |  |  |  |  |  |  |  |  |  |  |  |  |  |  |  |  |  |  |  |  |  |  |  |  | x |  |  |  | 1 |
| Avlund et al. (2002) | Denmark |  |  |  |  |  |  |  |  |  |  |  |  |  |  | x |  |  |  |  |  |  |  |  |  |  |  |  |  |  |  |  |  |  |  | 1 |
| Awoke et al. (2019) | USA |  |  |  |  |  |  |  |  |  |  |  |  |  |  |  |  |  |  |  |  |  |  | x |  |  |  |  |  |  |  |  |  |  |  | 1 |
| Azevedo et al. (2002) | Portugal |  |  |  |  |  |  |  |  |  |  |  |  |  | x |  |  |  |  |  |  |  |  |  |  |  |  |  |  |  |  |  |  |  |  | 1 |
| Baker et al. (1991) | UK |  |  |  | x |  |  |  |  |  |  |  |  |  |  |  |  |  |  |  |  |  |  |  |  |  |  |  |  |  |  |  |  |  |  | 1 |
| Balaban et al. (2008) | USA |  |  |  |  |  |  |  | x |  |  | x |  |  | x |  |  |  |  |  |  |  |  |  |  | x |  |  |  |  |  | x |  |  |  | 5 |
| Balaban et al. (2015) | USA | x |  |  |  |  | x |  |  |  |  |  |  |  |  |  |  |  |  |  |  |  |  |  |  |  |  |  |  |  |  |  |  |  |  | 2 |
| Barfar et al. (2017) | UK |  |  |  |  |  |  |  |  |  |  |  |  |  |  |  |  |  |  |  |  |  |  |  |  |  |  |  |  |  |  | x |  |  |  | 1 |
| Barker et al. (2020) | UK |  |  |  | x |  |  |  |  |  |  |  |  |  |  |  |  |  |  |  |  |  |  |  |  |  |  |  |  |  |  | x |  |  |  | 2 |
| Basger at al. (2015) | Australia |  |  |  | x |  |  |  |  |  |  |  |  |  |  |  |  |  |  |  |  |  |  |  |  |  |  |  | x |  | x |  |  |  |  | 3 |
| Bawazeer et al. (2021) | Saudi Arabia |  |  |  |  |  |  |  |  |  |  |  |  |  |  |  |  |  |  |  |  |  |  |  |  |  |  |  |  |  |  | x |  |  |  | 1 |
| Becerra-Camargo et al. (2013) | USA |  |  |  |  |  |  | x |  |  |  |  |  |  |  |  |  |  |  |  |  | x |  |  |  |  |  |  |  |  |  |  |  |  |  | 2 |
| Becerra-Camargo et al. (2015) | Colombia |  |  |  |  |  |  | x |  |  |  |  |  |  |  |  |  |  |  |  |  |  |  |  |  |  |  |  |  |  |  |  |  |  |  | 1 |
| Beckett et al. (2012) | USA |  |  |  |  |  |  |  |  |  |  |  |  |  |  |  |  |  |  |  |  | x |  |  |  |  |  |  |  |  |  |  |  |  |  | 1 |
| Bell et al. (2016) | USA |  |  |  |  | x |  |  | x |  |  |  |  |  |  |  |  |  |  |  |  |  |  |  |  |  |  |  |  |  |  | x |  |  |  | 3 |
| Ben Said et al. (1994) | France |  |  |  | x |  |  |  |  |  |  |  |  |  |  |  |  |  |  |  |  |  |  |  |  |  |  |  |  |  |  |  |  |  |  | 1 |
| Bergkvist et al. (2009) (a) | Sweden |  |  |  |  |  |  |  |  |  |  |  |  |  |  |  |  |  |  |  |  | x |  |  |  |  |  |  | x |  |  |  |  |  |  | 2 |
| Bergkvist et al. (2009) (b) | Sweden |  |  |  |  |  |  |  |  |  |  |  |  |  |  |  |  |  |  |  |  |  |  |  |  |  |  |  | x |  |  |  |  |  |  | 1 |
| Berglund et al. (2013) | Sweden |  |  |  |  |  |  |  |  |  |  |  |  |  |  |  |  | x |  |  |  |  |  |  |  |  |  |  |  |  |  |  |  |  |  | 1 |
| Berglund et al. (2015) | Sweden |  |  |  |  |  |  |  |  |  |  |  |  |  |  |  |  | x |  |  |  |  |  |  |  |  |  |  |  |  |  |  |  |  |  | 1 |
| Biese et al. (2018) | USA |  |  |  |  |  |  |  |  |  |  |  |  |  |  |  |  |  |  |  |  |  |  |  |  |  |  |  |  |  |  | x |  |  |  | 1 |
| Bikmoradi et al. (2019) | Iran |  |  |  |  |  |  |  |  |  |  |  |  |  |  |  |  |  |  |  |  |  |  |  | x |  |  |  |  |  |  |  |  |  |  | 1 |
| Biscaglia et al. (2017) | Italy |  |  |  | x |  |  |  |  |  |  |  |  |  |  |  |  |  |  |  |  |  |  |  |  |  |  |  |  |  |  |  |  |  |  | 1 |
| Bladh et al. (2011) | Sweden |  |  |  | x |  |  |  |  |  |  |  |  |  |  |  |  |  |  |  |  |  |  |  |  |  |  |  | x |  |  |  |  |  |  | 2 |
| Bloodworth et al. (2019) | USA |  |  |  |  |  |  |  |  |  |  |  |  |  |  |  |  |  |  |  |  |  |  |  |  |  |  |  |  |  |  | x |  |  |  | 1 |
| Bloom et al. (2019) | USA |  |  |  |  |  |  |  |  |  |  |  |  |  |  |  |  |  |  |  |  |  |  |  |  |  |  |  |  |  |  | x |  |  |  | 1 |
| Blue et al. (2001) | UK |  |  |  |  |  |  |  |  |  |  |  |  |  |  |  | x |  |  |  |  |  |  |  |  |  |  |  |  | x |  |  |  |  |  | 2 |
| Blum et al. (2014) | USA |  |  |  |  |  |  |  |  |  |  |  |  |  |  |  |  |  |  |  |  |  |  |  |  |  |  |  |  |  |  | x |  |  |  | 1 |
| Bolas et al. (2004) | Ireland |  |  |  |  | x |  | x |  |  |  | x |  |  |  | x |  |  |  |  | x | x |  |  |  | x |  |  |  |  | x |  |  |  |  | 8 |
| Bonetti et al. (2018) | Brazil |  |  |  |  | x |  |  |  |  |  | x |  |  |  |  |  |  |  |  |  |  |  |  |  |  |  |  |  |  |  | x |  |  |  | 3 |
| Bonnet et al. (2013) | France |  |  |  |  |  |  |  |  |  |  |  |  |  |  |  |  |  |  |  |  |  |  |  |  |  |  | x |  |  |  |  |  |  |  | 1 |
| Bonnet Zamponi et al. (2013) | France |  |  |  |  |  |  |  |  |  |  |  |  |  |  |  |  |  |  |  |  |  |  |  |  |  |  |  |  |  |  | x |  |  |  | 1 |
| Bonsack et al. (2016) | Switzerland |  |  |  |  |  |  |  |  |  |  |  |  |  |  |  |  |  |  |  |  |  |  |  |  |  |  |  |  |  |  | x |  |  |  | 1 |
| Borok et al. (1994) | USA |  |  |  |  |  |  |  |  |  |  |  |  |  |  |  |  |  |  |  |  |  |  |  |  |  | x |  |  |  |  |  |  |  |  | 1 |
| Bostrom et al. (1996) | USA |  |  | x |  |  | x |  |  |  |  |  |  |  | x |  |  |  |  |  |  |  |  |  |  |  |  |  |  |  |  |  |  |  |  | 3 |
| Boter et al. (2004) | Netherlands |  |  | x |  |  | x |  |  |  |  |  |  |  |  |  |  |  |  |  |  |  |  |  |  |  |  |  |  |  |  |  |  |  |  | 2 |
| Bouchard et al. (2021) | France |  |  |  |  |  |  |  |  |  |  |  |  |  |  |  |  |  |  |  |  |  |  |  |  |  |  |  |  |  |  | x |  |  |  | 1 |
| Boult et al. (2011) | USA | x |  |  |  |  |  |  |  |  |  |  |  |  |  |  |  |  |  |  |  |  |  |  |  |  |  |  |  |  |  |  |  |  |  | 1 |
| Boult et al. (2013) | USA | x |  |  |  |  |  |  |  |  |  |  |  |  |  |  |  |  |  |  |  |  |  |  |  |  |  |  |  |  |  |  |  |  |  | 1 |
| Boyd et al. (2009) | USA | x |  |  |  |  |  |  |  |  |  |  |  |  |  |  |  |  |  |  |  |  |  |  |  |  |  |  |  |  |  |  |  |  |  | 1 |
| Boyde et al. (2018) | Australia |  |  |  |  |  |  |  |  |  |  |  |  |  |  |  |  |  |  |  |  |  |  | x |  |  |  |  |  |  |  |  |  |  |  | 1 |
| Brand et al. (2004) | USA |  |  |  |  |  |  |  |  |  |  |  |  |  |  |  |  |  | x |  |  |  |  |  |  | x |  |  |  |  |  |  |  |  |  | 2 |
| Braun et al. (2009) | Israel |  |  |  |  |  |  |  |  |  |  |  |  |  | x |  |  |  |  |  |  |  |  |  |  |  |  |  |  |  |  |  |  |  |  | 1 |
| Breathett et al. (2018) | USA |  |  |  | x |  |  |  |  |  |  |  |  |  |  |  |  |  |  |  |  |  |  |  |  |  |  |  |  |  |  |  |  |  |  | 1 |
| Bronstein et al. (2015) | USA |  |  |  |  |  | x |  |  |  |  |  |  |  |  |  |  |  |  |  |  |  |  |  |  |  |  |  |  |  |  |  |  |  |  | 1 |
| Brookes et al. (2000) | Ireland |  |  |  |  |  |  |  |  |  |  |  |  | x |  |  |  |  |  |  |  |  |  |  |  |  |  |  |  |  |  |  |  |  |  | 1 |
| Brown et al. (2017) | USA |  |  |  |  |  |  |  |  |  |  |  |  |  |  |  |  |  |  |  |  |  |  | x |  |  |  |  |  |  |  |  |  |  |  | 1 |
| Brown and Caplan (1997) | Australia |  |  |  |  |  |  |  |  |  |  |  |  |  | x |  |  |  |  |  |  |  |  |  |  |  |  |  |  |  |  |  |  |  |  | 1 |
| Bruehwiler et al. (2019) | Switzerland |  |  |  |  |  |  |  |  |  |  |  |  |  |  |  |  |  |  |  |  |  |  |  |  |  |  |  |  |  |  | x |  |  |  | 1 |
| Budiman et al. (2016) | USA |  |  |  |  |  |  |  |  |  |  |  |  |  |  |  |  |  |  |  |  |  |  |  |  |  |  |  |  |  |  |  |  |  | x | 1 |
| Burnett et al. (2009) | UK |  |  |  |  |  |  |  |  |  |  |  |  |  |  |  |  |  |  |  |  |  |  |  |  |  |  |  | x |  |  |  |  |  |  | 1 |
| Burns et al. (1995) | Germany |  |  |  |  |  |  |  |  |  |  |  |  |  |  |  |  |  |  |  |  |  |  |  |  |  | x |  |  |  |  |  |  |  |  | 1 |
| Burns et al. (2000) | Germany |  |  |  |  |  |  |  |  |  |  |  |  |  |  |  |  |  |  |  |  |  |  |  |  |  | x |  |  |  |  |  |  |  |  | 1 |
| Burns et al. (2014) | USA |  |  |  |  |  | x |  |  |  |  |  |  |  |  |  |  |  |  |  |  |  |  |  |  |  |  |  |  |  |  |  |  |  |  | 1 |
| Buurman et al. (2010) | Netherlands |  |  |  |  |  |  |  |  |  |  |  |  |  |  |  |  |  |  |  |  |  |  |  |  |  |  |  |  |  | x |  |  |  |  | 1 |
| Buurman et al. (2016) | Netherlands |  |  |  |  |  |  |  |  | x |  |  |  |  |  |  |  |  | x |  |  |  |  |  |  |  |  |  |  |  |  |  |  |  |  | 2 |
| Cajanding et al. (2017) | Philippines |  |  | x |  |  |  |  |  |  |  | x |  |  |  |  |  |  |  |  |  |  |  |  |  |  |  |  |  |  |  |  |  |  |  | 2 |
| Calvert et al. (2012) | USA |  |  |  |  |  |  |  | x |  |  |  |  |  |  |  |  |  |  |  |  |  |  |  |  |  |  |  |  |  |  |  |  |  | x | 2 |
| Caplan et al. (2004) | Australia |  |  |  |  |  |  |  |  |  |  |  |  |  |  | x |  |  |  |  |  |  |  |  |  |  |  |  |  |  |  |  |  |  |  | 1 |
| Carrington et al. (2013) | Australia |  |  |  |  |  |  |  |  |  |  |  |  |  |  |  |  |  |  | x |  |  |  |  |  |  |  |  |  |  |  |  |  |  |  | 1 |
| Casas et al. (2006) | Spain/ Belgium |  |  |  |  |  |  |  |  |  |  |  |  |  |  |  |  |  |  |  |  |  |  |  |  |  |  |  |  |  | x |  | x |  |  | 2 |
| Castelli et al. (2017) | USA |  |  |  | x |  |  |  |  |  |  |  |  |  |  |  |  |  |  |  |  |  |  |  |  |  |  |  |  |  |  |  |  |  |  | 1 |
| Castro et al. (2003) | Netherlands |  |  |  |  |  |  |  |  |  |  |  |  |  |  |  |  |  |  |  |  |  |  |  |  |  |  |  |  |  |  |  | x |  |  | 1 |
| Chakravarthy et al. (2018) | USA |  |  |  | x |  |  |  |  |  |  |  |  |  |  |  |  |  |  |  |  |  |  |  |  |  |  |  |  |  |  |  |  |  |  | 1 |
| Chan et al. (2015) | USA |  |  | x |  |  |  |  |  |  |  |  |  |  |  |  |  |  |  |  |  |  |  |  |  |  |  |  |  |  | x |  |  |  |  | 2 |
| Chan et al. (2016) | Taiwan |  |  |  | x |  |  |  |  |  |  |  |  |  |  |  |  |  |  |  |  |  |  |  |  |  |  |  |  |  |  |  |  |  |  | 1 |
| Char et al. (2017) | Singapore |  |  |  |  |  |  |  |  |  |  |  |  |  |  |  |  |  |  |  |  |  |  |  |  |  |  |  |  |  | x |  |  |  |  | 1 |
| Chen et al. (2010) | Australia |  |  |  |  |  |  |  |  |  |  |  |  |  |  | x |  |  |  |  |  |  |  |  |  |  |  |  |  |  |  |  |  |  |  | 1 |
| Chen et al. (2018) | China |  |  |  |  |  |  |  |  |  |  |  |  |  |  |  |  |  |  |  |  |  |  |  |  |  |  |  |  |  |  | x |  |  |  | 1 |
| Chiu et al. (2018) | China |  |  |  |  |  |  |  |  |  |  |  |  |  |  |  |  |  |  |  |  |  |  |  |  |  |  |  |  |  |  |  |  | x |  | 1 |
| Chow and Wong et al. (2014) | Hong Kong |  |  |  |  |  |  |  |  | x |  |  |  |  |  |  |  |  |  |  |  |  |  |  |  |  |  |  |  |  |  |  |  |  |  | 1 |
| Chow et al. (2010) | China |  |  | x |  |  |  |  |  |  |  |  |  |  |  |  |  |  |  |  |  |  |  |  |  |  |  |  |  |  |  |  |  |  |  | 1 |
| Cline et al. (1998) | Sweden |  |  |  |  |  |  |  |  |  |  |  | x |  |  |  | x |  |  |  |  |  |  |  |  |  |  |  |  |  |  |  | x |  |  | 3 |
| Coleman et al. (2004) | USA | x |  |  |  |  |  |  |  |  |  |  |  |  | x |  |  |  | x |  |  |  |  |  |  | x |  |  |  |  |  |  |  |  |  | 4 |
| Coleman et al. (2006) | USA | x | x |  |  |  | x |  |  |  |  |  |  |  | x |  |  |  | x |  |  |  | x |  |  | x |  |  |  |  | x |  | x |  |  | 9 |
| Collinsworth et al. (2018) | USA |  |  |  |  |  | x |  |  |  |  |  |  |  |  |  |  |  |  |  |  |  |  |  | x |  |  |  |  |  |  |  |  |  |  | 2 |
| Cordasco et al. (2009) | USA |  |  |  | x |  |  |  |  |  |  |  |  |  |  |  |  |  |  |  |  |  |  |  |  |  |  |  |  |  |  |  |  |  |  | 1 |
| Coskun et al. (2022) | Turkey |  |  |  |  |  |  |  |  |  |  |  |  |  |  |  |  |  |  |  |  |  |  |  |  |  |  |  |  |  |  | x |  |  |  | 1 |
| Counsell et al. (2000) | USA |  |  |  |  |  |  |  |  |  |  |  |  |  |  |  |  |  |  | x |  |  |  |  |  |  |  |  |  |  |  |  |  |  |  | 1 |
| Courtney et al. (2009) | Australia |  |  | x |  |  | x |  |  | x |  |  |  |  |  |  |  | x | x | x |  |  |  |  |  | x |  |  |  |  |  |  | x |  |  | 8 |
| Cowan et al. (2006) | Iran |  |  |  |  |  |  |  |  |  |  |  |  |  |  |  |  |  |  |  |  |  |  |  |  | x |  |  |  |  |  |  |  |  |  | 1 |
| Creason et al. (2001) | USA |  |  |  |  |  |  |  |  |  |  |  |  |  | x |  |  |  |  |  |  |  |  |  |  |  |  |  |  |  |  |  |  |  |  | 1 |
| Crotty et al. (2004) | Australia |  |  |  |  |  |  |  |  |  |  |  |  |  |  | x |  |  |  |  |  |  |  |  |  |  |  |  |  |  |  |  |  |  |  | 1 |
| Cui et al. (2019) | Australia |  |  |  |  |  | x |  |  |  |  |  |  |  |  |  |  |  |  |  |  |  |  |  |  |  |  |  |  |  |  |  |  |  |  | 1 |
| Dai et al. (2003) | Taiwan |  |  |  |  |  |  |  |  |  |  |  |  |  | x |  |  |  |  |  |  |  |  |  |  |  |  |  |  |  |  |  |  |  |  | 1 |
| Daliri et al. (2019) | Netherlands |  |  |  |  |  |  |  | x |  |  |  |  |  |  |  |  |  |  |  |  |  |  |  |  |  |  |  |  |  |  |  |  |  |  | 1 |
| Daly et al. (2005) | USA |  |  |  |  |  |  |  |  |  |  |  |  |  |  |  |  |  |  |  |  |  |  |  |  |  |  |  |  |  |  |  | x |  |  | 1 |
| Danielsen et al. (2020) | Norway |  |  |  |  |  |  |  |  |  |  |  |  |  |  |  |  |  |  |  |  |  |  |  |  |  |  |  |  |  |  | x |  |  |  | 1 |
| Davies et al. (2001) | UK |  |  |  | x |  |  |  |  |  |  |  |  |  |  |  |  |  |  |  |  |  |  |  |  |  |  |  |  |  |  |  |  |  |  | 1 |
| Davis et al. (2012) | USA |  |  | x |  |  | x |  |  |  |  |  |  |  |  |  |  |  |  |  |  |  |  |  |  |  |  |  |  |  |  |  |  |  |  | 2 |
| Dawes et al. (2007) | USA |  |  |  |  |  |  |  |  |  |  |  |  |  |  |  |  |  |  |  |  |  |  |  |  |  |  |  |  |  |  | x |  |  |  | 1 |
| Dawson et al. (2021) | Scotland |  |  |  |  |  |  |  |  |  |  |  |  |  |  |  |  |  |  |  |  |  |  |  |  |  |  |  |  |  |  | x |  |  |  | 1 |
| DeBusk et al. (2004) | USA |  |  |  |  |  |  |  |  |  |  |  |  |  |  |  |  |  |  |  |  |  |  |  |  |  |  |  |  | x |  |  |  |  |  | 1 |
| Dedhia et al. (2009) | USA | x |  |  |  |  |  |  |  |  |  |  |  |  | x |  |  |  |  | x |  |  |  |  |  |  |  |  |  |  |  |  |  | x |  | 4 |
| Del Sindaco et al. (2007) | Italy |  |  |  |  |  |  |  |  |  |  |  |  |  |  |  | x | x |  |  |  |  |  |  |  |  |  |  |  |  |  |  |  |  |  | 2 |
| Dellasega et al. (2000) | USA |  |  |  |  |  |  |  |  |  |  |  |  |  |  |  |  |  |  |  |  |  |  |  |  | x |  |  |  |  |  |  |  |  |  | 1 |
| Dempsey et al. (2018) | USA |  |  |  |  |  |  |  |  |  |  |  |  |  |  |  |  |  |  |  |  |  |  |  |  |  |  |  |  |  |  |  |  |  | x | 1 |
| Devore et al. (2021) | USA |  |  |  |  |  |  |  |  |  |  |  |  |  |  |  |  |  |  |  |  |  |  |  |  |  |  |  |  |  |  | x |  |  |  | 1 |
| Dhalla et al. (2014) | Canada |  |  |  |  |  |  |  |  |  |  |  |  |  |  |  |  |  |  |  |  |  |  |  |  |  |  |  |  |  |  | x |  |  |  | 1 |
| Dinh et al. (2019) | Vietnam |  |  |  |  |  |  |  |  |  |  |  |  |  |  |  |  |  |  |  |  |  |  | x |  |  |  |  |  |  |  |  |  |  |  | 1 |
| Doyle et al. (2020) | Australia |  |  |  | x |  |  |  |  |  |  |  |  |  |  |  |  |  |  |  |  |  |  |  |  |  |  |  |  |  |  |  |  |  |  | 1 |
| Dudas et al. (2001) | USA |  |  |  |  |  |  |  |  |  |  |  |  |  | x |  |  |  |  |  |  |  |  |  |  |  |  |  |  |  |  |  |  |  |  | 1 |
| Dunn et al. (1994) | UK |  |  |  |  |  |  |  |  |  |  |  |  |  | x |  |  |  |  |  |  |  |  |  |  |  |  |  |  |  |  |  |  |  |  | 1 |
| Ebrahimi et al. (2017) | Iran |  |  |  |  |  |  |  |  |  |  |  |  |  |  |  |  | x |  |  |  |  |  |  |  |  |  |  |  |  |  |  |  |  |  | 1 |
| Ebrahimi et al. (2021) | Iran |  |  |  | x |  |  |  |  |  |  |  |  |  |  |  |  |  |  |  |  |  |  |  |  |  |  |  |  |  |  |  |  |  |  | 1 |
| Edey et al. (2018) | Canada |  |  |  |  |  |  |  |  |  |  |  |  |  |  |  |  |  |  |  |  |  |  |  |  |  |  |  |  |  |  | x |  |  |  | 1 |
| Edman et al. (2013) | Sweden |  |  |  |  |  |  |  |  |  |  |  |  |  |  |  |  | x |  |  |  |  |  |  |  |  |  |  |  |  |  |  |  |  |  | 1 |
| Eggink et al. (2010) | Netherlands |  |  |  |  |  |  | x |  |  |  | x |  |  |  | x |  |  |  |  |  | x |  |  |  |  |  |  | x |  |  |  |  |  |  | 5 |
| Einstadter et al. (1996) | USA |  |  |  |  |  |  |  |  |  |  |  |  |  | x |  |  |  |  |  |  |  |  |  |  | x |  |  |  |  |  |  |  |  |  | 2 |
| Eisenhower (2014) | USA |  |  |  |  |  |  |  |  |  |  |  |  |  |  |  |  |  |  |  | x |  |  |  |  |  |  |  |  |  |  |  |  |  |  | 1 |
| Ekelund et al. (2015) | Sweden |  |  |  |  |  |  |  |  |  |  |  |  |  |  |  |  | x |  |  |  |  |  |  |  |  |  |  |  |  |  |  |  |  |  | 1 |
| Eklund et al. (2013) | Sweden |  |  |  |  |  |  |  |  |  |  |  |  |  |  |  |  | x |  |  |  |  |  |  |  |  |  |  |  |  |  |  |  |  |  | 1 |
| Ekman et al. (1998) | Sweden |  |  |  |  |  |  |  |  |  |  |  | x |  |  |  |  |  |  |  |  |  |  |  |  |  |  |  |  |  |  |  |  |  |  | 1 |
| Englander et al. (2014) | USA |  |  |  |  |  |  |  |  |  |  |  |  |  |  |  |  |  |  |  |  |  |  |  |  |  |  |  |  |  |  | x |  |  |  | 1 |
| Enguidanos et al. (2012) | USA |  | x |  |  |  |  |  |  |  |  |  |  |  |  |  |  |  |  |  |  |  |  |  |  |  |  |  |  |  |  |  |  |  |  | 1 |
| Esposito et al. (1995) | Finland |  |  |  | x |  |  |  |  |  |  |  |  |  |  |  |  |  |  |  |  |  |  |  |  |  |  |  |  |  |  |  |  |  |  | 1 |
| Evans and Hendricks (1993) | USA |  |  |  |  |  |  |  |  |  |  | x |  |  | x |  |  |  |  |  |  |  |  |  |  |  |  |  |  |  |  | x |  |  |  | 3 |
| Everink et al. (2018) | Netherlands |  |  |  |  |  |  |  |  |  |  |  |  |  |  |  |  |  |  |  |  |  | x |  |  |  |  |  |  |  |  |  |  |  |  | 1 |
| Eyler et al. (2016) | USA |  |  |  | x | x |  |  |  |  |  |  |  |  |  |  |  |  |  |  |  |  |  |  |  |  |  |  |  |  |  |  |  |  |  | 2 |
| Farley et al. (2014) | USA |  |  |  |  |  |  | x |  |  |  |  |  |  |  |  |  |  |  |  |  | x |  |  |  |  |  |  | x |  |  |  |  |  |  | 3 |
| Farris et al. (2014) | USA |  |  |  |  | x |  | x |  |  |  | x |  |  |  |  |  |  |  |  | x |  |  |  |  |  |  |  | x |  |  | x |  |  |  | 6 |
| Faulkner et al. (2000) | France |  |  |  |  |  |  |  |  |  |  |  |  |  |  |  |  |  |  |  |  |  |  |  |  |  |  |  |  |  |  |  |  |  | x | 1 |
| Finlayson et al. (2018) | Australia |  |  |  |  |  |  |  |  | x |  |  |  |  |  |  |  | x |  |  |  |  |  |  |  |  |  |  |  |  |  | x |  |  |  | 3 |
| Finn et al. (2011) | USA |  |  |  |  |  |  |  |  |  |  |  |  |  |  |  |  |  |  |  |  |  |  |  |  | x |  |  |  |  |  |  |  |  |  | 1 |
| Finn et al. (2020) | Australia |  |  |  |  |  |  |  |  |  |  |  |  |  |  |  |  |  |  |  |  |  |  |  |  |  |  |  |  |  |  | x |  |  |  | 1 |
| Fors et al. (2018) | Sweden |  |  |  |  |  | x |  |  |  |  |  |  |  |  |  |  |  |  |  |  |  |  |  |  |  |  |  |  |  |  |  |  |  |  | 1 |
| Forsmo et al. (2016) | Norway |  |  |  |  |  |  |  |  |  | x |  |  |  |  |  |  |  |  |  |  |  |  |  |  |  |  |  |  |  |  |  |  |  |  | 1 |
| Forster et al. (2005) | Canada |  |  |  |  |  |  |  |  |  |  |  |  |  | x |  |  |  |  | x |  |  |  |  |  | x |  |  |  |  |  |  | x |  |  | 4 |
| Forster et al. (2013) | UK |  |  |  |  |  |  |  |  |  |  |  |  |  |  |  |  |  |  |  |  |  | x |  |  |  |  | x |  |  |  |  |  |  |  | 2 |
| Fretwell et al. (1999) | USA |  |  |  |  |  |  |  |  |  |  |  |  |  |  |  |  |  |  |  |  |  |  |  |  |  | x |  |  |  |  |  |  |  |  | 1 |
| Fuenzalida et al. (2015) | Spain |  |  |  | x |  |  |  |  |  |  |  |  |  |  |  |  |  |  |  |  |  |  |  |  |  |  |  |  |  |  |  |  |  |  | 1 |
| Fuenzalida et al. (2017) | Spain |  |  |  | x |  |  |  |  |  |  |  |  |  |  |  |  |  |  |  |  |  |  |  |  |  |  |  |  |  |  |  |  |  |  | 1 |
| Gallagher et al. (2011) | UK |  |  |  |  |  |  |  |  |  |  |  |  |  |  |  |  |  |  |  |  |  |  |  |  | x |  |  |  |  |  |  |  |  |  | 1 |
| Gardella et al. (2012) | USA |  |  |  |  |  |  |  |  |  |  |  |  |  |  |  |  |  |  |  | x | x |  |  |  |  |  |  |  |  |  |  |  |  |  | 2 |
| Gardner et al. (2020) | USA |  |  |  |  |  |  |  |  |  |  |  |  |  |  |  |  |  |  |  |  |  |  |  |  |  |  |  |  |  |  | x |  |  |  | 1 |
| Gasbarro et al. (2015) | USA |  |  |  |  |  |  |  |  |  |  |  |  |  |  |  |  |  |  |  |  |  |  |  |  |  |  |  |  |  |  |  |  |  | x | 1 |
| Ghadiri-Vasfi et al. (2015) | Iran |  |  |  |  |  |  |  |  |  |  |  |  |  |  |  |  |  |  |  |  |  |  |  |  |  |  |  |  |  |  | x |  |  |  | 1 |
| Gilbert et al. (2021) | France |  |  |  |  |  |  |  |  |  |  |  |  |  |  |  |  |  |  |  |  |  |  |  |  |  |  |  |  |  |  | x |  |  |  | 1 |
| Gillard et al. (2022) | UK |  |  |  |  |  |  |  |  |  |  |  |  |  |  |  |  |  |  |  |  |  |  |  |  |  |  |  |  |  |  | x |  |  |  | 1 |
| Gillespie et al. (2009) | Sweden |  |  |  |  |  |  | x |  |  |  |  |  |  |  |  |  |  |  |  | x |  |  |  |  | x |  |  | x |  | x | x |  | x |  | 7 |
| Gillespie et al. (2013) | Sweden |  |  |  |  |  |  |  |  |  |  |  |  |  |  |  |  |  |  |  |  |  |  |  |  |  |  |  | x |  |  |  |  |  |  | 1 |
| Gillespie et al. (2019) | Sweden |  |  |  |  |  |  |  |  |  |  | x |  |  |  |  |  |  |  |  |  |  |  |  |  |  |  |  |  |  |  |  |  |  |  | 1 |
| Giuse et al. (2012) | USA |  |  |  | x |  |  |  |  |  |  |  |  |  |  |  |  |  |  |  |  |  |  |  |  |  |  |  |  |  |  |  |  |  |  | 1 |
| Goldman et al. (2014) | USA |  |  |  |  |  |  |  | x |  |  | x |  |  |  |  |  |  |  |  |  |  |  |  |  |  |  |  |  |  |  | x |  |  |  | 3 |
| Goncalves et al. (2016) | Brazil |  |  | x |  |  |  |  |  |  |  |  |  |  |  |  |  |  |  |  |  |  |  |  |  |  |  |  |  |  |  |  |  |  |  | 1 |
| Gorman et al. (2018) | USA |  |  |  |  |  |  |  |  |  |  |  |  |  |  |  |  |  |  |  |  |  |  |  |  |  |  |  |  |  |  |  |  |  | x | 1 |
| Gow et al. (1999) | New Zealand |  |  |  |  |  |  |  |  |  |  |  |  |  | x |  |  |  |  |  |  |  |  |  |  | x |  |  |  |  |  |  |  |  |  | 2 |
| Graabaek et al. (2018) | Denmark |  |  |  |  | x |  |  |  |  |  |  |  |  |  |  |  |  |  |  |  |  |  |  |  |  |  |  |  |  |  | x |  |  |  | 2 |
| Graabaek et al. (2019) | Denmark |  |  |  | x |  |  |  |  |  |  |  |  |  |  |  |  |  |  |  |  |  |  |  |  |  |  |  |  |  | x |  |  | x |  | 3 |
| Grafft et al. (2010) | USA |  |  |  |  |  |  |  |  |  |  |  |  |  | x |  |  |  |  |  |  |  |  |  |  |  |  |  |  |  |  |  |  |  |  | 1 |
| Grahn et al. (2019) | USA |  |  |  |  |  |  |  |  |  | x |  |  |  |  |  |  |  |  |  |  |  |  |  |  |  |  |  |  |  |  |  |  |  |  | 1 |
| Granados-Santiago et al. (2020) | Spain |  |  |  |  |  |  |  |  |  |  |  |  |  |  |  |  |  |  |  |  |  |  |  | x |  |  |  |  |  |  |  |  |  |  | 1 |
| Gräsel et al. (2005) | Germany |  |  |  |  |  |  |  |  |  |  |  |  |  |  |  |  |  |  |  |  |  | x |  |  |  |  |  |  |  |  |  |  |  |  | 1 |
| Graumlich et al. (2009) (a) | USA |  |  |  |  |  |  |  |  |  |  |  |  |  |  | x |  |  |  |  |  |  |  |  |  |  |  |  |  |  |  |  |  |  |  | 1 |
| Graumlich et al. (2009) (b) | USA |  |  |  |  |  |  |  |  |  |  |  |  |  |  | x |  |  |  |  |  |  |  |  |  | x |  |  |  |  |  |  |  |  |  | 2 |
| Gregersen et al. (2012) | Denmark |  |  |  |  |  |  |  |  |  |  |  |  |  |  |  |  |  | x |  |  |  |  |  |  |  |  |  |  |  |  |  |  |  |  | 1 |
| Griffey et al. (2015) | USA |  |  |  | x |  |  |  |  |  |  |  |  |  |  |  |  |  |  |  |  |  |  |  |  |  |  |  |  |  |  |  |  |  |  | 1 |
| Grimes et al. (2014) | Ireland |  |  |  |  |  |  |  |  |  |  |  |  |  |  |  |  |  |  |  |  | x |  |  |  |  |  |  |  |  |  |  |  |  |  | 1 |
| Guihan et al. (2014) | USA |  |  | x |  |  |  |  |  |  |  |  |  |  |  |  |  |  |  |  |  |  |  |  |  |  |  |  |  |  |  |  |  |  |  | 1 |
| Gurwitz et al. (2014) | USA |  |  |  |  |  |  |  |  |  |  |  |  |  |  |  |  |  |  |  |  |  |  |  |  |  |  |  |  |  | x | x |  |  |  | 2 |
| Gustafsson et al. (2017) | Sweden |  |  |  |  |  |  |  |  |  |  |  |  |  |  |  |  |  |  |  |  |  |  |  |  |  |  |  |  |  |  |  |  | x |  | 1 |
| Gwadry-Sridhar et al. (2001) | Canada |  |  |  |  |  |  |  |  |  |  |  | x |  |  |  |  |  |  |  |  |  |  |  |  |  |  |  |  |  |  |  |  |  |  | 1 |
| Gwadry-Sridhar et al. (2005) | UK |  |  |  | x |  |  |  |  |  |  |  |  |  |  |  |  |  |  |  |  |  |  |  |  |  |  |  |  |  |  |  |  |  |  | 1 |
| Haag et al. (2016) | USA |  |  |  |  |  |  |  |  |  |  |  |  |  |  |  |  |  |  |  |  |  |  |  |  |  |  |  |  |  | x |  |  |  |  | 1 |
| Habib et al. (2021) | Canada |  |  |  |  |  |  |  |  |  |  |  |  |  |  |  |  |  |  |  |  |  |  |  |  |  |  |  |  |  |  | x |  |  |  | 1 |
| Hanrahan et al. (2014) | USA |  |  |  |  |  |  |  |  |  |  |  |  |  |  |  |  |  |  |  |  |  |  |  |  |  |  |  |  |  |  | x |  |  |  | 1 |
| Hansen et al. (1995) | Denmark |  | x |  |  |  |  |  |  |  |  |  |  |  |  |  |  | x |  |  |  |  |  |  |  |  |  |  |  |  |  |  |  |  |  | 2 |
| Hanssen et al. (2007) | Norway |  |  | x |  |  |  |  |  |  |  |  |  |  |  |  |  |  |  |  |  |  |  |  |  |  |  |  |  |  |  |  |  |  |  | 1 |
| Hanssen et al. (2009) | Norway |  |  |  |  |  | x |  |  |  |  |  |  |  |  |  |  |  |  |  |  |  |  |  |  |  |  |  |  |  |  |  |  |  |  | 1 |
| Harrison et al. (2002) | Canada | x |  | x |  |  | x |  |  |  |  | x |  |  |  | x | x |  |  |  |  |  |  |  |  |  |  |  |  | x |  | x |  |  |  | 8 |
| Harrison et al. (2011) | USA |  |  |  |  |  |  |  |  |  |  |  |  |  | x |  |  |  |  |  |  |  |  |  |  |  |  |  |  |  |  |  |  |  |  | 1 |
| Harvey et al. (2014) | Australia |  |  |  |  |  |  |  |  |  |  |  |  |  |  |  |  |  |  |  |  |  |  |  |  |  |  |  |  |  |  | x |  |  |  | 1 |
| Hawes et al. (2014) | USA |  |  |  |  |  |  | x |  |  |  |  |  |  |  |  |  |  |  |  | x | x |  |  |  |  |  |  |  |  |  |  |  |  |  | 3 |
| Hayes et al. (1998) | USA |  |  |  | x |  |  |  |  |  |  |  |  |  |  |  |  |  |  |  |  |  |  |  |  |  |  |  |  |  |  |  |  |  |  | 1 |
| Heaton et al. (2019) | USA |  |  |  |  |  |  |  |  |  |  |  |  |  |  |  |  |  |  |  |  |  |  |  |  |  |  |  |  |  |  | x |  |  |  | 1 |
| Hebel et al. (2014) | Poland |  |  |  |  |  |  |  |  |  |  |  |  |  |  |  |  |  |  |  |  |  | x |  |  |  |  |  |  |  |  |  |  |  |  | 1 |
| Hegelund et al. (2019) | Denmark |  |  |  |  |  |  |  |  |  |  |  |  |  |  |  |  |  |  |  |  |  |  |  |  |  |  |  |  |  |  | x |  |  |  | 1 |
| Hegelund et al. (2020) | Denmark |  |  |  |  |  |  |  |  |  |  |  |  |  |  |  |  |  |  |  |  |  |  |  | x |  |  |  |  |  |  | x |  |  |  | 2 |
| Hellstrom et al. (2011) | Sweden |  |  |  |  |  |  |  |  |  |  |  |  | x |  |  |  |  |  |  | x |  |  |  |  |  |  |  | x |  |  |  |  | x |  | 4 |
| Hellstrom et al. (2012) | Sweden |  |  |  |  |  |  |  |  |  |  |  |  |  |  |  |  |  |  |  | x |  |  |  |  | x |  |  | x |  |  |  |  |  |  | 3 |
| Hendriksen et al. (1990) | Denmark |  |  |  |  |  |  |  |  |  |  | x |  |  |  |  |  |  |  |  |  |  |  |  |  |  |  |  |  |  |  |  |  |  |  | 1 |
| Hengartner et al. (2016) | Switzerland |  |  |  |  |  |  |  |  |  |  |  |  |  |  |  |  |  |  |  |  |  |  |  |  |  |  |  |  |  |  | x |  |  |  | 1 |
| Henschen et al. (2022) | USA |  |  |  |  |  | x |  |  |  |  |  |  |  |  |  |  |  |  |  |  |  |  |  |  |  |  |  |  |  |  |  |  |  |  | 1 |
| Hernandez et al. (2010) | USA |  |  |  |  |  |  |  |  |  |  |  |  |  | x |  |  |  |  |  |  |  |  |  |  |  |  |  |  |  |  |  |  |  |  | 1 |
| Hess et al. (2010) | USA |  |  |  |  |  |  |  |  |  |  |  |  |  | x |  |  |  |  |  |  |  |  |  |  |  |  |  |  |  |  |  |  |  |  | 1 |
| Hess et al. (2012) | USA |  |  |  | x |  |  |  |  |  |  |  |  |  |  |  |  |  |  |  |  |  |  |  |  |  |  |  |  |  |  |  |  |  |  | 1 |
| Hess et al. (2016) | USA |  |  |  | x |  |  |  |  |  |  |  |  |  |  |  |  |  |  |  |  |  |  |  |  |  |  |  |  |  |  |  |  |  |  | 1 |
| Hick et al. (2001) | UK |  |  |  |  |  |  |  |  |  |  |  |  | x |  |  |  |  |  |  |  |  |  |  |  |  |  |  |  |  |  |  |  |  |  | 1 |
| Hill et al. (2013) | Australia |  |  | x |  |  |  |  |  |  |  |  |  |  |  |  |  |  |  |  |  |  |  |  |  |  |  |  |  |  |  |  |  |  |  | 1 |
| Hill et al. (2016) | USA |  |  |  | x |  |  |  |  |  |  |  |  |  |  |  |  |  |  |  |  |  |  |  |  |  |  |  |  |  |  |  |  |  |  | 1 |
| Hilleman et al. (2001) | Israel |  |  |  |  |  |  |  |  |  |  |  |  |  |  |  |  |  |  |  |  |  |  |  |  |  |  |  |  |  |  |  |  |  | x | 1 |
| Hirsch et al. (2014) | Germany |  |  |  |  |  |  |  |  |  |  |  |  |  |  |  |  |  |  |  |  |  | x |  |  |  |  |  |  |  |  |  |  |  |  | 1 |
| Ho et al. (2013) | USA |  |  |  |  |  |  |  | x |  |  |  |  |  |  |  |  |  |  |  |  |  |  |  |  |  |  |  |  |  |  |  |  |  |  | 1 |
| Ho et al. (2014) | USA |  |  |  |  |  |  |  |  |  |  |  |  |  |  |  |  |  |  |  |  |  |  |  |  |  |  |  |  |  |  |  |  |  | x | 1 |
| Hockly et al. (2018) | UK |  |  |  |  |  |  |  |  |  |  |  |  |  |  |  |  |  |  |  |  |  |  |  |  |  |  |  |  |  | x |  |  |  |  | 1 |
| Hoffmann et al. (2015) | Australia |  |  | x |  |  |  |  |  |  |  |  |  |  |  |  |  |  |  |  |  |  |  |  |  |  |  |  |  |  |  |  |  |  |  | 1 |
| Hogan and Fox (1990) | Canada |  |  |  |  |  |  |  |  |  |  |  |  |  |  |  |  |  |  |  |  |  |  |  |  | x |  |  |  |  |  |  |  |  |  | 1 |
| Hogan et al. (1987) | Canada |  |  |  |  |  |  |  |  |  |  |  |  |  |  |  |  |  |  |  |  |  |  |  |  |  | x |  |  |  |  |  |  |  |  | 1 |
| Holland et al. (2005) | UK |  |  |  |  |  |  |  |  |  |  |  |  |  |  |  |  |  |  |  |  |  |  |  |  |  |  |  |  |  | x |  |  | x |  | 2 |
| Holmes-Rovner et al. (2008) | USA |  |  | x |  |  |  |  |  |  |  |  |  |  |  |  |  |  |  |  |  |  |  |  |  |  |  |  |  |  |  |  |  |  |  | 1 |
| Hoover et al. (2017) | USA |  |  |  |  |  |  |  | x |  |  |  |  |  |  |  |  |  |  |  |  |  |  |  |  |  |  |  |  |  |  |  |  |  |  | 1 |
| Hosein Abadi et al. (2020) | Iran |  |  |  |  |  |  |  |  |  |  |  |  |  |  |  |  |  |  |  |  |  |  |  |  |  |  |  |  |  |  | x |  |  |  | 1 |
| Houghton et al. (1996) | UK |  |  |  |  |  |  |  |  |  |  |  |  |  | x |  |  |  |  |  |  |  |  |  |  |  |  |  |  |  |  |  |  |  |  | 1 |
| Hu et al. (2020) | China |  |  |  |  |  | x |  |  |  |  |  |  |  |  |  |  |  |  |  |  |  |  |  |  |  |  |  |  |  |  | x |  |  |  | 2 |
| Huang et al. (2005) | Taiwan |  |  | x |  |  | x |  |  |  |  |  |  |  |  |  |  |  |  |  |  |  |  |  |  |  |  | x |  |  | x |  | x |  |  | 5 |
| Huckfeldt et al. (2019) | USA |  |  |  |  |  |  |  |  |  |  |  |  |  |  |  |  |  | x |  |  |  |  |  |  |  |  |  |  |  |  |  |  |  |  | 1 |
| Ihedioha et al. (2013) | UK |  |  |  |  |  |  |  |  |  | x |  |  |  |  |  |  |  |  |  |  |  |  |  |  |  |  |  |  |  |  |  |  |  |  | 1 |
| Indraratna et al. (2022) | Australia |  |  |  |  |  |  |  |  |  |  |  |  |  |  |  |  |  |  |  |  |  |  |  |  |  |  |  |  |  |  | x |  |  |  | 1 |
| Indredavik et al. (2000) | Norway |  |  |  |  |  |  |  |  |  |  |  |  |  |  | x |  |  |  |  |  |  | x |  |  |  |  |  |  |  |  |  |  |  |  | 2 |
| Israel et al. (2013) | USA |  |  |  |  |  |  |  |  |  |  |  |  | x |  |  |  |  |  |  |  |  |  |  |  |  |  |  | x |  |  |  |  |  |  | 2 |
| Jaarsma et al. (1999) | Netherlands |  |  |  |  |  |  |  |  |  |  |  | x |  | x |  | x |  |  |  |  |  |  |  |  |  |  |  |  | x |  |  |  |  |  | 4 |
| Jaarsma et al. (2000) | Netherlands |  |  | x |  |  |  |  |  |  |  |  |  |  |  |  |  |  |  |  |  |  |  |  |  |  |  |  |  |  |  |  |  |  |  | 1 |
| Jaarsma et al. (2008) | Netherlands |  |  |  |  |  |  |  |  |  |  |  |  |  |  |  | x |  |  |  |  |  |  |  |  |  |  |  |  | x |  |  |  |  |  | 2 |
| Jack et al. (2009) | USA | x |  |  |  |  |  |  | x |  |  | x |  |  | x | x |  |  |  |  |  |  |  |  |  | x |  |  |  |  |  | x |  |  |  | 7 |
| Jasinski et al. (2018) | USA |  |  |  | x |  |  |  |  |  |  |  |  |  |  |  |  |  |  |  |  |  |  |  |  |  |  |  |  |  |  |  |  |  |  | 1 |
| Jayaram et al. (2022) | Canada |  |  |  |  |  |  |  |  |  |  |  |  |  |  |  |  |  |  |  |  |  |  |  |  |  |  |  |  |  |  | x |  |  |  | 1 |
| Jennings et al. (2015) | USA |  |  |  |  |  |  |  |  |  |  |  |  |  |  |  |  |  |  |  |  |  |  |  |  |  |  |  |  |  |  | x |  |  |  | 1 |
| Jepma et al. (2021) | Netherlands |  |  |  |  |  |  |  |  |  |  |  |  |  |  |  |  | x |  |  |  |  |  |  |  |  |  |  |  |  |  |  |  |  |  | 1 |
| Jerant et al. (2003) | USA |  |  | x |  |  |  |  |  |  |  |  |  |  |  |  |  |  |  |  |  |  |  |  |  |  |  |  |  |  |  |  |  |  |  | 1 |
| Kalra et al. (2004) | UK |  |  |  |  |  |  |  |  |  |  |  |  |  |  |  |  |  |  |  |  |  | x |  |  |  |  |  |  |  |  |  |  |  |  | 1 |
| Kang et al. (2022) | Australia |  |  |  |  |  |  |  |  |  | x |  |  |  |  |  |  |  |  |  |  |  |  |  |  |  |  |  |  |  |  |  |  |  |  | 1 |
| Kangovi et al. (2014) | USA |  |  | x |  |  | x |  |  |  |  |  |  |  |  |  |  |  |  |  |  |  |  |  |  |  |  |  |  |  |  |  |  |  |  | 2 |
| Karaoui et al. (2020) | Lebanon |  |  |  |  |  |  |  |  |  |  |  |  |  |  |  |  |  |  |  |  |  |  |  |  |  |  |  |  |  |  | x |  |  |  | 1 |
| Karapinar-Carkit et al. (2012) | Netherlands |  |  |  |  |  |  |  |  |  |  |  |  | x |  |  |  |  |  |  |  |  |  |  |  |  |  |  |  |  |  |  |  |  |  | 1 |
| Karapinar-Çarkit et al. (2017) | Netherlands |  |  |  |  | x |  |  |  |  |  |  |  |  |  |  |  |  |  |  |  |  |  |  |  |  |  |  |  |  |  |  |  |  |  | 1 |
| Kato et al. (2016) | Japan |  |  |  | x |  |  |  |  |  |  |  |  |  |  |  |  |  |  |  |  |  |  |  |  |  |  |  |  |  |  |  |  |  |  | 1 |
| Kazemi Majd et al. (2021) | Iran |  |  |  |  |  | x |  |  |  |  |  |  |  |  |  |  |  |  |  |  |  |  |  |  |  |  |  |  |  |  |  |  |  |  | 1 |
| Kennedy et al. (1987) | USA |  |  |  |  |  |  |  |  |  |  | x |  |  |  |  |  |  |  |  |  |  |  |  |  |  | x |  |  |  |  | x |  |  |  | 3 |
| King (2008) | USA |  |  |  |  |  |  |  |  |  |  |  |  |  |  | x |  |  |  |  |  |  |  |  |  |  |  |  |  |  |  |  |  |  |  | 1 |
| Kitzman et al. (2017) | USA |  |  |  |  |  |  |  |  |  |  |  |  |  |  |  |  |  |  |  |  |  | x |  |  |  |  |  |  |  |  |  |  |  |  | 1 |
| Ko et al. (2017) | South Korea |  |  |  |  |  |  |  |  |  |  |  |  |  |  |  |  |  |  |  |  |  |  |  | x |  |  |  |  |  |  | x |  |  |  | 2 |
| Koehler et al. (2009) | USA |  |  |  |  |  |  | x |  | x |  |  |  |  | x | x |  |  |  | x | x |  |  |  |  | x |  |  | x |  | x |  |  | x |  | 10 |
| Koelling et al. (2005) | USA |  |  |  | x |  |  |  |  |  |  |  |  |  |  |  | x |  |  |  |  |  |  |  |  |  |  |  |  |  |  |  |  |  |  | 2 |
| Kommuri et al. (2012) | USA |  |  |  | x |  |  |  |  |  |  |  |  |  |  |  |  |  |  |  |  |  |  |  |  |  |  |  |  |  |  |  |  |  |  | 1 |
| Koonce et al. (2011) | USA |  |  |  | x |  |  |  |  |  |  |  |  |  |  |  |  |  |  |  |  |  |  |  |  |  |  |  |  |  |  |  |  |  |  | 1 |
| Korkmaz et al. (2020) | Turkey |  |  |  |  |  |  |  |  |  | x |  |  |  |  |  |  |  |  |  |  |  |  |  |  |  |  |  |  |  |  |  |  |  |  | 1 |
| Kowalkowski et al. (2022) | USA |  |  |  |  |  |  |  |  |  |  |  |  |  |  |  |  |  |  |  |  |  |  |  |  |  |  |  |  |  |  | x |  |  |  | 1 |
| Kramer et al. (2007) | USA |  |  |  |  |  |  |  |  |  |  |  |  | x | x |  |  |  |  |  |  |  |  |  |  |  |  |  |  |  |  |  |  |  |  | 2 |
| Kripalani et al. (2012) | USA |  |  |  |  |  |  | x | x |  |  | x |  |  |  |  |  |  |  |  |  | x |  |  |  |  |  |  |  |  |  | x |  |  |  | 5 |
| Kwan et al. (2007) | USA |  |  |  |  |  |  | x |  |  |  |  |  |  |  |  |  |  |  |  |  | x |  |  |  |  |  |  |  |  |  |  |  |  |  | 2 |
| Kwok et al. (2004) | Hong Kong |  |  |  |  |  |  |  |  |  |  |  |  |  | x | x |  |  |  | x |  |  | x |  |  |  |  |  |  |  |  |  | x |  |  | 5 |
| Kwok et al. (2008) | China |  |  |  |  |  |  |  |  |  |  |  |  |  |  |  | x |  |  |  |  |  |  |  |  |  |  |  |  | x |  |  | x |  |  | 3 |
| Lainscak et al. (2013) | Slovenia |  |  | x |  |  | x |  |  |  |  | x |  |  |  |  |  |  |  |  |  |  |  |  |  |  |  | x |  |  |  | x |  |  |  | 5 |
| Lalonde et al. (2008) | Canada |  |  |  |  |  |  |  |  |  |  |  |  |  |  | x |  |  |  |  |  |  |  |  |  |  |  |  |  |  | x |  |  |  |  | 2 |
| Laramee et al. (2003) | USA |  |  | x |  |  | x |  |  |  |  | x |  |  |  |  | x |  |  |  |  |  | x |  |  |  |  | x |  | x |  | x | x |  |  | 9 |
| Latour et al. (2006) | Netherlands |  |  |  |  |  |  |  |  |  |  |  |  |  |  |  |  |  |  |  |  |  |  |  |  |  |  |  |  |  |  | x |  |  |  | 1 |
| Lavesen et al. (2016) | Denmark |  |  |  |  |  |  |  |  |  |  |  |  |  |  |  |  |  |  |  |  |  |  |  |  |  |  |  |  |  |  | x |  |  |  | 1 |
| Lea et al. (2020) | Norway |  |  |  |  |  |  |  |  |  |  |  |  |  |  |  |  |  |  |  |  |  |  |  |  |  |  |  |  |  |  | x |  |  |  | 1 |
| Ledwidge et al. (2005) | Ireland |  |  |  |  |  |  |  |  |  |  |  |  |  |  |  | x |  |  |  |  |  |  |  |  |  |  |  |  |  |  |  |  |  |  | 1 |
| Lee et al. (2015) | Singapore |  |  |  |  |  |  |  |  |  |  |  |  |  |  |  |  |  |  |  |  |  |  |  |  |  |  |  |  |  | x |  |  |  |  | 1 |
| Leff et al. (2009) | USA | x |  |  |  |  |  |  |  |  |  |  |  |  |  |  |  |  |  |  |  |  |  |  |  |  |  |  |  |  |  |  |  |  |  | 1 |
| Legrain et al. (2011) | France |  | x |  |  |  |  |  |  |  |  | x |  |  |  |  |  |  |  |  |  |  | x |  |  | x |  | x |  |  | x | x |  |  |  | 7 |
| Leguelinel-Blache et al. (2014) | France |  |  |  |  |  |  |  |  |  |  |  |  |  |  |  |  |  |  |  |  | x |  |  |  |  |  |  |  |  |  |  |  |  |  | 1 |
| Lembeck et al. (2019) | Denmark |  |  |  |  |  | x |  |  |  |  |  |  |  |  |  |  | x | x |  |  |  |  |  |  |  |  |  |  |  |  | x |  |  |  | 4 |
| Lenssen et al. (2018) | Germany |  |  |  |  |  |  |  |  |  |  |  |  |  |  |  |  |  |  |  |  |  |  |  |  |  |  |  |  |  |  |  |  | x |  | 1 |
| Lenz et al. (2000) | USA |  |  | x |  |  |  |  |  |  |  |  |  |  |  |  |  |  |  |  |  |  |  |  |  |  |  |  |  |  |  |  |  |  |  | 1 |
| Levine et al. (2018) | USA |  |  |  |  |  |  |  |  |  |  |  |  |  |  |  |  |  |  |  |  |  |  |  |  |  |  |  |  |  |  | x |  |  |  | 1 |
| Li et al. (2003) | USA |  |  |  |  |  |  |  |  |  |  |  |  |  |  |  |  |  |  |  |  |  |  |  |  |  |  | x |  |  |  |  |  |  |  | 1 |
| Li et al. (2012) | USA |  |  | x |  |  |  |  |  |  |  |  |  |  |  |  |  |  |  |  |  |  |  |  |  |  |  | x |  |  |  |  |  |  |  | 2 |
| Li et al. (2014) | China |  |  |  |  |  |  |  |  |  |  |  |  |  |  |  |  |  |  |  |  |  |  |  |  |  |  |  |  |  |  | x |  |  |  | 1 |
| Li et al. (2018) | China |  |  |  |  |  |  |  |  |  |  |  |  |  |  |  |  |  |  |  |  |  |  |  |  |  |  |  |  |  |  | x |  |  |  | 1 |
| Li et al. (2021) | China |  |  |  |  |  | x |  |  |  |  |  |  |  |  |  |  |  |  |  |  |  |  |  |  |  |  |  |  |  |  |  |  |  |  | 1 |
| Liang et al. (2021) | Taiwan |  |  |  |  |  | x |  |  |  |  |  |  |  |  |  |  |  |  |  |  |  |  |  |  |  |  |  |  |  |  |  |  |  |  | 1 |
| Lim et al. (2003) | Australia |  | x |  |  |  |  |  |  |  |  |  |  |  |  | x |  |  |  | x |  |  |  |  |  | x |  | x |  |  |  |  | x |  |  | 6 |
| Lim et al. (2013) | Australia |  |  |  |  |  |  |  |  |  |  |  |  |  |  |  |  |  | x |  |  |  |  |  |  |  |  |  |  |  |  |  |  |  |  | 1 |
| Lin et al. (2009) | Taiwan |  |  |  |  |  |  |  |  |  |  | x |  |  |  |  |  |  |  |  |  |  |  |  |  |  |  |  |  |  |  |  |  |  |  | 1 |
| Lin et al. (2014) | Australia |  |  |  | x |  |  |  |  |  |  |  |  |  |  |  |  |  |  |  |  |  |  |  |  |  |  |  |  |  |  | x |  |  |  | 2 |
| Lin et al. (2015) | Hong Kong |  |  |  |  |  |  |  |  | x |  |  |  |  |  |  |  |  |  |  |  |  |  |  |  |  |  |  |  |  |  |  |  |  |  | 1 |
| Lindhardt et al. (2019) | Denmark |  |  |  |  |  | x |  |  |  |  |  |  |  |  |  |  |  |  |  |  |  |  |  |  |  |  |  |  |  |  | x |  |  |  | 2 |
| Lindpaintner et al. (2013) | Slovenia |  |  |  |  |  |  |  |  |  |  | x |  |  |  |  |  |  |  |  |  |  |  |  |  |  |  |  |  |  |  |  |  |  |  | 1 |
| Lipton and Bird (1994) | USA |  |  |  |  | x |  |  |  |  |  |  |  |  |  |  |  |  |  |  |  |  |  |  |  | x |  |  |  |  |  |  |  | x |  | 3 |
| Lisby et al. (2010) | Denmark |  |  |  |  |  |  | x |  |  |  |  |  |  |  |  |  |  |  |  |  |  |  |  |  |  |  |  |  |  |  |  |  | x |  | 2 |
| Lisby et al. (2019) | Denmark |  |  |  |  |  | x |  |  |  |  | x |  |  |  |  |  |  |  |  |  |  |  |  |  |  |  |  |  |  |  |  |  |  |  | 2 |
| Liu et al. (2020) | China |  |  |  |  |  |  |  |  |  |  |  |  |  |  |  |  |  |  |  |  |  |  |  |  |  |  |  |  |  |  | x |  |  |  | 1 |
| Lockwood et al. (2019) | Australia |  |  |  |  |  |  |  |  |  |  |  |  |  |  |  |  |  |  |  |  |  |  |  |  |  |  |  |  |  |  | x |  |  |  | 1 |
| Lopez Cabezas et al. (2006) | Spain |  |  |  |  |  | x |  |  |  |  |  |  |  |  |  |  |  |  |  |  |  |  |  |  |  |  |  |  |  |  |  |  |  |  | 1 |
| Lucas et al. (1998) | USA |  |  |  |  |  |  |  |  |  |  |  |  |  | x |  |  |  |  |  |  |  |  |  |  |  |  |  |  |  |  |  |  |  |  | 1 |
| Ma et al. (2010) | USA |  |  |  |  |  |  |  |  |  |  |  |  |  |  |  |  |  |  |  |  |  |  |  |  |  |  |  |  |  |  |  |  |  | x | 1 |
| Magdaleno et al. (2018) | Spain |  |  |  |  |  |  |  |  |  | x |  |  |  |  |  |  |  |  |  |  |  |  |  |  |  |  |  |  |  |  |  |  |  |  | 1 |
| Magny-Normilus et al. (2021) | USA |  |  |  |  |  | x |  |  |  |  |  |  |  |  |  |  |  |  |  |  |  |  |  |  |  |  |  |  |  |  | x |  |  |  | 2 |
| Makowsky et al. (2009) | Canada |  |  |  |  |  |  |  |  |  |  |  |  | x |  |  |  |  |  |  |  |  |  |  |  | x |  |  | x |  |  |  |  |  |  | 3 |
| Manning et al. (2007) | USA |  |  |  | x |  |  |  |  |  |  |  |  |  |  |  |  |  |  |  |  |  |  |  |  |  |  |  |  |  |  |  |  |  |  | 1 |
| Marotti et al. (2011) | Australia |  |  |  |  |  |  | x |  |  |  |  |  |  |  |  |  |  |  |  |  |  |  |  |  |  |  |  |  |  |  |  |  |  |  | 1 |
| Marsteller et al. (2010) | USA | x |  |  |  |  |  |  |  |  |  |  |  |  |  |  |  |  |  |  |  |  |  |  |  |  |  |  |  |  |  |  |  |  |  | 1 |
| Marsteller et al. (2013) | USA | x |  |  |  |  |  |  |  |  |  |  |  |  |  |  |  |  |  |  |  |  |  |  |  |  |  |  |  |  |  |  |  |  |  | 1 |
| Martin et al. (1994) | UK |  |  |  |  |  |  |  |  |  |  |  |  |  |  |  |  |  |  |  |  |  |  |  |  | x |  |  |  |  |  |  |  |  |  | 1 |
| Marusic et al. (2012) | Croatia |  |  |  |  |  |  |  |  |  |  |  |  |  |  |  |  |  |  |  |  |  |  |  |  | x |  |  |  |  |  |  |  |  |  | 1 |
| Marušić et al. (2013) | Croatia |  |  |  | x |  |  |  |  |  |  |  |  |  |  |  |  |  |  |  |  |  |  |  |  |  |  |  |  |  |  |  |  |  |  | 1 |
| Marušić et al. (2018) | Croatia |  |  |  | x |  |  |  |  |  |  |  |  |  |  |  |  |  |  |  |  |  |  |  |  |  |  |  |  |  |  |  |  |  |  | 1 |
| Maslove et al. (2009) | Canada |  |  |  |  |  |  |  |  |  |  |  |  |  |  | x |  |  |  |  |  |  |  |  |  |  |  |  |  |  |  |  |  |  |  | 1 |
| Mata et al. (2020) | Canada |  |  |  |  |  |  |  |  |  | x |  |  |  |  |  |  |  |  |  |  |  |  |  |  |  |  |  |  |  |  |  |  |  |  | 1 |
| Mayo et al. (2008) | Canada |  |  |  |  |  |  |  |  |  |  |  |  |  |  | x |  |  |  |  |  |  |  |  |  |  |  |  |  |  |  |  |  |  |  | 1 |
| McCarthy et al. (2013) | UK |  |  |  | x |  |  |  |  |  |  |  |  |  |  |  |  |  |  |  |  |  |  |  |  |  |  |  |  |  |  |  |  |  |  | 1 |
| McCorkle et al. (2000) | USA |  |  | x |  |  |  |  |  |  |  |  |  |  |  |  |  |  |  |  |  |  |  |  |  |  |  |  |  |  |  |  |  |  |  | 1 |
| McDonald et al. (2001) | Ireland |  |  |  |  |  |  |  |  |  |  |  |  |  | x |  |  |  |  |  |  |  |  |  |  |  |  |  |  | x |  |  |  |  |  | 2 |
| McDonald et al. (2002) | Ireland |  |  |  |  |  |  |  |  |  |  |  |  |  |  |  | x |  |  |  |  |  |  |  |  |  |  |  |  | x |  |  | x |  |  | 3 |
| McInnes et al. (1999) | Australia |  | x |  |  |  |  |  |  |  |  |  |  |  |  | x |  |  |  |  |  |  |  |  |  |  | x |  |  |  |  |  |  |  |  | 3 |
| McPhee et al. (1983) | USA |  |  |  |  |  |  |  |  |  |  |  |  |  | x |  |  |  |  |  |  |  |  |  |  |  |  |  |  |  |  |  |  |  |  | 1 |
| McWilliams et al. (2019) | USA |  |  |  |  |  |  |  |  |  |  |  |  |  |  |  |  |  |  |  |  |  |  |  |  |  |  |  |  |  |  | x |  |  |  | 1 |
| Mehta et al. (2020) | USA |  |  |  |  |  |  |  |  |  |  |  |  |  |  |  |  |  |  |  |  |  |  |  |  |  |  |  |  |  |  | x |  |  |  | 1 |
| Meisinger et al. (2013) | Germany |  |  |  |  |  |  |  |  |  |  |  |  |  |  |  |  |  |  |  |  |  |  |  |  |  |  |  |  |  |  | x |  |  |  | 1 |
| Mesbahi et al. (2020) | Iran |  |  |  |  |  |  |  |  |  |  |  |  |  |  |  |  |  |  |  |  |  |  | x |  |  |  |  |  |  |  |  |  |  |  | 1 |
| Misky et al. (2010) | USA |  |  |  |  |  |  |  |  |  |  |  |  |  | x |  |  |  |  |  |  |  |  |  |  |  |  |  |  |  |  |  |  |  |  | 1 |
| Moher et al. (1992) | Canada |  |  |  |  |  |  |  |  |  |  | x |  |  |  |  |  |  |  |  |  |  |  |  |  |  |  |  |  |  |  | x |  |  |  | 2 |
| Moore et al. (2015) | USA |  |  |  | x |  |  |  |  |  |  |  |  |  |  |  |  |  |  |  |  |  |  |  |  |  |  |  |  |  |  |  |  |  |  | 1 |
| Morice et al. (2001) | UK |  |  |  | x |  |  |  |  |  |  |  |  |  |  |  |  |  |  |  |  |  |  |  |  |  |  |  |  |  |  |  |  |  |  | 1 |
| Mortimer et al. (2010) | Australia |  |  |  |  |  |  |  |  |  |  |  |  |  |  |  |  |  |  |  |  |  |  |  |  |  |  |  | x |  |  |  |  |  |  | 1 |
| Moss et al. (2014) | UK |  |  |  | x |  |  |  |  |  |  |  |  |  |  |  |  |  |  |  |  |  |  |  |  |  |  |  |  |  |  |  |  |  |  | 1 |
| Mudge et al. (2006) | Australia |  |  |  |  |  |  |  |  |  |  |  |  |  |  |  |  |  |  |  |  |  |  |  |  | x |  |  |  |  |  |  |  |  |  | 1 |
| Murphy et al. (2019) | USA |  |  |  |  |  |  |  |  |  |  |  |  |  |  |  |  |  |  |  |  |  |  |  |  |  |  |  |  |  |  |  |  |  | x | 1 |
| Muyiwa-Ojo (2018) | USA |  |  |  |  |  |  |  |  |  |  |  |  |  |  |  |  |  |  |  |  |  |  | x |  |  |  |  |  |  |  |  |  |  |  | 1 |
| Naderloo et al. (2018) | Iran |  |  |  | x |  |  |  |  |  |  |  |  |  |  |  |  |  |  |  |  |  |  |  |  |  |  |  |  |  |  |  |  |  |  | 1 |
| Naji et al. (1999) | Scotland |  |  |  |  |  |  |  |  |  |  | x |  |  |  |  |  |  |  |  |  |  |  |  |  |  |  |  |  |  |  |  |  |  |  | 1 |
| Naughton et al. (1994) | USA |  |  |  |  |  |  |  |  |  |  | x |  |  |  |  |  |  |  |  |  |  |  |  |  |  |  |  |  |  |  |  |  |  |  | 1 |
| Naunton et al. (2003) | Australia |  |  |  |  |  | x |  |  |  |  |  |  |  |  |  |  |  |  |  |  |  |  |  |  |  |  |  |  |  |  |  |  |  |  | 1 |
| Naylor et al. (1990) | USA |  | x |  |  |  |  |  |  |  |  |  |  |  |  |  |  |  |  |  |  |  |  |  |  | x |  |  |  |  |  |  |  |  |  | 2 |
| Naylor et al. (1994) | USA | x | x |  |  |  | x |  |  |  |  | x |  |  | x |  |  |  |  |  |  |  | x |  |  |  | x | x |  |  |  | x |  |  |  | 9 |
| Naylor et al. (1999) | USA | x | x | x |  |  | x |  |  |  |  |  |  |  |  | x |  |  |  |  |  |  | x |  |  | x | x | x |  |  |  |  | x |  |  | 10 |
| Naylor et al. (2004) | USA | x | x | x |  |  | x |  |  |  |  |  |  |  |  | x |  |  | x | x |  |  | x |  |  |  |  | x |  | x |  |  | x |  |  | 11 |
| Nazareth et al. (2001) | UK |  |  |  |  | x |  |  |  |  |  | x |  |  |  | x |  |  |  |  |  |  |  |  |  | x |  |  |  |  |  | x |  | x |  | 6 |
| Negarandeh et al. (2019) | Iran |  |  |  |  |  |  |  |  |  |  |  |  |  |  |  |  |  |  |  |  |  |  |  |  |  |  |  |  |  |  | x |  |  |  | 1 |
| Nguyen et al. (2018) | Vietnam |  |  |  |  |  | x |  | x |  |  | x |  |  |  |  |  |  |  |  |  |  |  |  |  |  |  |  |  |  |  | x |  |  |  | 4 |
| Nickerson et al. (2005) | Canada |  |  |  |  |  |  | x |  |  |  |  |  |  |  |  |  |  |  |  |  | x |  |  |  |  |  |  |  |  |  |  |  |  |  | 2 |
| Nielsen et al. (2018) | Denmark |  |  |  |  |  |  |  |  | x |  |  |  |  |  |  |  |  |  |  |  |  |  |  |  |  |  |  |  |  |  |  |  |  |  | 1 |
| Nikolaus et al. (1995) | Germany |  |  |  |  |  |  |  |  |  |  |  |  |  |  |  |  |  |  |  |  |  |  |  |  |  | x |  |  |  |  |  |  |  |  | 1 |
| Nikolaus et al. (1999) | Germany |  |  |  |  |  |  |  |  |  |  |  |  |  |  |  |  | x |  |  |  |  |  |  |  | x | x |  |  |  |  |  | x |  |  | 4 |
| Noel et al. (2020) | USA |  |  |  |  |  |  |  |  |  |  |  |  |  |  |  |  |  |  |  |  |  |  |  |  |  |  |  |  |  |  | x |  |  |  | 1 |
| Nucifora et al. (2006) | Italy |  |  | x |  |  | x |  |  |  |  |  |  |  |  |  |  |  |  |  |  |  |  |  |  |  |  |  |  | x |  |  | x |  |  | 4 |
| Nylor et al. (2004) | USA |  |  |  |  |  |  |  |  |  |  |  |  |  |  |  | x |  |  |  |  |  |  |  |  |  |  |  |  |  |  |  |  |  |  | 1 |
| O'Connell et al. (2018) | UK |  |  |  |  |  |  |  |  |  |  |  |  |  |  |  |  |  |  |  |  |  |  |  |  |  |  |  |  |  |  | x |  |  |  | 1 |
| Odeh et al. (2020) | Ireland |  |  |  |  |  |  |  |  |  |  |  |  |  |  |  |  |  |  |  |  |  |  |  |  |  |  |  |  |  |  | x |  |  |  | 1 |
| O'Dell and Kucukarslan (2005) | USA |  |  |  |  |  |  |  |  |  |  |  |  |  | x |  |  |  |  |  |  |  |  |  |  |  |  |  |  |  |  |  |  |  |  | 1 |
| Ohuabunwa et al. (2013) | USA |  |  |  |  |  |  |  |  |  |  |  |  |  |  |  |  |  |  |  |  |  | x |  |  |  |  |  |  |  |  |  |  |  |  | 1 |
| Ojeda et al. (2005) | Spain |  |  |  |  |  |  |  |  |  |  |  |  |  |  |  | x |  |  |  |  |  |  |  |  |  |  |  |  |  |  |  |  |  |  | 1 |
| Okere et al. (2016) | USA |  |  |  |  |  |  |  |  |  |  |  |  |  |  |  |  |  |  |  |  |  |  |  |  |  |  |  | x |  |  |  |  |  |  | 1 |
| Oliveira-Filho et al. (2014) | Brazil |  |  |  | x | x | x |  |  |  |  |  |  |  |  |  |  |  |  |  |  |  |  |  |  |  |  |  |  |  |  |  |  |  |  | 3 |
| Olives et al. (2016) | USA |  |  |  | x |  |  |  |  |  |  |  |  |  |  |  |  |  |  |  |  |  |  |  |  |  |  |  |  |  |  |  |  |  |  | 1 |
| Ong et al. (2016) | USA |  |  |  |  |  | x |  |  |  |  |  |  |  |  |  |  |  |  |  |  |  |  |  |  |  |  |  |  |  |  | x |  |  |  | 2 |
| Ornstein et al. (2011) | USA |  |  |  |  |  |  |  |  |  |  |  |  |  |  |  |  |  | x |  |  |  |  |  |  |  |  |  |  |  |  |  |  |  |  | 1 |
| Oscalices et al. (2019) | Brazil |  |  |  |  |  |  |  |  |  |  |  |  |  |  |  |  |  |  |  |  |  |  |  |  |  |  |  |  |  |  | x |  |  |  | 1 |
| Osman et al. (2002) | UK |  |  |  | x |  |  |  |  |  |  |  |  |  |  |  |  |  |  |  |  |  |  |  |  |  |  |  |  |  |  |  |  |  |  | 1 |
| Ougrin et al. (2020) | UK |  |  |  |  |  |  |  |  |  |  |  |  |  |  |  |  |  |  |  |  |  |  |  |  |  |  |  |  |  |  | x |  |  |  | 1 |
| Pal et al. (2013) | USA |  |  |  |  |  |  |  |  |  |  |  |  |  |  |  |  |  |  |  | x |  |  |  |  |  |  |  |  |  |  |  |  |  |  | 1 |
| Palmer et al. (2002) | USA |  |  |  |  |  |  |  |  |  |  |  |  |  |  |  |  |  |  |  |  |  |  |  |  | x |  |  |  |  |  |  |  |  |  | 1 |
| Pardessus et al. (2002) | France |  |  |  |  |  |  |  |  |  |  |  |  |  |  |  |  |  |  |  |  |  |  |  |  |  |  |  |  |  |  | x |  |  |  | 1 |
| Parfrey et al. (1994) | Canada |  |  |  |  |  |  |  |  |  |  | x |  |  |  |  |  |  |  |  |  |  |  |  |  |  |  |  |  |  |  |  |  |  |  | 1 |
| Parry et al. (2009) | USA |  |  |  |  |  |  |  |  |  |  |  |  |  | x |  |  |  | x |  |  |  |  |  |  | x |  |  |  |  |  |  | x |  |  | 4 |
| Parsons et al. (2020) | New Zealand |  |  |  |  |  |  |  |  |  |  |  |  |  |  |  |  |  |  |  |  |  |  |  |  |  |  |  |  |  |  | x |  |  |  | 1 |
| Pearson et al. (2006) | Australia |  |  |  |  |  | x |  |  |  |  |  |  |  |  |  |  |  |  |  |  |  |  |  |  |  |  |  |  |  |  |  |  |  |  | 1 |
| Pellegrin et al. (2017) | USA |  |  |  |  |  |  |  |  |  |  |  |  |  |  |  |  |  |  |  |  |  |  |  |  |  |  |  |  |  |  |  |  | x |  | 1 |
| Perennes et al. (2012) | France |  |  |  |  |  |  |  |  |  |  |  |  | x |  |  |  |  |  |  |  |  |  |  |  |  |  |  |  |  |  |  |  |  |  | 1 |
| Perera et al. (2012) | Sri Lanka |  |  |  | x |  |  |  |  |  |  |  |  |  |  |  |  |  |  |  |  |  |  |  |  |  |  |  |  |  |  |  |  |  |  | 1 |
| Phatak et al. (2016) | USA |  |  |  |  | x |  |  | x |  |  |  |  |  |  |  |  |  |  |  |  |  |  |  |  |  |  |  |  |  |  | x |  |  |  | 3 |
| Piette et al. (2016) | USA |  |  |  |  |  | x |  |  |  |  |  |  |  |  |  |  |  |  |  |  |  |  |  |  |  |  |  |  |  |  |  |  |  |  | 1 |
| Piette et al. (2020) | USA |  |  |  |  |  |  |  |  |  |  |  |  |  |  |  |  |  |  |  |  |  |  |  |  |  |  |  |  |  |  | x |  |  |  | 1 |
| Piette, Striplin, Fisher et al. (2020) | USA |  |  |  |  |  | x |  |  |  |  |  |  |  |  |  |  |  |  |  |  |  |  |  |  |  |  |  |  |  |  |  |  |  |  | 1 |
| Pourrat et al. (2020) | France |  |  |  |  |  |  |  |  |  |  |  |  |  |  |  |  |  |  |  |  |  |  |  |  |  |  |  |  |  |  | x |  |  |  | 1 |
| Preen et al. (2005) | Australia |  | x |  |  |  |  |  |  |  |  | x |  |  |  | x |  |  |  |  |  |  | x |  |  |  |  |  |  |  |  |  |  |  |  | 4 |
| Press et al. (2012) | USA |  |  |  | x |  |  |  |  |  |  |  |  |  |  |  |  |  |  |  |  |  |  |  |  |  |  |  |  |  |  |  |  |  |  | 1 |
| Press et al. (2016) | USA |  |  |  | x |  |  |  |  |  |  |  |  |  |  |  |  |  |  |  |  |  |  |  |  |  |  |  |  |  |  |  |  |  |  | 1 |
| Puligano et al. (2010) | Italy |  |  |  |  |  |  |  |  |  |  |  |  |  |  |  |  | x |  |  |  |  |  |  |  |  |  |  |  |  |  |  |  |  |  | 1 |
| Qian et al. (2019) | China |  |  |  |  |  |  |  |  |  |  |  |  |  |  |  |  |  |  |  |  |  |  |  |  |  |  |  |  |  |  | x |  |  |  | 1 |
| Rabi and Dahdal (2007) | USA |  |  |  |  |  |  |  |  |  |  |  |  | x |  |  |  |  |  |  |  |  |  |  |  |  |  |  |  |  |  |  |  |  |  | 1 |
| Rafferty et al. (2016) | USA |  |  |  |  |  |  |  |  |  |  |  |  |  |  |  |  |  |  |  |  |  |  |  |  |  |  |  | x |  |  |  |  |  |  | 1 |
| Rahmani (2020) | Iran |  |  |  |  |  |  |  |  |  |  |  |  |  |  |  |  |  |  |  |  |  |  | x |  |  |  |  |  |  |  |  |  |  |  | 1 |
| Rainville et al. (1999) | USA |  |  |  |  |  |  |  |  |  |  |  |  |  | x |  |  |  |  |  |  |  |  |  |  |  |  |  |  |  |  |  |  |  |  | 1 |
| Ravn-Nielsen et al. (2018) | Denmark |  |  |  |  | x |  |  |  |  |  |  |  |  |  |  |  |  |  |  |  |  |  |  |  |  |  |  | x |  | x | x |  | x |  | 5 |
| Raynor et al. (1993) | UK |  |  |  | x |  |  |  |  |  |  |  |  |  |  |  |  |  |  |  |  |  |  |  |  |  |  |  |  |  |  |  |  |  |  | 1 |
| Rebello et al. (2017) | USA |  |  |  |  |  |  |  |  |  |  |  |  |  |  |  |  |  | x |  |  |  |  |  |  |  |  |  |  |  |  |  |  |  |  | 1 |
| Reuben et al. (1995) | USA |  |  |  |  |  |  |  |  |  |  |  |  |  |  |  |  | x |  |  |  |  |  |  |  |  | x |  |  |  |  |  |  |  |  | 2 |
| Reynolds et al. (2004) | Scotland |  |  |  |  |  |  |  |  |  |  |  |  |  |  |  |  |  |  |  |  |  |  |  |  |  |  |  |  |  |  | x |  |  |  | 1 |
| Rich et al. (1993) | USA |  |  |  |  |  |  |  |  |  |  | x | x |  |  | x |  |  |  |  |  |  |  |  |  |  |  |  |  | x |  | x |  |  |  | 5 |
| Rich et al. (1995) | USA |  |  |  |  |  |  |  |  |  |  | x | x |  |  |  | x |  |  |  |  |  |  |  |  |  |  | x |  | x |  | x | x |  |  | 7 |
| Ritchie et al. (2016) | USA |  |  |  |  |  |  |  |  |  |  |  |  |  |  |  |  |  |  |  |  |  |  |  |  |  |  |  |  |  |  | x |  |  |  | 1 |
| Robinson et al. (2015) | New Zealand |  |  |  |  |  |  |  |  | x |  |  |  |  |  |  |  |  |  |  |  |  |  |  |  |  |  |  |  |  |  |  |  |  |  | 1 |
| Rose et al. (2018) | Canada |  |  |  |  |  |  |  |  |  |  |  |  |  |  |  |  |  |  |  |  |  |  |  | x |  |  |  |  |  |  |  |  |  |  | 1 |
| Rottman-Sagebiel et al. (2018) | USA |  |  |  |  |  |  |  |  | x |  |  |  |  |  |  |  |  |  |  |  |  |  |  |  |  |  |  |  |  |  |  |  |  |  | 1 |
| Rubenstein et al. (1984) | USA |  |  |  |  |  |  |  |  |  |  |  |  |  |  |  |  |  |  |  |  |  |  |  |  |  | x |  |  |  |  |  |  |  |  | 1 |
| Rubenstein et al. (1995) | USA |  |  |  |  |  |  |  |  |  |  |  |  |  |  |  |  |  |  |  |  |  |  |  |  |  | x |  |  |  |  |  |  |  |  | 1 |
| Rubin et al. (1992) | USA |  |  |  |  |  |  |  |  |  |  |  |  |  |  |  |  |  |  |  |  |  |  |  |  |  | x |  |  |  |  |  |  |  |  | 1 |
| Rubin et al. (1993) | USA |  |  |  |  |  |  |  |  |  |  |  |  |  |  |  |  |  |  |  |  |  |  |  |  |  | x |  |  |  |  |  |  |  |  | 1 |
| Rubin et al. (1994) | USA |  |  |  |  |  |  |  |  |  |  |  |  |  |  |  |  |  |  |  |  |  |  |  |  |  | x |  |  |  |  |  |  |  |  | 1 |
| Russell et al. (2011) | USA | x |  |  |  |  |  |  |  |  |  |  |  |  |  |  |  |  |  |  |  |  |  |  |  |  |  |  |  | x |  |  |  |  |  | 2 |
| Rutherford and Burge (2001) | Australia |  |  |  |  |  |  |  |  |  |  |  |  |  |  | x |  |  |  |  |  |  |  |  |  |  |  |  |  |  |  |  |  |  |  | 1 |
| Rytter et al. (2010) | Denmark |  |  |  |  |  |  |  |  |  |  |  |  |  |  | x |  | x |  |  |  |  |  |  |  |  |  |  |  |  |  |  |  |  |  | 2 |
| Saez De La Fuente et al. (2011) | Spain |  |  |  | x |  |  |  |  |  |  |  |  |  |  |  |  |  |  |  |  |  |  |  |  |  |  |  |  |  |  |  |  |  |  | 1 |
| Sahota et al. (2017) | UK |  |  |  |  |  |  |  |  | x |  |  |  |  |  |  |  | x |  |  |  |  |  |  |  |  |  |  |  |  |  | x |  |  |  | 3 |
| Salameh et al. (2018) | Jordan |  |  |  |  | x |  |  |  |  |  |  |  |  |  |  |  |  |  |  |  |  |  |  |  |  |  |  |  |  |  | x |  |  |  | 2 |
| Saleh et al. (2012) | USA | x |  |  |  |  |  |  |  |  |  |  |  |  |  |  |  |  |  |  |  |  |  |  |  |  |  |  |  |  |  |  | x |  |  | 2 |
| Sales et al. (2013) | USA |  |  |  |  |  | x |  |  |  |  |  |  |  |  |  |  |  |  |  |  |  |  |  |  |  |  |  |  |  |  |  |  |  |  | 1 |
| Sales et al. (2014) | USA |  |  |  |  |  |  |  |  |  |  |  |  |  |  |  |  |  |  |  |  |  |  |  |  |  |  |  |  |  |  | x |  |  |  | 1 |
| Salmany et al. (2018) | Jordan |  |  |  |  |  |  |  |  |  |  |  |  |  |  |  |  |  |  |  |  |  |  |  |  |  |  |  |  |  |  | x |  |  |  | 1 |
| Sanchez et al. (2012) | Spain |  |  |  |  | x |  |  |  |  |  |  |  |  |  |  |  |  |  |  |  |  |  |  |  |  |  |  |  |  |  |  |  |  |  | 1 |
| Sanchez Ulayar et al. (2012) | Spain |  |  |  | x |  |  |  |  |  |  |  |  |  |  |  |  |  |  |  |  |  |  |  |  |  |  |  |  |  |  |  |  |  |  | 1 |
| Sanii et al. (2016) | Iran |  |  |  | x |  |  |  |  |  |  |  |  |  |  |  |  |  |  |  |  |  |  |  |  |  |  |  |  |  |  |  |  |  |  | 1 |
| Santana et al. (2017) | Portugal |  |  |  |  |  |  |  |  |  |  |  |  |  |  |  |  |  |  |  |  |  | x |  |  |  |  |  |  |  |  | x |  |  |  | 2 |
| Sarangarm et al. (2013) | USA |  |  |  |  |  |  |  | x |  |  |  |  |  |  |  |  |  |  |  |  |  |  |  |  |  |  |  |  |  |  |  |  |  |  | 1 |
| Schapira et al. (2021) | Argentina |  |  |  |  |  |  |  |  |  |  |  |  |  |  |  |  | x |  |  |  |  |  |  |  |  |  |  |  |  |  |  |  |  |  | 1 |
| Schneider et al. (1993) | USA |  |  |  |  |  | x |  |  |  |  |  |  |  | x |  |  |  |  |  |  |  |  |  |  |  |  |  |  |  |  |  |  |  |  | 2 |
| Schnipper et al. (2006) | USA |  |  |  |  | x |  | x |  |  |  |  |  |  |  | x |  |  |  |  | x |  |  |  |  | x |  |  |  |  |  |  |  |  |  | 5 |
| Schnipper et al. (2009) | USA |  |  |  |  |  |  |  |  |  |  |  |  |  |  | x |  |  |  |  |  |  |  |  |  |  |  |  |  |  |  |  |  |  |  | 1 |
| Schnipper et al. (2021) | USA |  |  |  |  |  | x |  |  |  |  |  |  |  |  |  |  |  |  |  |  |  |  |  |  |  |  |  |  |  |  |  |  |  |  | 1 |
| Scnipper et al. (2006) | USA |  |  |  |  |  |  |  |  |  |  |  |  |  |  |  |  |  |  |  |  | x |  |  |  |  |  |  |  |  |  |  |  |  |  | 1 |
| Scullin et al. (2007) | Ireland |  |  |  |  | x |  | x |  |  |  |  |  | x |  |  |  |  |  |  | x |  |  |  |  | x |  |  | x |  | x |  |  |  |  | 7 |
| Scullin et al. (2011) | Ireland |  |  |  |  |  |  |  |  |  |  |  |  |  |  |  |  |  |  |  |  |  |  |  |  | x |  |  |  |  |  |  |  |  |  | 1 |
| Scullin et al. (2012) | UK |  |  |  |  |  |  |  |  |  |  |  |  |  |  |  |  |  |  |  |  |  |  |  |  |  |  |  | x |  |  |  |  |  |  | 1 |
| Serxner et al. (1998) | USA |  |  |  |  |  |  |  |  |  |  |  | x |  |  |  |  |  |  |  |  |  |  |  |  |  |  |  |  |  |  |  |  |  |  | 1 |
| Sethares and Elliot (2004) | USA |  |  |  |  |  |  |  |  |  |  |  |  |  |  |  | x |  |  |  |  |  |  |  |  |  |  |  |  |  |  |  |  |  |  | 1 |
| Shah et al. (2013) | USA |  |  |  | x |  |  |  |  |  |  |  |  |  |  |  |  |  |  |  |  |  |  |  |  |  |  |  |  |  |  |  |  |  |  | 1 |
| Shahrokhi et al. (2017) | Iran |  |  |  |  |  |  |  |  |  |  |  |  |  |  |  |  |  |  |  |  |  |  |  |  |  |  |  |  |  |  | x |  |  |  | 1 |
| Shahrokhi et al. (2018) | Iran |  |  |  |  |  | x |  |  |  |  |  |  |  |  |  |  |  |  |  |  |  |  |  |  |  |  |  |  |  |  |  |  |  |  | 1 |
| Shakib et al. (2016) | Australia |  |  |  |  |  |  |  |  |  |  |  |  |  |  |  |  |  | x |  |  |  |  |  |  |  |  |  |  |  |  |  |  |  |  | 1 |
| Sharma et al. (2010) | USA |  |  |  |  |  |  |  |  |  |  |  |  |  | x |  |  |  |  |  |  |  |  |  |  |  |  |  |  |  |  |  |  |  |  | 1 |
| Shaw et al. (2000) | Scotland |  |  |  |  | x |  |  |  |  |  | x |  |  |  |  |  |  |  |  |  |  |  |  |  |  |  |  |  |  |  | x |  |  |  | 3 |
| Shyu et al. (2005) | Taiwan |  |  |  |  |  |  |  |  |  |  |  |  |  |  |  |  |  |  |  |  |  |  |  |  |  |  | x |  |  |  |  |  |  |  | 1 |
| Shyu et al. (2008) | China |  |  |  |  |  |  |  |  |  |  |  |  |  |  |  |  |  |  |  |  |  | x |  |  |  |  |  |  |  |  |  |  |  |  | 1 |
| Shyu et al. (2010) | Taiwan |  |  |  |  |  |  |  |  |  |  |  |  |  |  | x |  |  |  |  |  |  |  |  |  |  |  | x |  |  |  |  |  |  |  | 2 |
| Sier et al. (2017) | Netherlands |  |  |  |  |  |  |  |  |  | x |  |  |  |  |  |  |  |  |  |  |  |  |  |  |  |  |  |  |  |  |  |  |  |  | 1 |
| Silliman et al. (1990) | USA |  |  |  |  |  |  |  |  |  |  |  |  |  |  |  |  |  |  |  |  |  |  |  |  |  | x |  |  |  |  |  |  |  |  | 1 |
| Silver et al. (2017) | USA |  |  |  |  |  |  |  |  |  |  |  |  |  |  |  |  |  |  |  |  |  |  |  | x |  |  |  |  |  |  |  |  |  |  | 1 |
| Simorangkir et al. (2017) | Indonesia |  |  |  |  |  |  |  | x |  |  |  |  |  |  |  |  |  |  |  |  |  |  |  |  |  |  |  |  |  |  |  |  |  |  | 1 |
| Simpson et al. (2019) | USA |  |  |  |  |  |  |  |  |  |  |  |  |  |  |  |  |  | x |  |  |  |  |  |  |  |  |  |  |  |  |  |  |  |  | 1 |
| Siu et al. (1996) | USA |  |  |  |  |  |  |  |  |  |  |  |  |  |  | x |  | x |  |  |  |  |  |  |  | x | x |  |  |  |  |  | x |  |  | 5 |
| Smith et al. (1988) | USA |  |  |  |  |  |  |  |  |  |  |  |  |  |  |  |  |  |  |  |  |  |  |  |  |  |  |  |  |  |  |  | x |  |  | 1 |
| Smith et al. (1995) | USA |  |  |  |  |  |  |  |  |  |  |  |  |  | x |  |  |  |  |  |  |  |  |  |  |  |  |  |  |  |  |  |  |  |  | 1 |
| Smith et al. (1997) | UK |  |  |  | x |  |  |  |  |  |  |  |  |  |  |  |  |  |  |  |  |  |  |  |  |  |  |  |  |  |  |  |  |  |  | 1 |
| Song et al. (2021) | China |  |  |  |  |  |  |  |  |  | x |  |  |  |  |  |  |  |  |  |  |  |  |  |  |  |  |  |  |  |  |  |  |  |  | 1 |
| Sorknaes et al. (2011) | Denmark |  |  |  |  |  |  |  |  |  |  |  |  |  | x |  |  |  |  |  |  |  |  |  |  |  |  |  |  |  |  |  |  |  |  | 1 |
| Spinewine et al. (2007) | Belgium |  |  |  |  |  |  |  |  |  |  |  |  |  |  |  |  |  |  |  |  |  |  |  |  |  |  |  | x |  |  |  |  | x |  | 2 |
| Stauffer et al. (2011) | USA | x |  |  |  |  |  |  |  |  |  |  |  |  |  |  |  |  |  |  |  |  |  |  |  |  |  |  |  | x |  |  |  |  |  | 2 |
| Steeman et al. (2006) | Belgium |  |  |  |  |  |  |  |  |  |  |  |  |  | x |  |  |  |  |  |  |  |  |  |  | x |  |  |  |  |  |  |  |  |  | 2 |
| Stewart et al. (1998) | Australia |  |  |  |  |  |  |  |  |  |  |  | x |  |  |  | x |  |  |  |  |  |  |  |  | x |  |  |  | x |  |  |  |  |  | 4 |
| Stewart et al. (1999) | USA |  |  |  |  |  |  |  |  |  |  |  |  |  |  |  |  |  |  |  |  |  |  |  |  |  |  |  |  | x |  |  |  |  |  | 1 |
| Stowasser et al. (2002) | Australia |  |  |  |  |  |  | x |  |  |  |  |  | x |  |  |  |  |  |  | x |  |  |  |  |  |  |  |  |  |  |  |  |  |  | 3 |
| Strano et al. (2019) | USA |  |  |  |  |  |  |  |  |  |  |  |  |  |  |  |  |  |  |  |  |  |  |  |  |  |  |  |  |  |  | x |  |  |  | 1 |
| Strobach et al. (2000) | Germany |  |  |  | x |  |  |  |  |  |  |  |  |  |  |  |  |  |  |  |  |  |  |  |  |  |  |  |  |  |  |  |  |  |  | 1 |
| Styrborn et al. (1995) | Sweden |  |  |  |  |  |  |  |  |  |  |  |  |  |  |  |  |  |  |  |  |  |  |  |  | x |  |  |  |  |  |  |  |  |  | 1 |
| Sudas Na Ayutthaya et al. (2017) | Thailand |  |  |  |  |  |  |  |  |  |  |  |  |  |  |  |  |  |  |  |  |  |  |  |  |  |  |  |  |  |  | x |  |  |  | 1 |
| Sulch et al. (2000) | UK |  |  |  |  |  |  |  |  |  |  | x |  |  |  |  |  |  |  |  |  |  |  |  |  |  |  |  |  |  |  |  |  |  |  | 1 |
| Surepill et al. (2015) | UK |  |  |  |  |  |  |  |  |  |  |  |  |  |  |  |  |  |  |  |  |  |  |  |  |  |  |  | x |  |  |  |  |  |  | 1 |
| Tamblyn et al. (2019) | Canada |  |  |  |  |  |  |  |  |  |  |  |  |  |  |  |  |  |  |  |  |  |  |  |  |  |  |  |  |  | x |  |  |  |  | 1 |
| Tanajewski et al. (2015) | UK |  |  |  |  |  |  |  |  |  |  |  |  |  |  |  |  | x |  |  |  |  |  |  |  |  |  |  |  |  |  |  |  |  |  | 1 |
| Thomas et al. (1993) | USA |  |  |  |  |  |  |  |  |  |  |  |  |  |  |  |  |  |  |  |  |  |  |  |  | x | x |  |  |  |  |  |  |  |  | 2 |
| Thompson et al. (2005) | UK |  |  |  |  |  |  |  |  |  |  |  |  |  |  |  | x |  |  |  |  |  |  |  |  |  |  |  |  |  |  |  |  |  |  | 1 |
| Thygesen et al. (2015) | Denmark |  |  |  |  |  |  |  |  |  |  |  |  |  |  |  |  |  |  |  |  |  |  |  |  |  |  |  |  |  |  | x |  |  |  | 1 |
| Toles et al. (2017) | USA |  |  |  |  |  |  |  |  |  |  |  |  |  |  |  |  |  |  |  |  |  | x |  |  |  |  |  |  |  |  |  |  |  |  | 1 |
| Tomita et al. (2012) | USA |  |  |  |  |  |  |  |  |  |  |  |  |  |  |  |  |  |  |  |  |  |  |  |  |  |  |  |  |  |  | x |  |  |  | 1 |
| Tompson et al. (2012) | Australia |  |  |  |  |  |  |  |  |  |  |  |  |  |  |  |  |  |  |  |  | x |  |  |  |  |  |  |  |  |  |  |  |  |  | 1 |
| Tong et al. (2017) | Australia |  |  |  |  |  |  |  |  |  |  |  |  |  |  |  |  |  |  |  |  |  |  |  |  |  |  |  |  |  | x |  |  |  |  | 1 |
| Townsend et al. (1988) | UK |  |  |  |  |  |  |  |  |  |  |  |  |  |  |  |  |  |  |  |  |  |  |  |  |  |  |  |  |  |  |  | x |  |  | 1 |
| Trentini et al. (1995) | Italy |  |  |  |  |  |  |  |  |  |  |  |  |  |  |  |  |  |  |  |  |  |  |  |  |  | x |  |  |  |  |  |  |  |  | 1 |
| Tripp (2009) | USA |  |  |  |  |  |  |  |  |  |  |  |  |  |  | x |  |  |  |  |  |  |  |  |  |  |  |  |  |  |  |  |  |  |  | 1 |
| Tsuyuki et al. (2004) | Canada |  |  |  |  | x |  |  |  |  |  |  |  |  |  |  |  |  |  |  |  |  |  |  |  |  |  |  |  |  |  |  |  |  |  | 1 |
| Tu et al. (2020) | China |  |  |  |  |  | x |  |  |  |  |  |  |  |  |  |  |  |  |  |  |  |  |  |  |  |  |  |  |  |  |  |  |  |  | 1 |
| Tuttle et al. (2018) | n.a. |  |  |  |  |  |  |  |  |  |  |  |  |  |  |  |  |  |  |  |  |  |  |  |  |  |  |  |  |  | x |  |  |  |  | 1 |
| Utens et al. (2012) | Netherlands |  |  |  |  |  |  |  |  |  |  |  |  |  |  |  |  |  |  |  |  |  |  |  |  |  |  |  |  |  |  | x |  |  |  | 1 |
| Van den Bemt et al. (2009) | Netherlands |  |  |  |  |  |  |  |  |  |  |  |  |  |  |  |  |  |  |  |  | x |  |  |  |  |  |  |  |  |  |  |  |  |  | 1 |
| Van den Bemt et al. (2013) | Netherlands |  |  |  |  |  |  |  |  |  |  |  |  |  |  |  |  |  |  |  |  | x |  |  |  |  |  |  |  |  |  |  |  |  |  | 1 |
| Van der Hejiden et al. (2019) | Netherlands |  |  |  |  |  |  |  |  |  |  |  |  |  |  |  |  |  |  |  |  |  |  |  |  |  |  |  |  |  |  | x |  |  |  | 1 |
| Van der Linden et al. (2017) | Belgium |  |  |  |  |  |  |  |  |  |  |  |  |  |  |  |  |  |  |  |  |  |  |  |  |  |  |  | x |  |  |  |  | x |  | 2 |
| Van Spall et al. (2019) | Canada |  |  |  |  |  | x |  |  |  |  |  |  |  |  |  |  |  |  |  |  |  | x |  |  |  |  |  |  |  |  | x |  |  |  | 3 |
| Van Walraven et al. (1999) | Canada |  |  |  |  |  |  |  |  |  |  |  |  |  |  | x |  |  |  |  |  |  |  |  |  |  |  |  |  |  |  |  |  |  |  | 1 |
| Van Walraven et al. (2004) | Canada |  |  |  |  |  |  |  |  |  |  |  |  |  | x |  |  |  |  |  |  |  |  |  |  |  |  |  |  |  |  |  |  |  |  | 1 |
| Vasileff et al. (2009) | Australia |  |  |  |  |  |  |  |  |  |  |  |  |  |  |  |  |  |  |  |  | x |  |  |  |  |  |  |  |  |  |  |  |  |  | 1 |
| Vesterby et al. (2017) | Denmark |  |  |  |  |  |  |  |  |  |  |  |  |  |  |  |  |  |  |  |  |  |  |  |  |  |  |  |  |  |  | x |  |  |  | 1 |
| Villars et al. (2013) | France |  |  |  |  |  |  |  |  |  |  |  |  |  |  |  |  |  | x |  |  |  |  |  |  |  |  |  |  |  |  |  |  |  |  | 1 |
| Vinluan et al. (2015) | USA |  |  |  |  |  |  |  |  |  |  |  |  |  |  |  |  |  |  |  |  |  |  |  |  |  |  |  |  |  |  | x |  |  |  | 1 |
| Vira et al. (2006) | Canada |  |  |  |  |  |  |  |  |  |  |  |  | x |  |  |  |  |  |  |  |  |  |  |  |  |  |  |  |  |  |  |  |  |  | 1 |
| Voss et al. (2011) | USA | x |  |  |  |  |  |  |  | x |  |  |  |  |  |  |  |  |  |  |  |  |  |  |  | x |  |  |  |  |  |  |  |  |  | 3 |
| Vuong et al. (2008) | Australia |  |  |  |  |  |  |  |  |  |  |  |  |  |  | x |  |  |  |  |  |  |  |  |  |  |  |  |  |  |  |  |  |  |  | 1 |
| Waggoner et al. (1981) | USA |  |  |  | x |  |  |  |  |  |  |  |  |  |  |  |  |  |  |  |  |  |  |  |  |  |  |  |  |  |  |  |  |  |  | 1 |
| Walker et al. (2009) | USA |  |  |  |  |  |  |  |  |  |  |  |  |  |  |  |  |  |  |  | x | x |  |  |  | x |  |  | x |  |  |  |  |  |  | 4 |
| Wallerstedt et al. (2012) | Sweden |  |  |  |  |  |  |  |  |  |  |  |  |  |  |  |  |  |  |  |  |  |  |  |  |  |  |  | x |  |  |  |  |  |  | 1 |
| Wang et al. (2020) | China |  |  |  |  |  |  |  |  |  |  |  |  |  |  |  |  |  |  |  |  |  |  |  | x |  |  |  |  |  |  |  |  |  |  | 1 |
| Warden et al. (2014) | USA |  |  |  |  |  |  |  |  |  |  |  |  |  |  |  |  |  |  |  | x |  |  |  |  |  |  |  |  |  |  |  |  |  |  | 1 |
| Webster et al. (2011) | Australia |  |  |  |  |  |  |  |  |  |  |  |  |  |  |  |  |  |  |  |  |  |  |  |  |  |  |  |  |  |  | x |  |  |  | 1 |
| Wee et al. (2014) | Singapore |  |  |  |  |  |  |  |  |  |  |  |  |  |  |  |  |  | x |  |  |  | x |  |  |  |  |  |  |  |  |  |  |  |  | 2 |
| Weinberger et al. (1996) | USA |  | x |  |  |  |  |  | x |  |  | x |  |  |  |  |  |  |  |  |  |  |  |  |  | x |  |  |  |  |  |  |  |  |  | 4 |
| Weiss et al. (2019) | USA |  |  |  |  |  |  |  |  |  |  |  |  |  |  |  |  |  |  |  |  |  |  |  |  |  |  |  |  |  |  | x |  |  |  | 1 |
| Wells et al. (2004) | UK |  |  |  |  |  |  |  |  |  |  |  |  |  |  | x |  |  |  |  |  |  |  |  |  |  |  |  |  |  |  |  |  |  |  | 1 |
| Wihelmson et al. (2017) | Sweden |  |  |  |  |  |  |  |  |  |  |  |  |  |  |  |  | x |  |  |  |  |  |  |  |  |  |  |  |  |  |  |  |  |  | 1 |
| Wilkin et al. (2020) | USA |  |  |  | x |  |  |  |  |  |  |  |  |  |  |  |  |  |  |  |  |  |  |  |  |  |  |  |  |  |  |  |  |  |  | 1 |
| Wilkinson et al. (2011) | USA |  |  |  |  |  |  |  |  |  |  |  |  |  |  |  |  |  |  |  | x |  |  |  |  | x |  |  |  |  |  |  |  |  |  | 2 |
| Williams et al. (2010) | UK |  |  |  |  |  |  |  |  |  |  |  |  |  |  |  |  |  |  |  |  |  |  |  |  |  |  |  |  | x |  |  |  |  |  | 1 |
| Wolff et al. (2010) | USA | x |  |  |  |  |  |  |  |  |  |  |  |  |  |  |  |  |  |  |  |  |  |  |  |  |  |  |  |  |  |  |  |  |  | 1 |
| Wong et al. (2004) | Hong Kong |  |  |  |  |  |  |  |  |  |  |  |  |  |  |  |  |  |  |  |  |  |  |  |  |  |  |  |  |  |  | x |  |  |  | 1 |
| Wong et al. (2008) | Hong Kong |  |  |  |  |  |  |  |  |  |  |  |  |  | x |  |  |  |  |  |  |  |  |  |  |  |  |  |  |  |  |  |  |  |  | 1 |
| Wong et al. (2011) | Hong Kong |  |  |  |  |  |  |  |  |  |  |  |  |  |  |  |  |  |  |  |  |  |  |  |  |  |  |  |  |  |  |  | x |  |  | 1 |
| Wong et al. (2014) | Hong Kong |  |  |  |  |  |  |  |  |  |  |  |  |  |  |  |  |  |  |  |  |  |  |  |  |  |  |  |  |  |  | x |  |  |  | 1 |
| Wong et al. (2015) | Hong Kong |  |  |  |  |  |  |  |  |  |  |  |  |  |  |  |  |  |  |  |  |  |  |  |  |  |  |  |  |  |  | x |  |  |  | 1 |
| Wong et al. (2016) | Hong Kong |  |  |  |  |  |  |  |  |  |  |  |  |  |  |  |  |  |  |  |  |  |  |  |  |  |  |  |  |  |  | x |  |  |  | 1 |
| Wright et al. (2019) | USA |  |  |  |  |  |  |  | x |  |  |  |  |  |  |  |  |  |  |  |  |  |  |  |  |  |  |  |  |  |  |  |  |  |  | 1 |
| Wu et al. (2019) | China |  |  |  |  |  | x |  |  |  |  |  |  |  |  |  |  |  |  |  |  |  |  |  |  |  |  |  |  |  |  | x |  |  |  | 2 |
| Wyer et al. (2015) | USA | x |  |  |  |  |  |  |  |  |  |  |  |  |  |  |  |  |  |  |  |  |  |  |  |  |  |  |  |  |  |  |  |  |  | 1 |
| Xiao et al. (2018) | China |  |  |  | x |  |  |  |  |  |  |  |  |  |  |  |  |  |  |  |  |  |  |  |  |  |  |  |  |  |  |  |  |  |  | 1 |
| Xie et al. (2018) | China |  |  |  |  |  |  |  |  |  |  |  |  |  |  |  |  |  |  |  |  |  |  |  |  |  |  |  |  |  |  | x |  |  |  | 1 |
| Xu et al. (2019) | China |  |  |  |  |  |  |  |  |  |  |  |  |  |  |  |  |  |  |  |  |  |  |  |  |  |  |  |  |  |  | x |  |  |  | 1 |
| Yiadom et al. (2020) | USA |  |  |  |  |  |  |  |  |  |  |  |  |  |  |  |  |  |  |  |  |  |  |  |  |  |  |  |  |  |  | x |  |  |  | 1 |
| Yin et al. (2020) | China |  |  |  | x |  |  |  |  |  |  |  |  |  |  |  |  |  |  |  |  |  |  |  |  |  |  |  |  |  |  | x |  |  |  | 2 |
| You et al. (2020) | China |  |  |  |  |  |  |  |  |  |  |  |  |  |  |  |  |  |  |  |  |  |  |  |  |  |  |  |  |  |  | x |  |  |  | 1 |
| Yu et al. (2015) | China |  |  |  |  |  |  |  |  |  |  |  |  |  |  |  |  |  |  | x |  |  |  |  |  |  |  |  |  |  |  |  |  |  |  | 1 |
| Yun et al. (2016) | Korea |  |  |  |  |  |  |  |  |  | x |  |  |  |  |  |  |  |  |  |  |  |  |  |  |  |  |  |  |  |  |  |  |  |  | 1 |
| Zhang et al. (2012) | China |  |  |  |  | x |  |  |  |  |  |  |  |  |  |  |  |  |  |  |  |  |  |  |  |  |  |  |  |  |  |  |  |  |  | 1 |
| Zhang et al. (2018) | China |  |  |  |  |  |  |  |  |  |  |  |  |  |  |  |  |  |  |  |  |  |  |  |  |  |  |  |  |  |  | x |  |  |  | 1 |
| Zhao et al. (2009) | China |  |  | x |  |  | x |  |  |  |  |  |  |  |  |  |  |  |  |  |  |  |  |  |  |  |  |  |  |  |  |  | x |  |  | 3 |

# **S10: Quality assessment according to AMSTAR2 for included reviews incl. alternative weighting**

|  | **1 - PICO** | **2 - Methods/Protocol** | **3 - Selection study designs** | **4 - Search strategy** | **5 - Duplicate study selection** | **6 - Duplicate data extraction** | **7 - Excluded studies** | **8 - Description included studies** | **9 - RoB** | **11 - Justification meta-analysis** | **12 - RoB in meta-analysis** | **13 - RoB individual studies** | **14- Heterogeneity** | **15 - Publication bias** | **16 - Conflict of interest** | **Quality score (Unweighted)** | **AMSTAR 2 Score (Unweighted)** |  | **Quality score (Weighting)** | **AMSTAR 2 Score (Weighting)** |
| --- | --- | --- | --- | --- | --- | --- | --- | --- | --- | --- | --- | --- | --- | --- | --- | --- | --- | --- | --- | --- |
| **Unweighted**  **Weighting** | 1 | 1 | 1 | 1 | 1 | 1 | 1 | 1 | 1 | 1 | 1 | 1 | 1 | 1 | 1 |  |  |  |  |  |
| **Weighting** | 2 | 2 | 1 | 3 | 2 | 2 | 3 | 3 | 3 | 3 | 3 | 3 | 3 | 3 | 1 |  |  |  |  |  |
|  |  |  |  |  |  |  |  |  |  |  |  |  |  |  |  |  |  |  |  |  |
| **Author** |  |  |  |  |  |  |  |  |  |  |  |  |  |  |  |  |  |  |  |  |
| **Albert et al. (2016)** | 0 | 0 | 0 | 0.5 | 0 | 0 | 0 | 0.5 | 0 | n.a. | n.a. | 0 | 0 | n.a. | 1 | Low | 0.17 |  | Low | 0.14 |
| **Allen et al. (2014)** | 1 | 0 | 0 | 0.5 | 1 | 0 | 1 | 0.5 | 1 | n.a. | n.a. | 0 | 0 | n.a. | 1 | Moderate | 0.50 |  | Moderate | 0.50 |
| **Backman et al. (2020)** | 1 | 0.5 | 0 | 0.5 | 1 | 1 | 1 | 0 | 1 | n.a. | n.a. | 0 | 1 | n.a. | 1 | Moderate | 0.67 |  | Moderate | 0.66 |
| **Becker et al. (2021)** | 0 | 0.5 | 0 | 0.5 | 1 | 1 | 0 | 0 | 1 | 0 | 1 | 0 | 0 | 1 | 1 | Moderate | 0.47 |  | Moderate | 0.45 |
| **Bonetti et al. (2020)** | 1 | 0.5 | 0 | 0.5 | 1 | 1 | 1 | 0 | 1 | 1 | 0 | 0 | 1 | 1 | 1 | Moderate | 0.67 |  | Moderate | 0.66 |
| **Chartrand et al. (2020)** | 1 | 1 | 0 | 0.5 | 0 | 1 | 1 | 0 | 1 | 1 | 0 | 0 | 1 | 0 | 1 | Moderate | 0.57 |  | Moderate | 0.55 |
| **Cheema et al. (2018)** | 1 | 0 | 1 | 0.5 | 0 | 1 | 0 | 0 | 1 | 1 | 0 | 0 | 1 | 0 | 0 | Moderate | 0.43 |  | Moderate | 0.42 |
| **Daliri et al. (2021)** | 1 | 1 | 0 | 1 | 1 | 1 | 1 | 1 | 1 | 1 | 1 | 1 | 1 | 0 | 1 | High | 0.87 |  | High | 0.89 |
| **Fønss Rasmussen et al. (2021)** | 1 | 1 | 0 | 1 | 1 | 1 | 1 | 1 | 1 | n.a. | n.a. | 1 | 0 | n.a. | 1 | High | 0.83 |  | High | 0.86 |
| **Gillespie et al. (2023)** | 1 | 1 | 0 | 0.5 | 1 | 1 | 1 | 1 | 1 | 1 | 0 | 1 | 1 | 0 | 1 | High | 0.77 |  | High | 0.77 |
| **Gonçalves-Bradley et al. (2022)** | 1 | 1 | 0 | 1 | 1 | 1 | 1 | 1 | 1 | 1 | 1 | 1 | 1 | 1 | 1 | High | 0.93 |  | High | 0.97 |
| **Gwadry-Sridhar et al. (2004)** | 1 | 0 | 0 | 0.5 | 1 | 0 | 0 | 0.5 | 0.5 | 1 | 1 | 1 | 0 | 1 | 1 | Moderate | 0.57 |  | Moderate | 0.58 |
| **Hammad et al. (2017)** | 0 | 0 | 0 | 1 | 1 | 1 | 0 | 0.5 | 1 | n.a. | n.a. | 1 | 1 | n.a. | 1 | Moderate | 0.63 |  | Moderate | 0.66 |
| **Hansen et al. (2011)** | 0 | 0 | 0 | 1 | 1 | 1 | 0 | 0 | 1 | n.a. | n.a. | 0 | 1 | n.a. | 1 | Moderate | 0.50 |  | Moderate | 0.50 |
| **Hesselink et al. (2012)** | 0 | 0 | 0 | 0.5 | 1 | 1 | 0 | 0.5 | 1 | n.a. | n.a. | 1 | 1 | n.a. | 1 | Moderate | 0.58 |  | Moderate | 0.61 |
| **Lambrinou et al. (2012)** | 0 | 0 | 0 | 0 | 0 | 1 | 0 | 0 | 0.5 | 1 | 0 | 0 | 1 | 1 | 1 | Moderate | 0.37 |  | Moderate | 0.36 |
| **Lee et al. (2022)** | 1 | 0.5 | 0 | 0.5 | 0 | 1 | 0 | 0.5 | 1 | 0 | 0 | 0 | 0 | 0 | 1 | Moderate | 0.37 |  | Low | 0.32 |
| **Leithaus et al. (2022)** | 1 | 0.5 | 0 | 0.5 | 0 | 1 | 0 | 0.5 | 0 | n.a. | n.a. | 0 | 0 | n.a. | 1 | Moderate | 0.38 |  | Low | 0.32 |
| **Mabire et al. (2018)** | 0 | 1 | 1 | 0.5 | 0 | 1 | 0 | 0.5 | 1 | 1 | 0 | 0 | 1 | 1 | 1 | Moderate | 0.60 |  | Moderate | 0.57 |
| **Mekonnen et al. (a) (2016)** | 0 | 0 | 0 | 1 | 0 | 0 | 1 | 0.5 | 1 | 1 | 0 | 0 | 1 | 1 | 1 | Moderate | 0.50 |  | Moderate | 0.55 |
| **Mekonnen et al. (b) (2016)** | 0 | 0 | 0 | 0.5 | 0 | 0 | 0 | 0.5 | 1 | 1 | 0 | 0 | 1 | 1 | 1 | Moderate | 0.40 |  | Moderate | 0.43 |
| **Meulenbroeks et al. (2021)** | 1 | 0 | 0 | 0.5 | 0 | 0 | 0 | 1 | 1 | n.a. | n.a. | 1 | 1 | n.a. | 1 | Moderate | 0.54 |  | Moderate | 0.59 |
| **Oh et al. (2023)** | 1 | 0.5 | 0 | 0 | 1 | 1 | 0 | 0.5 | 1 | 1 | 0 | 1 | 1 | 1 | 1 | Moderate | 0.67 |  | Moderate | 0.66 |
| **Park et al. (2023)** | 1 | 1 | 0 | 0,5 | 1 | 1 | 0 | 0,5 | 1 | 1 | 0 | 0 | 1 | 0 | 1 | Moderate | 0.60 |  | Moderate | 0.57 |
| **Rennke et al. (2013)** | 1 | 0 | 0 | 0.5 | 1 | 1 | 0 | 0.5 | 0 | n.a. | n.a. | 0 | 1 | n.a. | 1 | Moderate | 0.50 |  | Moderate | 0.46 |
| **Richards and Coast (2003)** | 1 | 0 | 1 | 0.5 | 0 | 0 | 1 | 0 | 1 | n.a. | n.a. | 0 | 1 | n.a. | 0 | Moderate | 0.46 |  | Moderate | 0.48 |
| **Rodakowski et al. (2017)** | 0 | 0 | 0 | 1 | 1 | 1 | 0 | 0 | 1 | 0 | 0 | 0 | 1 | 1 | 1 | Moderate | 0.47 |  | Moderate | 0.46 |
| **Skjøt-Arkil et al. (2018)** | 1 | 1 | 0 | 0.5 | 1 | 1 | 0 | 0.5 | 1 | n.a. | n.a. | 0 | 1 | n.a. | 1 | Moderate | 0.67 |  | Moderate | 0.64 |
| **Stamp et al. (2014)** | 0 | 0 | 0 | 0.5 | 0 | 0 | 0 | 0.5 | 0 | n.a. | n.a. | 0 | 1 | n.a. | 1 | Low | 0.25 |  | Low | 0.25 |
| **Tomlinson et al. (2020)** | 1 | 0.5 | 1 | 0.5 | 0 | 1 | 0 | 1 | 1 | 0 | 0 | 0 | 1 | 1 | 1 | Moderate | 0.60 |  | Moderate | 0.55 |
| **Tyler et al. (2023)** | 1 | 0.5 | 0 | 0.5 | 1 | 1 | 0 | 0.5 | 1 | 0 | 0 | 0 | 0 | 1 | 1 | Moderate | 0.50 |  | Moderate | 0.46 |
| **Verhaegh et al. (2014)** | 1 | 0 | 0 | 0.5 | 1 | 1 | 1 | 0 | 1 | 1 | 0 | 0 | 0 | 1 | 0 | Moderate | 0.50 |  | Moderate | 0.53 |
| **Villeneueve et al. (2021)** | 0 | 0 | 0 | 0.5 | 1 | 1 | 0 | 1 | 1 | n.a. | n.a. | 0 | 0 | n.a. | 1 | Moderate | 0.46 |  | Moderate | 0.45 |
| **Weeda et al. (2023)** | 1 | 0.5 | 0 | 0.5 | 1 | 1 | 0 | 0.5 | 1 | n.a. | n.a. | 0 | 1 | n.a. | 1 | Moderate | 0.63 |  | Moderate | 0.61 |

*Notes: Scores equal weighted averages; Due to minimal differences in results, the non-weighted approach was chosen*

***Assessment:***

- 0 = No
- 0,5 = Partial Yes
- 1 = Yes

***Categorization:***

- Low: Score < 0,33
- Moderate: 0,33 ≤ Score ≤ 0,67
- High: Score > 0,67

# **S11: Intervention Types included in each review**

|  |  |  |  | **Assessment & Plan Creation** | | | **Education** | | | | **Medication** | | | **Coordination** | | | | **Follow-up** | | | | **Other** | | |  |
| --- | --- | --- | --- | --- | --- | --- | --- | --- | --- | --- | --- | --- | --- | --- | --- | --- | --- | --- | --- | --- | --- | --- | --- | --- | --- |
|  | ***Author*** | ***# of primary studies*** | ***Review focus*** | **Home Assessment** | **Patient Assessment** | **Creation of Discharge Plan** | **Patient Education / Counselling/ Communication** | **Caregiver / Family Involvement / Education** | **Medication Reconciliation** | **Medication Review** | **Medication History** | **Provision of Medication Adherence Tools** | **Input from Outpatient Providers** | **Interdisciplinary Teamwork/ Meetings (in hospital)** | **Linkage to Outpatient Providers / Support Structures** | **Organizing Community Support** | **Scheduling Follow-up Appointments** | **Outpatient Follow-up (not further specified)** | **Post-discharge HC-professional Visit** | **Follow-up Home Visit** | **Follow-up Telephone Call** | **Hotline** | **Telemonitoring** | **DP Intervention (not further specified)** | **Total** |
| 1 | Albert et al. (2016) | 23 | Transitional Care/DP |  |  |  |  |  | x |  |  |  |  | x |  | x |  | x |  |  |  |  |  | x | **5** |
| 2 | Allen et al. (2014) | 12 | Transitional Care/DP |  |  |  | x |  | x |  |  |  |  |  | x |  |  | x |  |  |  |  |  | x | **5** |
| 3 | Backman et al. (2020) | 28 | PFCC |  |  |  | x | x | x |  |  |  |  | x |  |  |  |  | x |  |  |  |  | x | **6** |
| 4 | Becker et al. (2021) | 60 | Communication |  |  |  | x |  |  |  |  |  |  |  |  |  |  |  |  |  |  |  |  |  | **1** |
| 5 | Bonetti et al. (2020) | 21 | Medication |  |  |  | x |  | x | x |  |  |  |  | x |  |  | x |  |  | x |  |  |  | **6** |
| 6 | Chartrand et al. (2023) | 50 | PFCC |  |  |  | x | x | x |  |  |  |  | x |  | x |  | x |  |  |  |  |  | x | **7** |
| 7 | Cheema et al. (2018) | 18 | Medication |  |  | x | x | x | x | x | x | x |  | x | x |  |  | x |  |  | x |  |  |  | **11** |
| 8 | Daliri et al. (2021) | 15 | Medication |  |  |  | x |  | x | x |  |  |  |  | x |  |  |  |  |  |  |  |  |  | **4** |
| 9 | Fønss Rasmussen et al. (2021) | 11 | Transitional Care/DP | x | x | x | x | x | x |  |  |  |  | x | x | x |  |  |  |  | x |  |  | x | **11** |
| 10 | Gillespie et al. (2023) | 10 | Discharge Education |  |  |  | x |  |  |  |  |  |  |  |  |  |  |  |  | x |  |  |  |  | **2** |
| 11 | Gonçalves-Bradley et al. (2022) | 33 | Transitional Care/DP | x | x | x | x | x | x | x | x |  |  | x | x | x | x |  | x | x | x | x |  |  | **16** |
| 12 | Gwadry-Sridhar et al. (2004) | 8 | Heart failure Management Program |  |  |  | x | x |  |  |  |  |  |  |  |  |  |  |  | x | x |  |  |  | **4** |
| 13 | Hammad et al. (2017) | 13 | Medication |  |  |  | x | x | x | x |  |  |  |  | x |  |  |  |  |  |  |  |  | x | **6** |
| 14 | Hansen et al. (2011) | 43 | Interventions |  |  | x | x |  | x |  |  |  |  | x | x |  | x | x |  | x | x | x |  |  | **10** |
| 15 | Hesselink et al. (2012) | 36 | Interventions |  |  | x |  |  | x |  |  |  | x | x | x |  | x | x |  | x |  |  |  | x | **9** |
| 16 | Lambrinou et al. (2012) | 19 | Heart failure Management Program |  | x |  | x |  |  |  |  |  |  |  |  |  |  |  |  | x | x |  |  | x | **5** |
| 17 | Lee et al. (2022) | 21 | Transitional Care/DP | x |  |  |  | x |  | x |  |  |  | x |  |  |  |  |  | x | x |  |  | x | **7** |
| 18 | Leithaus et al. (2022) | 17 | Transitional Care/DP |  | x |  | x | x | x | x |  |  |  |  | x |  | x |  |  | x | x |  |  |  | **9** |
| 19 | Mabire et al. (2018) | 13 | Transitional Care/DP |  |  | x | x | x | x |  |  |  |  | x | x |  |  |  |  | x | x |  |  |  | **8** |
| 20 | Mekonnen et al. (a) (2016) | 17 | Medication |  |  | x | x |  | x | x | x | x | x |  | x |  | x | x |  | x | x |  |  |  | **12** |
| 21 | Mekonnen et al. (b) (2016) | 19 | Medication |  | x | x | x |  | x | x | x | x | x |  | x |  |  |  | x | x | x |  |  |  | **12** |
| 22 | Meulenbroeks et al. (2021) | 23 | PFCC |  |  |  |  | x |  |  |  |  |  | x | x |  |  |  |  |  |  |  |  |  | **3** |
| 23 | Oh et al. (2003) | 7 | Discharge Education |  |  |  | x |  |  |  |  |  |  |  |  |  |  |  |  |  |  |  |  |  | **1** |
| 24 | Park et al. (2023) | 9 | Transitional Care/DP |  | x | x | x |  |  |  |  |  |  |  |  |  |  |  | x | x | x |  |  |  | **6** |
| 25 | Rennke et al. (2013) | 47 | Transitional Care/DP |  | x |  | x | x | x |  |  |  |  | x | x |  |  |  |  | x | x |  |  |  | **8** |
| 26 | Richards and Coast (2003) | 23 | Interventions |  | x | x |  |  |  |  |  |  |  |  |  |  |  | x |  |  |  |  |  | x | **4** |
| 27 | Rodakowski et al. (2017) | 15 | PFCC |  |  |  | x | x | x |  |  |  |  |  | x |  |  |  |  |  |  |  |  |  | **4** |
| 28 | Skjøt-Arkil et al. (2018) | 28 | Medication |  | x |  | x |  | x | x | x |  |  | x | x |  |  |  |  |  | x |  |  |  | **8** |
| 29 | Stamp et al. (2014) | 20 | Transitional Care/DP |  |  |  | x | x |  | x |  |  |  |  | x |  | x |  |  | x | x |  |  |  | **7** |
| 30 | Tomlinson et al. (2020) | 24 | Transitional Care/DP |  |  | x | x |  | x | x |  |  |  | x | x |  |  |  |  | x | x | x |  |  | **9** |
| 31 | Tyler et al. (2023) | 126 | Transitional Care/DP |  | x | x | x | x | x | x |  |  | x | x | x |  |  |  |  |  | x | x | x |  | **12** |
| 32 | Verhaegh et al. (2014) | 26 | Transitional Care/DP |  | x |  | x | x |  |  |  |  |  |  | x |  |  |  |  | x | x |  |  | x | **7** |
| 33 | Villeneueve et al. (2021) | 17 | Medication |  |  |  | x |  | x |  |  |  |  | x |  |  |  | x |  |  |  |  |  |  | **4** |
| 34 | Weeda et al. (2023) | 11 | Medication |  |  |  | x |  | x |  | x | x |  |  | x |  | x |  |  | x | x |  |  |  | **8** |
| ***Total number of intervention types per review*** | | | | ***3*** | ***11*** | ***12*** | ***29*** | ***16*** | ***23*** | ***13*** | ***6*** | ***4*** | ***4*** | ***16*** | ***22*** | ***4*** | ***7*** | ***10*** | ***4*** | ***17*** | ***20*** | ***4*** | ***1*** | ***11*** |  |

# **S12: Sensitivity analyses for SoE with different weighting combinations**

| **#** | **Outcome** | **Baseline SoE calculation** | **Sensitivity analysis: Variation in thresholds (vs. baseline)** | | **Sensitivity analysis: Variation in  weightings (vs. baseline)** | | |
| --- | --- | --- | --- | --- | --- | --- | --- |
|  |  | As described in our Paper (chapter 2.3) | SoE-categorization: *“High”, if ≥ 60% (vs. 2/3) of literature reviews indicate significant findings  “Moderate”, if 30-59%*  *(vs. ≥ 1/3 and < 2/3) “Low”, if 0-29% (vs. < 1/3)* | Findings coded significant (Y=1): *For systematic reviews: if ≥ 60% (vs. 2/3) of primary studies cited in the review indicated a significant association between DP and the outcome* | No extra weight for ≥ 5 primary studies:  *For systematic reviews: aij=0 (vs. 1)*  *if ≥ 5 primary studies included* | No extra weight for results of meta-analysis: *For meta-analysis: aij=0 (vs. 1) if conducted* | No extra weight for high quality assessment: *Quality: aij=0 (vs. 1) for “high" methodological rating (AMSTAR 2)* |
| 1 | **Medication discrepancy** | High | High | High | High | High | High |
| 2 | **180-day readmission** | High | High | High | High | High | High |
| 3 | **Patient satisfaction** | High | High | High | High | High | High |
| 4 | **Readmission (mixed time frames)** | High | High | High | High | Moderate | High |
| 5 | **1-year readmission** | High | High | High | High | Moderate | High |
| 6 | **90-day readmission** | Moderate | Moderate | Moderate | Moderate | Low | Moderate |
| 7 | **Medication error** | Moderate | Moderate | Moderate | Moderate | Moderate | Moderate |
| 8 | **Patient knowledge** | Moderate | Moderate | Moderate | Moderate | Moderate | Moderate |
| 9 | **30-day readmission** | Moderate | Moderate | Moderate | Moderate | Low | Moderate |
| 10 | **Length of stay (LoS)** | Moderate | Moderate | Moderate | Moderate | Low | Low |
| 11 | **Discharge to home** | Moderate | Moderate | Moderate | Moderate | Moderate | Moderate |
| 12 | **Medication adherence** | Moderate | Moderate | Moderate | Moderate | Low | Moderate |
| 13 | **Revisits** | Moderate | Moderate | Moderate | Moderate | Moderate | Moderate |
| 14 | **Health/Functional status** | Low | Moderate | Moderate | Low | Low | Low |
| 15 | **Quality of life** | Low | Low | Low | Low | Low | Low |
| 16 | **Emergency department visits** | Low | Low | Low | Low | Low | Low |
| 17 | **Post-discharge healthcare utilization** | Low | Low | Moderate | Low | Low | Low |
| 18 | **Mortality** | Low | Low | Low | Low | Low | Low |
| 19 | **Adverse drug events (ADEs)** | Low | Low | Low | Low | Low | Low |

# **S13: SoE analysis (by SoE-subgroups)**

|  |  | **SoE (baseline)** | ***SoE by topic focus:*** | | | ***SoE by age group:*** | | ***SoE by disease group:*** | |
| --- | --- | --- | --- | --- | --- | --- | --- | --- | --- |
| **#** | **Outcome** |  | ***Transitional Care/ DP*** | ***Medication*** | ***PFCC*** | ***Adults*** | ***Older patients  (> 60 years)*** | ***Cardiological patients*** | ***other/ not specified*** |
| 1 | **Medication discrepancy** | **0,800** | n.a. | 1,000 | n.a. | 0,800 | 0,800 | n.a. | n.a. |
| 2 | **180-day readmission** | **0,769** | 0,889 | 0,000 | 0,667 | 0,571 | 0,571 | 0,000 | 0,000 |
| 3 | **Patient Satisfaction** | **0,714** | 1,000 | n.a. | 0,333 | 0,636 | 0,636 | n.a. | n.a. |
| 4 | **Readmission (mixed time Frames)** | **0,700** | 0,875 | 0,750 | 0,333 | 0,913 | 0,913 | 1,000 | 1,000 |
| 5 | **1-year readmission** | **0,667** | 0,667 | n.a. | n.a. | 1,000 | 1,000 | n.a. | n.a. |
| 6 | **90-day readmission** | **0,444** | 0,500 | 0,000 | 0,667 | 0,333 | 0,333 | 0,000 | 0,000 |
| 7 | **Medication error** | **0,400** | 0,250 | 1,000 | n.a. | 0,250 | 0,250 | n.a. | n.a. |
| 8 | **Patient knowledge** | **0,400** | 0,000 | n.a. | 1,000 | 0,400 | 0,400 | 0,000 | 0,000 |
| 9 | **30-day readmission** | **0,385** | 0,125 | 0,778 | 0,000 | 0,400 | 0,400 | 0,750 | 0,750 |
| 10 | **Medication adherence** | **0,375** | 0,000 | 0,333 | n.a. | 0,375 | 0,375 | 1,000 | 1,000 |
| 13 | **Length of Stay (LOS)** | **0,364** | 0,600 | 0,333 | 0,000 | 0,667 | 0,667 | n.a. | n.a. |
| 12 | **Discharge to home** | **0,333** | n.a. | n.a. | 1,000 | n.a. | n.a. | n.a. | n.a. |
| 11 | **Revisits** | **0,333** | n.a. | 0,333 | n.a. | 0,333 | 0,333 | n.a. | n.a. |
| 14 | **Health/Functional Status** | **0,300** | 0,750 | n.a. | 0,000 | 0,250 | 0,250 | 0,000 | 0,000 |
| 15 | **QoL** | **0,250** | 0,455 | 0,000 | 0,000 | 0,308 | 0,308 | 1,000 | 1,000 |
| 16 | **ED-visits** | **0,238** | 0,500 | 0,250 | 0,000 | 0,200 | 0,200 | 1,000 | 1,000 |
| 17 | **Post-discharge healthcare utilization** | **0,222** | 1,000 | 0,000 | 0,000 | 0,500 | 0,500 | 1,000 | 1,000 |
| 18 | **Mortality** | **0,000** | 0,000 | 0,000 | 0,000 | 0,000 | 0,000 | 0,000 | 0,000 |
| 19 | **Adverse drug events (ADEs)** | **0,000** | 0,000 | 0,000 | n.a. | 0,000 | 0,000 | n.a. | n.a. |

# **S14: Subgroup analysis – Methodological approach**

| **Methodological approach**  We categorized the included reviews based on how subgroup analyses were conducted:   1. **Inductive analyses of successful interventions (n=4):** Subgroup analyses were conducted post hoc and inductively to identify common characteristics of effective interventions 2. **Narrative or visual comparison of subgroup effects (n=10)**: Compared the share of significant effects or compared estimated effect sizes across subgroups narratively or visually 3. **Statistical testing of subgroup differences (n=6):** Subgroup differences were formally tested using statistical methods such as meta-regressions 4. **Mixed analyses for different outcomes (n=2):** Used a combination of approaches for analyzing different outcomes 5. **Performed or planned subgroup analysis but did not report comparative findings (n=4):** Reported results by subgroup (e.g., by intervention type) without concluding whether one type was more effective than another, or stated an intention to perform subgroup analysis but did not report or could not perform the analysis due to small numbers of studies    - The final group (n=5) was excluded from the presentation of subgroup analysis results as no comparative findings were reported |
| --- |

# **S15: Subgroup analysis (1/2)**

|  | | **Findings (# of reviews)** | | |  |
| --- | --- | --- | --- | --- | --- |
| **Subgroup types** | | *Differences identified^a^* | *No differences*  *identified* | *Mixed*  *findings* | **Total^b^** |
| *1* | **Intervention** **intensity**  *No. of interventions (components), intervention complexity/intensity, single vs. bundled/ multicomponent interventions* | 9 | 4 | 2 | **15 (17)** |
| *2* | **Intervention types/components** | 7 | 4 | 1 | **12 (13)** |
| *3* | **Professional group**  *No./type of professional delivering the intervention* | 2 | 4 | 0 | **6** |
| *4* | **Intervention timing**  *No. of stages/target stage of the intervention: pre-discharge, post-discharge, bridging, etc.* | 3 | 0 | 0 | **3** |
| *5* | **Country/region**  *e.g., US vs. Europe vs. other, Europe vs. Non-Europe* | 2 | 3 | 1 | **6** |
| *6* | **Medical conditions**  *e.g., diseases* | 2 | 1 | 0 | **3** |
| *7* | **Age** | - Older: 2 - Younger: 0 | 2 | 0 | **4** |
| *8* | **Gender**  *Higher share of males/females, balanced shares, or not reported* | - Male: 0 - Female: 0 | 2 | 0 | **2** |
| *9* | **Setting**  *e.g., hospitalized versus ED patients, single vs. multicenter setting* | 1 | 2 | 0 | **3** |
| *10* | **Patient complexity** | - Higher: 1 - Lower: 0 | 1 | 0 | **2** |
| *11* | **Study quality/risk of bias**  *low, moderate, high Quality/RoB* | - High Quality/ Low RoB: 0 - Low Quality/ High RoB: 1 | 1 | 1 | **3 (4)** |
| *12* | **Sample size (adequacy)**  *No. of patients included in study; power calculation performed* | - Smaller: 1 - Larger: 0 | 2 | 0 | **3** |
| *13* | **Study design**  *Randomized vs. non-randomized studies* | - Non-randomized: 1 - Randomized: 0 | 1 | 0 | **2** |
| *14* | **Alignment between purpose of an intervention and the outcome measured** | 1 | 0 | 0 | **1** |
| *15* | **Publication date** | 0 | 1 | 0 | **1** |

*^a^ e.g., younger/ older: Number of reviews which found significant differences for older/ younger patients*

*^b^ Number in brackets = Total number of subgroup analyses in this category; One review can conduct more than one subgroup analysis in one category (e.g., assessing differences between number of interventions and single vs. bundled interventions)*

# **S16: Subgroup analysis (2/2)**

|  | **Findings summarized narratively** |
| --- | --- |
| **Intervention and follow-up** | |
| **Intervention types/components** | Twelve reviews examined subgroup differences based on the type/components of discharge interventions. Four reviews did not identify any consistent patterns. The remaining eight reviews found that effective interventions emphasized patient-centered approaches, including clear discharge instructions, post-discharge follow-up calls, and medication reconciliation. Additional components associated with greater effectiveness included the use of electronic tools for structured discharge summaries and notifications, intensive follow-up care with home visits and telephone monitoring, and multidisciplinary, nurse-led education. Involving informal caregivers, promoting self-management, and ensuring communication between hospital and primary care providers also contributed to improved care continuity and patient outcomes. |
| **Intervention intensity**  *No. of interventions (components), intervention complexity/intensity, single vs. bundled/ multicomponent interventions* | In 15 subgroup analyses, 9 subgroup analyses in 7 reviews found that interventions with higher intensity or complexity (e.g., defined as including a greater number of interventions or intervention components or spanning more intervention categories) were more effective in improving outcomes. Four reviews did not find any clear differences, while two further reviews reported that the effects of intervention intensity varied depending on the specific outcomes assessed. |
| **Professional group**  *No./type of professional delivering the intervention* | Two reviews found non-significant or inconsistent effects related to which professional delivered the intervention. One review observed that the number of professionals involved was also not a determining factor. However, one review identified pharmacists as a crucial professional group contributing to effectiveness. Four reviews did not identify any consistent differences. |
| **Intervention timing**  *No. of stages/target stage of the intervention: pre-discharge, post-discharge, bridging, etc.* | Three reviews analyzed whether the number of stages and the target stage of interventions (e.g., pre-discharge, post-discharge, bridging) influenced the effectiveness of interventions. One review found that interventions delivered at multiple transitions were more effective, whereas two reviews found that interventions at single transitions were more effective. One of the latter two reviews concluded that the effect tended to stronger if the intervention was initiated at hospital admission, while the other found the strongest effects in interventions delivered after discharge. |
| **Study population and setting** | |
| **Country/Region**  *e.g., US vs. Europe vs. other, Europe vs. Non-Europe* | Six reviews analyzed whether the effectiveness of interventions differed by geographic region (e.g., US vs. Europe, Europe vs. non-Europe). However, comparisons were not possible across reviews and no clear pattern could be identified. |
| **Medical conditions**  *e.g., diseases* | Among three reviews investigating subgroups by patient medical conditions, findings were mixed. One review found greater effectiveness for patients with respiratory illnesses, whereas another reported stronger effects for those admitted to general internal medicine units. A third review did not observe significant differences in intervention effectiveness across diagnostic groups. |
| **Age** | Four reviews assessed variation by patient age. Two concluded that older adults benefited more from discharge interventions, whereas two others found no age-related differences in intervention effectiveness. |
| **Gender**  *Higher share of males/females, balanced shares, or not reported* | Two reviews examined whether intervention effectiveness varied by the gender composition of the study population (e.g., higher proportions of male or female patients, balanced samples). No consistent patterns were identified. |
| **Setting**  *e.g., hospitalized versus ED patients, single vs. multicenter setting* | Three reviews considered differences by care setting, such as hospitalized vs. ED patients or single- vs. multicenter sites. One review found that interventions were more effective for hospitalized patients than for those discharged from the ED. The other two other reviews, however, found no significant differences by setting type (single vs. multicenter) or location (hospital vs. community). However, comparisons were not possible across reviews. |
| **Patient complexity** | Two reviews explored whether intervention effectiveness differed by patient complexity or risk level. One concluded that patients at higher risk benefited more from discharge interventions, whereas the other review found no consistent pattern across complexity levels. |

| **Study design and quality** | |
| --- | --- |
| **Study Quality/risk of bias**  *low, moderate, high Quality/RoB* | Three reviews examined whether methodological quality (e.g., which was generally rated using the three categories of low, moderate or high) or risk of bias (which was generally rated as weak, moderate, strong) influenced the reported effectiveness of interventions. One review found no significant differences between studies with study quality or risk of bias rated as high vs. low. Another review reported larger effects in studies with a high risk of bias, whereas a third review found lower effectiveness only among studies rates as having moderate methodological quality. Overall, study quality and risk of bias do not appear to be important drivers of the reported effectiveness of discharge planning interventions. |
| **Sample size (adequacy)**  *No. of patients included in study; power calculation performed* | Three reviews explored whether sample size or the presence of a power calculation influenced estimates of effectiveness. One review concluded that studies with smaller sample sizes were associated with greater effectiveness. However, another found no such association. A third review compared subgroups of studies with and without formal power calculations and found no significant differences. |
| **Study design**  *Randomized vs. non-randomized studies* | Two reviews investigated whether study design (i.e., randomized vs. non-randomized) was associated with the effectiveness of interventions. Across several analyses, both reviews found no consistent differences in effect sizes by study design, although some exceptions suggested slightly greater effects in non-randomized studies. |
| **Other** | |
| **Alignment between the intended purpose of an intervention** (or a specific component of the intervention) **and the outcome measured** | One review examined whether intervention effectiveness varied based on the alignment between the intended purpose of an intervention (or a specific component of the intervention) and the outcome measured. It found that significant effects were more likely when the outcomes were directly related to this purpose (e.g., medication reconciliation was more effect in reducing the percentage of unreconciled medication after discharge). |
| **Publication date** | Regarding publication date (i.e., studies published before vs. after 2002), no consistent pattern in intervention effectiveness was identified in one review. |

*Note: For details of subgroup analysis results on the review-level, see Table S8*

# **S17: Subgroup analysis by intervention type**

|  |  |  |  |  |  |  |  | *Ass.* | *No ass.* | *Ass.* | *No ass.* | *Ass.* | *No ass.* | *Ass.* | *No ass.* | *Ass.* | *No ass.* | *Ass.* | *No ass.* | *Ass.* | *No ass.* | *Ass.* | *No ass.* | *Ass.* | *No ass.* | *Ass.* | *No ass.* | *Ass.* | *No ass.* | *Ass.* | *No ass.* | *Ass.* | *No ass.* | *Ass.* | *No ass.* | *Ass.* | *No ass.* | *Ass.* | *No ass.* | *Ass.* | *No ass.* |
| --- | --- | --- | --- | --- | --- | --- | --- | --- | --- | --- | --- | --- | --- | --- | --- | --- | --- | --- | --- | --- | --- | --- | --- | --- | --- | --- | --- | --- | --- | --- | --- | --- | --- | --- | --- | --- | --- | --- | --- | --- | --- |
| **Intervention type**  **(category)** | **Number of reviews mentioning intervention type** | **Number of reviews examining associations of intervention type** | **Number of reviews finding an**  **association** | **Number of reviews finding**  **no association** | **Sum of diff. outcomes investigated** | **of which finding associations** | **of which finding no associations** | **30-day readmission** | **30-day readmission** | **90-day readmission** | **90-day readmission** | **180-day readmission** | **180-day readmission** | **Readmission (various timeframes)** | **Readmission (various timeframes)** | **Mortality** | **Mortality** | **Medication discrepancies** | **Medication discrepancies** | **Medication adherence** | **Medication adherence** | **Medication error** | **Medication error** | **ADEs** | **ADEs** | **ED-visits** | **ED-visits** | **LOS** | **LOS** | **Patient satisfaction** | **Patient satisfaction** | **Patient knowledge** | **Patient knowledge** | **QoL** | **QoL** | **Discharge to home** | **Discharge to home** | **Revisits** | **Revisits** | **Post-discharge HC utilization** | **Post-discharge HC utilization** |
| **Preparation** |  |  |  |  |  |  |  |  |  |  |  |  |  |  |  |  |  |  |  |  |  |  |  |  |  |  |  |  |  |  |  |  |  |  |  |  |  |  |  |  |  |
| Creation of Discharge Plan | **12** | **2** | 2 | 0 | 1 | 1 | 0 | 0 | 0 | 0 | 0 | 0 | 0 | 1 | 0 | 0 | 0 | 0 | 0 | 0 | 0 | 0 | 0 | 0 | 0 | 0 | 0 | 0 | 0 | 0 | 0 | 0 | 0 | 0 | 0 | 0 | 0 | 0 | 0 | 0 | 0 |
| Patient Assessment | **11** | **2** | 2 | 0 | 1 | 1 | 0 | 0 | 0 | 0 | 0 | 0 | 0 | 1 | 0 | 0 | 0 | 0 | 0 | 0 | 0 | 0 | 0 | 0 | 0 | 0 | 0 | 0 | 0 | 0 | 0 | 0 | 0 | 0 | 0 | 0 | 0 | 0 | 0 | 0 | 0 |
| Home Assessment | **3** | **0** | 0 | 0 | 0 | 0 | 0 | 0 | 0 | 0 | 0 | 0 | 0 | 0 | 0 | 0 | 0 | 0 | 0 | 0 | 0 | 0 | 0 | 0 | 0 | 0 | 0 | 0 | 0 | 0 | 0 | 0 | 0 | 0 | 0 | 0 | 0 | 0 | 0 | 0 | 0 |
| **Education** |  |  |  |  |  |  |  |  |  |  |  |  |  |  |  |  |  |  |  |  |  |  |  |  |  |  |  |  |  |  |  |  |  |  |  |  |  |  |  |  |  |
| Patient Education / Counselling / Communication | **29** | **6** | 4 | 2 | 14 | 6 | 8 | 2 | 2 | 0 | 0 | 0 | 0 | 2 | 1 | 0 | 1 | 0 | 0 | 1 | 0 | 0 | 0 | 0 | 0 | 0 | 1 | 0 | 0 | 1 | 1 | 0 | 1 | 0 | 1 | 0 | 0 | 0 | 0 | 0 | 0 |
| Caregiver / Family Involvement / Education | **16** | **2** | 2 | 0 | 2 | 2 | 0 | 0 | 0 | 0 | 0 | 0 | 0 | 2 | 0 | 0 | 0 | 0 | 0 | 0 | 0 | 0 | 0 | 0 | 0 | 0 | 0 | 0 | 0 | 0 | 0 | 0 | 0 | 0 | 0 | 0 | 0 | 0 | 0 | 0 | 0 |
| **Medication** |  |  |  |  |  |  |  |  |  |  |  |  |  |  |  |  |  |  |  |  |  |  |  |  |  |  |  |  |  |  |  |  |  |  |  |  |  |  |  |  |  |
| Medication Reconciliation | **23** | **5** | 3 | 2 | 12 | 7 | 5 | 1 | 0 | 0 | 0 | 0 | 0 | 2 | 0 | 0 | 2 | 2 | 0 | 0 | 0 | 1 | 0 | 0 | 1 | 1 | 0 | 0 | 1 | 0 | 0 | 0 | 0 | 0 | 0 | 0 | 0 | 0 | 1 | 0 | 0 |
| Medication Review | **13** | **0** | 0 | 0 | 0 | 0 | 0 | 0 | 0 | 0 | 0 | 0 | 0 | 0 | 0 | 0 | 0 | 0 | 0 | 0 | 0 | 0 | 0 | 0 | 0 | 0 | 0 | 0 | 0 | 0 | 0 | 0 | 0 | 0 | 0 | 0 | 0 | 0 | 0 | 0 | 0 |
| Medication History | **6** | **0** | 0 | 0 | 0 | 0 | 0 | 0 | 0 | 0 | 0 | 0 | 0 | 0 | 0 | 0 | 0 | 0 | 0 | 0 | 0 | 0 | 0 | 0 | 0 | 0 | 0 | 0 | 0 | 0 | 0 | 0 | 0 | 0 | 0 | 0 | 0 | 0 | 0 | 0 | 0 |
| Provision of Medication Adherence Tools | **4** | **0** | 0 | 0 | 0 | 0 | 0 | 0 | 0 | 0 | 0 | 0 | 0 | 0 | 0 | 0 | 0 | 0 | 0 | 0 | 0 | 0 | 0 | 0 | 0 | 0 | 0 | 0 | 0 | 0 | 0 | 0 | 0 | 0 | 0 | 0 | 0 | 0 | 0 | 0 | 0 |
| **Coordination** |  |  |  |  |  |  |  |  |  |  |  |  |  |  |  |  |  |  |  |  |  |  |  |  |  |  |  |  |  |  |  |  |  |  |  |  |  |  |  |  |  |
| Linkage to Outpatient Providers / Support Structures | **22** | **3** | 2 | 1 | 2 | 1 | 1 | 1 | 0 | 0 | 0 | 0 | 0 | 0 | 1 | 0 | 0 | 0 | 0 | 0 | 0 | 0 | 0 | 0 | 0 | 0 | 0 | 0 | 0 | 0 | 0 | 0 | 0 | 0 | 0 | 0 | 0 | 0 | 0 | 0 | 0 |
| Interdisciplinary Teamwork/ Meetings (in hospital) | **16** | **0** | 0 | 0 | 0 | 0 | 0 | 0 | 0 | 0 | 0 | 0 | 0 | 0 | 0 | 0 | 0 | 0 | 0 | 0 | 0 | 0 | 0 | 0 | 0 | 0 | 0 | 0 | 0 | 0 | 0 | 0 | 0 | 0 | 0 | 0 | 0 | 0 | 0 | 0 | 0 |
| Scheduling Follow-up Appointments | **7** | **0** | 0 | 0 | 0 | 0 | 0 | 0 | 0 | 0 | 0 | 0 | 0 | 0 | 0 | 0 | 0 | 0 | 0 | 0 | 0 | 0 | 0 | 0 | 0 | 0 | 0 | 0 | 0 | 0 | 0 | 0 | 0 | 0 | 0 | 0 | 0 | 0 | 0 | 0 | 0 |
| Input from Outpatient Providers | **4** | **0** | 0 | 0 | 0 | 0 | 0 | 0 | 0 | 0 | 0 | 0 | 0 | 0 | 0 | 0 | 0 | 0 | 0 | 0 | 0 | 0 | 0 | 0 | 0 | 0 | 0 | 0 | 0 | 0 | 0 | 0 | 0 | 0 | 0 | 0 | 0 | 0 | 0 | 0 | 0 |
| Organizing Community Support | **4** | **0** | 0 | 0 | 0 | 0 | 0 | 0 | 0 | 0 | 0 | 0 | 0 | 0 | 0 | 0 | 0 | 0 | 0 | 0 | 0 | 0 | 0 | 0 | 0 | 0 | 0 | 0 | 0 | 0 | 0 | 0 | 0 | 0 | 0 | 0 | 0 | 0 | 0 | 0 | 0 |
| **Follow-up** |  |  |  |  |  |  |  |  |  |  |  |  |  |  |  |  |  |  |  |  |  |  |  |  |  |  |  |  |  |  |  |  |  |  |  |  |  |  |  |  |  |
| Follow-up Telephone Call | **20** | **6** | 6 | 0 | 6 | 6 | 0 | 1 | 0 | 0 | 0 | 0 | 0 | 4 | 0 | 1 | 0 | 0 | 0 | 0 | 0 | 0 | 0 | 0 | 0 | 0 | 0 | 0 | 0 | 0 | 0 | 0 | 0 | 0 | 0 | 0 | 0 | 0 | 0 | 0 | 0 |
| Follow-up Home Visit | **17** | **6** | 6 | 0 | 6 | 6 | 0 | 1 | 0 | 0 | 0 | 0 | 0 | 4 | 0 | 1 | 0 | 0 | 0 | 0 | 0 | 0 | 0 | 0 | 0 | 0 | 0 | 0 | 0 | 0 | 0 | 0 | 0 | 0 | 0 | 0 | 0 | 0 | 0 | 0 | 0 |
| Outpatient Follow-up (not further specified) | **10** | **0** | 0 | 0 | 0 | 0 | 0 | 0 | 0 | 0 | 0 | 0 | 0 | 0 | 0 | 0 | 0 | 0 | 0 | 0 | 0 | 0 | 0 | 0 | 0 | 0 | 0 | 0 | 0 | 0 | 0 | 0 | 0 | 0 | 0 | 0 | 0 | 0 | 0 | 0 | 0 |
| Post-discharge HC-professional Visit | **4** | **0** | 0 | 0 | 0 | 0 | 0 | 0 | 0 | 0 | 0 | 0 | 0 | 0 | 0 | 0 | 0 | 0 | 0 | 0 | 0 | 0 | 0 | 0 | 0 | 0 | 0 | 0 | 0 | 0 | 0 | 0 | 0 | 0 | 0 | 0 | 0 | 0 | 0 | 0 | 0 |
| Hotline | **4** | **0** | 0 | 0 | 0 | 0 | 0 | 0 | 0 | 0 | 0 | 0 | 0 | 0 | 0 | 0 | 0 | 0 | 0 | 0 | 0 | 0 | 0 | 0 | 0 | 0 | 0 | 0 | 0 | 0 | 0 | 0 | 0 | 0 | 0 | 0 | 0 | 0 | 0 | 0 | 0 |
| Telemonitoring | **1** | **0** | 0 | 0 | 0 | 0 | 0 | 0 | 0 | 0 | 0 | 0 | 0 | 0 | 0 | 0 | 0 | 0 | 0 | 0 | 0 | 0 | 0 | 0 | 0 | 0 | 0 | 0 | 0 | 0 | 0 | 0 | 0 | 0 | 0 | 0 | 0 | 0 | 0 | 0 | 0 |
| **DP interventions** *(not further specified)* | **11** | **2** | 2 | 0 | 0 | 0 | 0 | 0 | 0 | 0 | 0 | 0 | 0 | 0 | 0 | 0 | 0 | 0 | 0 | 0 | 0 | 0 | 0 | 0 | 0 | 0 | 0 | 0 | 0 | 0 | 0 | 0 | 0 | 0 | 0 | 0 | 0 | 0 | 0 | 0 | 0 |
| **Sum** |  |  | **29** | **5** | **44** | **30** | **14** | **6** | **2** | **0** | **0** | **0** | **0** | **16** | **2** | **2** | **3** | **2** | **0** | **1** | **0** | **1** | **0** | **0** | **1** | **1** | **1** | **0** | **1** | **1** | **1** | **0** | **1** | **0** | **1** | **0** | **0** | **0** | **1** | **0** | **0** |

Note: This analysis includes both intervention types assessed through subgroup analyses (see S14–16) and those examined in reviews that focused on a single type (e.g., education as in *Gillespie et al., 2023*) without conducting a dedicated subgroup analysis.

The columns “Number of reviews finding an association/no associations” summarize reviews that discuss associations between outcomes and single intervention types overall, regardless of specific outcomes.

The green and white columns (from “30-day readmissions” onwards to the right) capture reviews reporting associations of intervention types with particular outcomes, finding either an association or no association. Since one review can discuss the effect of one intervention type on either none, one, or several outcomes, the sum across all outcome-columns does not necessarily match the total in “Number of reviews examining associations of intervention types”.

Results on the association with readmissions are summarized in the paper in Figure 3.
